# Hepatocyte-intrinsic SMN deficiency drives metabolic dysfunction and liver steatosis in spinal muscular atrophy

**DOI:** 10.1172/JCI173702

**Published:** 2024-05-09

**Authors:** Damien Meng-Kiat Leow, Yang Kai Ng, Loo Chien Wang, Hiromi W.L. Koh, Tianyun Zhao, Zi Jian Khong, Tommaso Tabaglio, Gunaseelan Narayanan, Richard M. Giadone, Radoslaw M. Sobota, Shi-Yan Ng, Adrian Kee Keong Teo, Simon H. Parson, Lee L. Rubin, Wei-Yi Ong, Basil T. Darras, Crystal J.J. Yeo

**Affiliations:** 1 Yong Loo Lin School of Medicine, National University of Singapore, Singapore, Singapore.; 2 Institute of Molecular and Cell Biology (IMCB), Agency for Science, Technology and Research (A*STAR), 61 Biopolis Drive, Proteos, Singapore, Singapore.; 3 Duke-NUS Medical School, Singapore, Singapore.; 4 Department of Stem Cell and Regenerative Biology, Harvard University, Cambridge Massachusetts, USA.; 5 National Neuroscience Institute, Singapore, Singapore.; 6 Institute of Education in Healthcare and Medical Sciences, School of Medicine, Medical Sciences and Nutrition, University of Aberdeen, Aberdeen, Scotland.; 7 Department of Neurology, Boston Children’s Hospital, Harvard Medical School, Boston, Massachusetts, USA.; 8 Department of Neurology, Feinberg School of Medicine, Northwestern University, Chicago, Illinois, USA.; 9 Lee Kong Chian School of Medicine, Nanyang Technological University, Singapore, Singapore.

**Keywords:** Metabolism, Neuroscience, Neuromuscular disease

## Abstract

Spinal muscular atrophy (SMA) is typically characterized as a motor neuron disease, but extraneuronal phenotypes are present in almost every organ in severely affected patients and animal models. Extraneuronal phenotypes were previously underappreciated, as patients with severe SMA phenotypes usually died in infancy; however, with current treatments for motor neurons increasing patient lifespan, impaired function of peripheral organs may develop into significant future comorbidities and lead to new treatment-modified phenotypes. Fatty liver is seen in SMA animal models, but generalizability to patients and whether this is due to hepatocyte-intrinsic survival motor neuron (SMN) protein deficiency and/or subsequent to skeletal muscle denervation is unknown. If liver pathology in SMA is SMN dependent and hepatocyte intrinsic, this suggests SMN-repleting therapies must target extraneuronal tissues and motor neurons for optimal patient outcome. Here, we showed that fatty liver is present in SMA patients and that SMA patient–specific induced pluripotent stem cell–derived hepatocyte-like cells were susceptible to steatosis. Using proteomics, functional studies, and CRISPR/Cas9 gene editing, we confirmed that fatty liver in SMA is a primary SMN-dependent hepatocyte-intrinsic liver defect associated with mitochondrial and other hepatic metabolism implications. These pathologies require monitoring and indicate the need for systematic clinical surveillance and additional and/or combinatorial therapies to ensure continued SMA patient health.

## Abstract 2

A hepatocyte cell–autonomous defect, secondary to SMN deficiency, drives fatty liver in spinal muscular atrophy, suggesting that current SMN-repleting therapies are insufficient.

## Graphical abstract

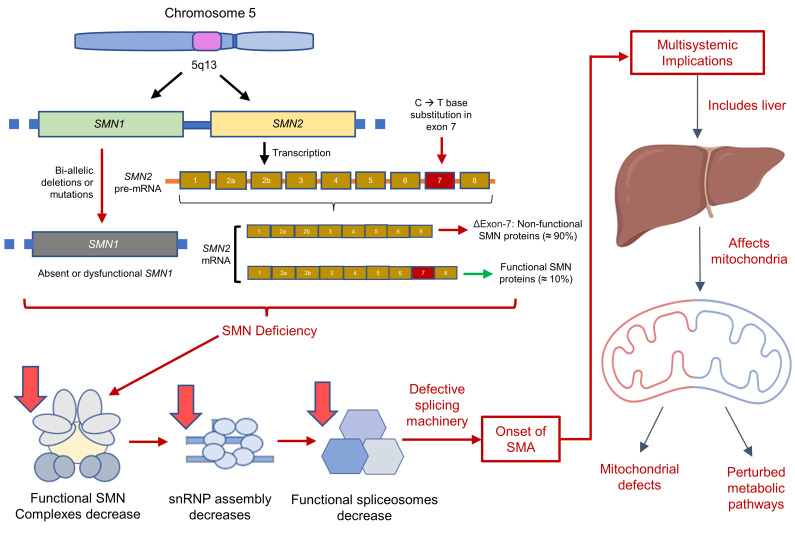

## Introduction

Spinal muscular atrophy (SMA) is one of the most common monogenetic autosomal recessive neuromuscular genetic diseases, with a worldwide carrier frequency of approximately 1 in 52 and disease incidence of around 1 in 10,000 (1). SMA clinical phenotypes are heterogeneous, ranging from infancy to adulthood, and classified into 5 major types based on age at onset of symptoms and maximum level of motor function achieved (2–6), with the most severe being SMA types 0 and 1 (SMA0 and -1), and mildest being SMA3–SMA4. The disease is caused by biallelic deletions in survival motor neuron 1 (*SMN1*) genes (7), resulting in a scarcity of survival motor neuron (SMN) protein and death of motor neurons. One to more than 4 copies of the *SMN2* gene, a highly homologous version of *SMN1*, are present in 95% of humans (8), and a c.840 C>T transition in exon 7, which converts an exonic splicing enhancer into an exonic splicing silencer, causes aberrant splicing and production of mostly truncated SMN protein (9). Fewer *SMN2* copies and lower amounts of SMN protein are associated with the most severe phenotypes (SMA0 and SMA1).

Before the US Food and Drug Administration’s (FDA’s)approval of the antisense oligonucleotide nusinersen in 2016 (10), adeno-associated virus serotype 9–mediated *SMN1* gene replacement therapy onasemnogene abeparvovec-xioi in 2019 (11), and small molecule splicing *SMN2* modifier risdiplam in 2020 (12), SMA was invariably fatal in infancy for the most severe forms of SMA. With recent innovative advances in the therapeutic landscape, infants and children below the age of 2 with SMA1 can now survive to childhood (10, 13) and are expected to survive into adulthood. It is thought that they are unlikely to achieve full motor capacity, as these new therapeutics are not outright cures. These therapeutics can only increase full-length functional SMN protein expression to halt the progression of disease by preventing future motor neuron loss, but cannot reverse motor neuron loss that has already occurred. Additionally, data are still emerging concerning treatment tolerability, efficacy, and durability in different clinical settings and age groups, and the long-term adverse effects of treatment are unclear (14).

While SMA is typically characterized as a motor neuron disease, extraneuronal phenotypes have been noted in severely affected patients and animal models (15). Data from autopsies, case studies, and limited cohort studies in patients with severe SMA correspond to animal models of SMA, showing dysfunction in almost every peripheral organ system, including the skeletal muscle, heart, kidney, liver, pancreas, spleen, bone, connective tissues, and immune systems, providing evidence in support of SMA as a multisystem disorder (16–19). SMN protein is ubiquitously expressed in all tissues and has roles in ribonucleoprotein components, RNA transcription and splicing (20), ribosomal function, and messenger RNA translation; however, the complete range of functions of SMN protein in all tissues is unknown (21, 22). Extraneuronal phenotypes were not a focus in the past, as patients with severe phenotypes of SMA usually died by the age of 2 years, but with current treatments that increase survival and lifespans of SMA patients, impaired function of peripheral tissues and organs may become significant future comorbidities and cause the emergence of treatment-modified phenotypes, particularly for treatments that only target motor neurons (14). It is vital to understand extraneuronal phenotypes to inform systematic clinical surveillance and treatment programs. Whether extraneuronal phenotypes are caused by cell-intrinsic SMN protein deficiency and/or are secondary to motor neuron death needs to be explored, as it changes patient management. In the former, SMN-repleting therapies must target extraneuronal tissues as well as motor neurons for optimal treatment outcome; in the latter, treatment of motor neurons would suffice.

The liver is an important organ to study in SMA because of its many functions in detoxification, metabolism, immune regulation, hematopoiesis, and clotting (23), and its implications for SMA treatments. Onasemnogene abeparvovec-xio is usually associated with mild to moderate hepatotoxicity within days to a few weeks of treatment (13). Notably, 2 children with SMA died of acute liver failure 6–7 weeks after receiving gene therapy in August 2022, suggesting an urgent need to identify patients who react poorly to treatment and to understand the mechanisms underlying liver damage in SMA (24). Risdiplam is primarily metabolized by hepatic flavin monooxygenases 1 and 3 (FMO1 and FMO3) (25), and changes in pharmacokinetics may affect drug efficacy, durability, and toxicity. Over 5,000 hepatic genes are dysregulated with disease progression in mouse models of SMA, as compared with 1,000 genes in muscle, suggesting that despite SMA being classified as a neuromuscular disease, pathways are also significantly dysregulated in the liver (26). Targeted hepatic ablation of SMN is embryonically lethal (27) and peripheral depletion of SMN in adult mice results in lipid accumulation, altered iron homeostasis (28), hepatocellular necrosis, and inflammatory infiltrates (29). Animal models of SMA have fatty liver, but it is not known whether this is primarily due to hepatic SMN deficiency or secondary to metabolic changes subsequent to skeletal muscle denervation (30, 31). The etiology, importance, and generalizability of these findings to patients with SMA remain unclear; although one small study showed hepatic steatosis in a proportion of necropsies of patients diagnosed with SMA, most of the diagnoses were obtained through clinical examination and histology prior to the era of genetic testing (30).

The primary goal of this study was to describe hepatic defects in SMA patients and cell-intrinsic defects in SMA patient–derived induced pluripotent stem cell–derived (iPSC-derived) hepatocyte-like cells (iHeps). We retrospectively analyzed clinical data from a single-center cohort of pediatric and adult SMA patients without liver disease who received hepatic sonography. Sonographic steatosis grade and serum markers of liver function were reviewed as potential clinical biomarkers of liver dysfunction. In parallel, we differentiated type 0 to type 3 SMA patient iPSCs into iHeps to evaluate SMA hepatocyte–intrinsic effects. Lipid accumulation and proteomics were analyzed. We CRISPR edited a c.840 T>C transition in exon 7 of *SMN2* to generate *SMN1*-like genes with full-length SMN protein expression in an SMA1 iPSC line to rescue SMN protein expression on an isogenic background and confirm SMN-dependent effects. This work demonstrates that SMA patient hepatic steatosis is a cell-intrinsic metabolic phenotype, and highlights the critical need for further investigation of the liver in SMA and its clinical implications.

## Results

### SMA patients display liver steatosis.

Liver pathology, and specifically fatty liver (steatosis), have been described in mouse models of SMA, but patient data in this area are scant. To examine whether steatosis is present in the patient population, we carried out a retrospective, single-center cohort study on pediatric and adult patients with SMA1–SMA3 who had undergone liver ultrasound and collection of clinical serum markers of liver function. All patients were on risdiplam, nusinersen, or had received gene therapy. Six out of 8 (75%) of these SMA patients had ultrasonic evidence of increased hepatic echogenicity consistent with mild to moderate hepatic steatosis. Three out of 8 (37.5%) also had changes in serum markers indicative of liver damage, namely increases in alanine transaminase (ALT), aspartate transaminase (AST), and gamma glutamyl transferase (GGT) (Table 1). One of the patients who had elevated GGT without increased hepatic echogenicity was taking phenobarbital, a drug that can cause hepatocellular damage. Two adults with SMA had possible non-SMA causes of liver dysfunction: obesity and heavy alcohol use. None had changes in clinical serum markers of protein synthetic function (albumin, protein, bilirubin) and only the patient with obesity had impaired fasting glucose suggestive of insulin resistance. Interestingly, we found that hepatic steatosis was detected in patients across all SMA genotypes examined, regardless of severity of the neuromuscular phenotype. Increased hepatic echogenicity therefore has potential as a noninvasive and sensitive biomarker for a range of SMA types, although care should be taken to exclude other causes of liver dysfunction. Interestingly, steatosis was present in treated patients, suggesting that current therapies may not be sufficient to target this phenotype for these patients at the time of treatment.

### SMA iHeps show increased lipid accumulation.

To determine whether the pathological liver phenotype seen in SMA patients was hepatocyte intrinsic, we created an in vitro hepatocyte model. To do this, we differentiated SMA and non-SMA (WT) human iPSCs (hiPSCs) into iHeps (32, 33) as an in vitro model of the SMA liver phenotype (Supplemental Figure 1; supplemental material available online with this article; https://doi.org/10.1172/JCI173702DS1). SMA iPSCs were obtained from untreated SMA patients. Day 24 iHeps displayed large angular polygonal morphology, distinct round nuclei with 1 or 2 prominent nucleoli, and binucleate cells with bright junctions, characteristic of mature hepatocytes (Supplemental Figure 1). Quality control for RT-qPCR primers and house-keeping genes were performed prior to gene analysis (Supplemental Figure 2). RT-qPCR showed expression of a range of liver-specific genes: albumin (*ALB*), asialoglycoprotein receptor 1 (*ASGR1*) and 2 (*ASGR2*), apolipoprotein E (*APOE*), apolipoprotein A1 (*APOA1*), prothrombin (*F2*), α1-antitrypsin (*SERPINA1*), haptoglobin (*HP*), hepatocyte nuclear factor 4α (*HNF4A*), and α-fetoprotein (*AFP*), representing liver-specific functions of serum protein secretion and homeostasis, lipid metabolism, clotting, and hemolysis (Supplemental Figure 3A and Supplemental Figure 4A). Conversely, gene expression of embryonic stem cell markers POU class 5 homeobox 1 (*OCT4*), nanog homeobox (*NANOG*), and SRY-box transcription factor 2 (*SOX2*) was negligible(Supplemental Figure 3B and Supplemental Figure 4B). The iHeps were determined to be functional, as we found similar levels of urea secretion in cultures of WT and SMA1 and SMA3 iHeps (Supplemental Figure 3C).

**Figure 1:**
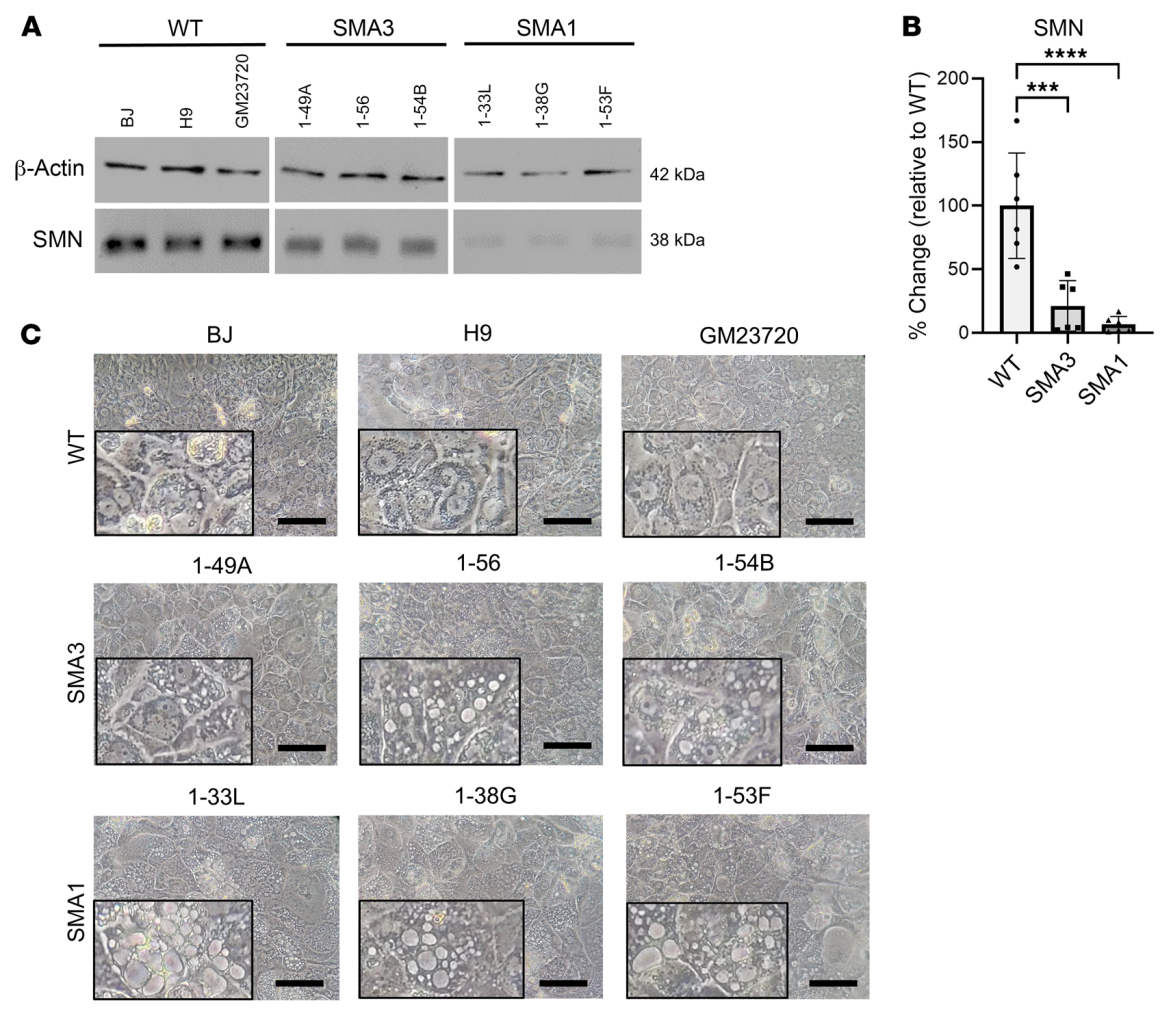
Day 24 SMA iHeps showed larger vacuoles. (**A**) Protein quantification of SMN expression between day 24 WT and SMA iHeps by Western blot, with β-actin as the housekeeping protein for normalization for ImageJ analysis (**B**). Data are from 2 independent experiments, each with 3 biological replicates (WT *n* = 6, SMA1 *n* = 6, SMA3 *n* = 6). (**C**) Bright-field microscopy images of day 24 WT and SMA iHeps. Boxed portions represent zoomed-in segments of the original image to showcase vacuole enlargement more clearly. Scale bars: 50 μm and 25 μm (zoomed-in images). Data are presented as mean ± SD. ***P < 0.001, ****P < 0.0001 by 1-way ANOVA with Tukey’s multiple-comparison test.

**Figure 2:**
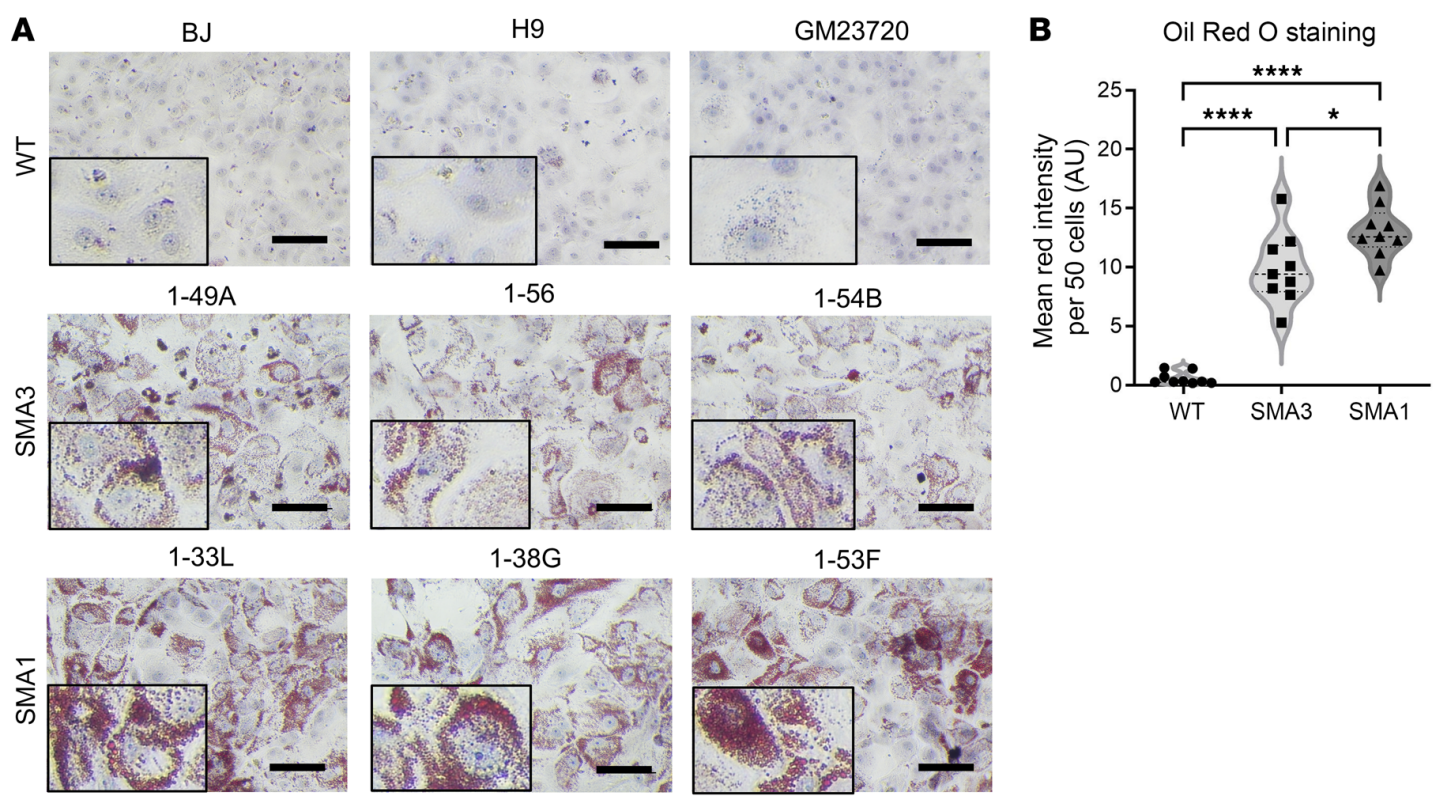
Day 24 SMA iHeps showed increased lipid accumulation. (**A**) Oil Red O (ORO) staining of day 24 WT and SMA iHeps and (**B**) ImageJ analysis. Hematoxylin stains nuclear components and ORO stains neutral triglycerides and lipids. Boxed portions represent zoomed-in segments of the original image to showcase ORO staining of triglycerides and lipids with more clarity. Scale bars: 50 μm and 25 μm (zoomed-in images). In **B**, the mean red intensity of 50 cells is presented. Data are representative of 3 independent experiments, each with 3 biological replicates (WT *n* = 9, SMA1 *n* = 9, SMA3 *n* = 9), and are presented as mean ± SD. *P < 0.05; ****P < 0.0001 by 1-way ANOVA with Tukey’s multiple-comparison test.

Consistent with SMA pathology, SMA iHeps showed markedly reduced SMN protein expression on Western blot and image analysis (Figure 1, A and B). Patient-derived SMA iHeps had increased numbers of small and large vacuoles (Figure 1C) that stained with Oil Red O (ORO), the gold standard histochemical stain for identifying lipids (Figure 2A). Using ORO and image analysis to quantify steatosis (34), SMA iHeps showed a 10-fold increase in lipid accumulation compared with WT (Figure 2B). Mean ORO staining intensity per 50 cells in each line is presented in Supplemental Figure 5A. Normalization of mean ORO staining intensity to cell number is presented in Supplemental Figure 5C. Lipid accumulation was similar across mild (SMA3) and severe (SMA1) clinical phenotypes, which showed that lipid accumulation is hepatocyte intrinsic and present across a range of untreated SMA severities.

### Distinct proteome changes are present in SMA iHeps.

**Figure 3:**
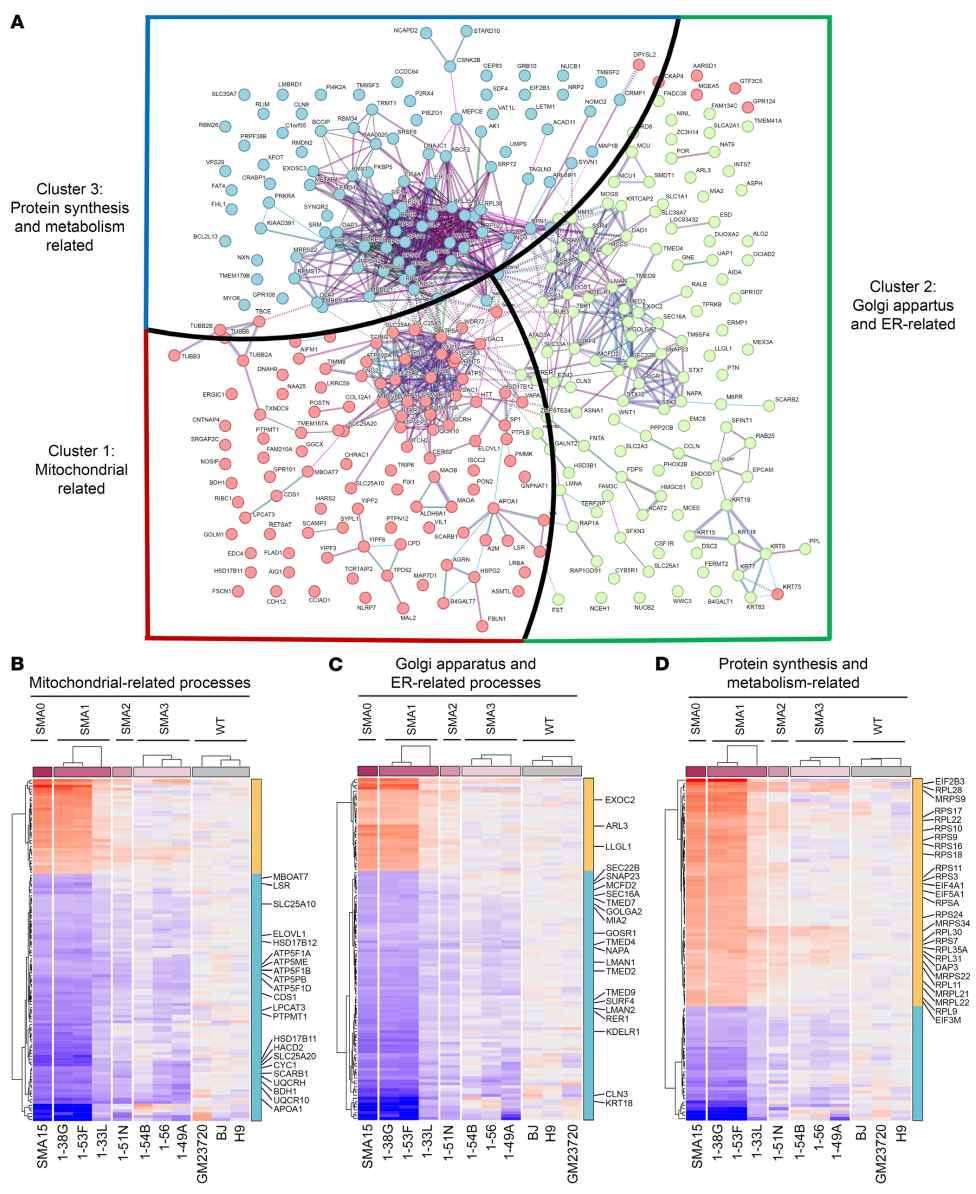
Proteomics analysis of day 24 WT and SMA iHeps. (**A**) Three hundred and forty-three proteins that showed significant differences in SMA patient–derived iHep cell lines compared with WT patient cell lines were analyzed for their interactions on the STRING database. Proteins in individual clusters were subjected to term enrichment analysis using the ShinyGO app (see Supplemental Methods). Cluster 1 (red), cluster 2 (green), and cluster 3 (blue) suggested that there were mitochondria-related processes, enrichment of Golgi apparatus– and ER-related processes, and henrichment of protein synthesis– and metabolism–related processes in the interaction network using the UniProt database (see Supplemental Methods). (**B**–**D**) Heatmap demonstration of significantly regulated proteins between SMA and WT iHeps, with proteins implicated in oxidative phosphorylation and lipid metabolism (cluster 1), Golgi vesicle transport (cluster 2), and protein translation (cluster 3) highlighted.

We next assessed protein pathways affected in SMA iHeps. For an overview of changes in the entire proteome in liver of SMA patients, SMA0–SMA3 and WT iHeps were subjected to quantitative proteome analysis. From the 657 proteins differentially expressed in at least one of the SMA phenotypes compared with WT, we identified 343 proteins that also exhibited trends correlating with disease severity. To understand their roles in the liver, we performed a STITCH and K-means clustering analysis, which identifies interaction networks between proteins and Gene Ontology (GO) enrichment analysis to identify potential biological processes. The resulting 3 clusters of affected protein networks (Figure 3A) indicate processes related to mitochondrial pathways and lipid metabolism, Golgi and endoplasmic reticulum (ER) transport, and protein synthesis were all dysregulated in SMA iHeps (Supplemental Figures 6–12), with overall downregulation of proteins involved in oxidative phosphorylation (OXPHOS), lipid metabolism, Golgi/ER–related transport, and upregulation of proteins involved in protein synthesis (Figure 3, B–D).

**Figure 4:**
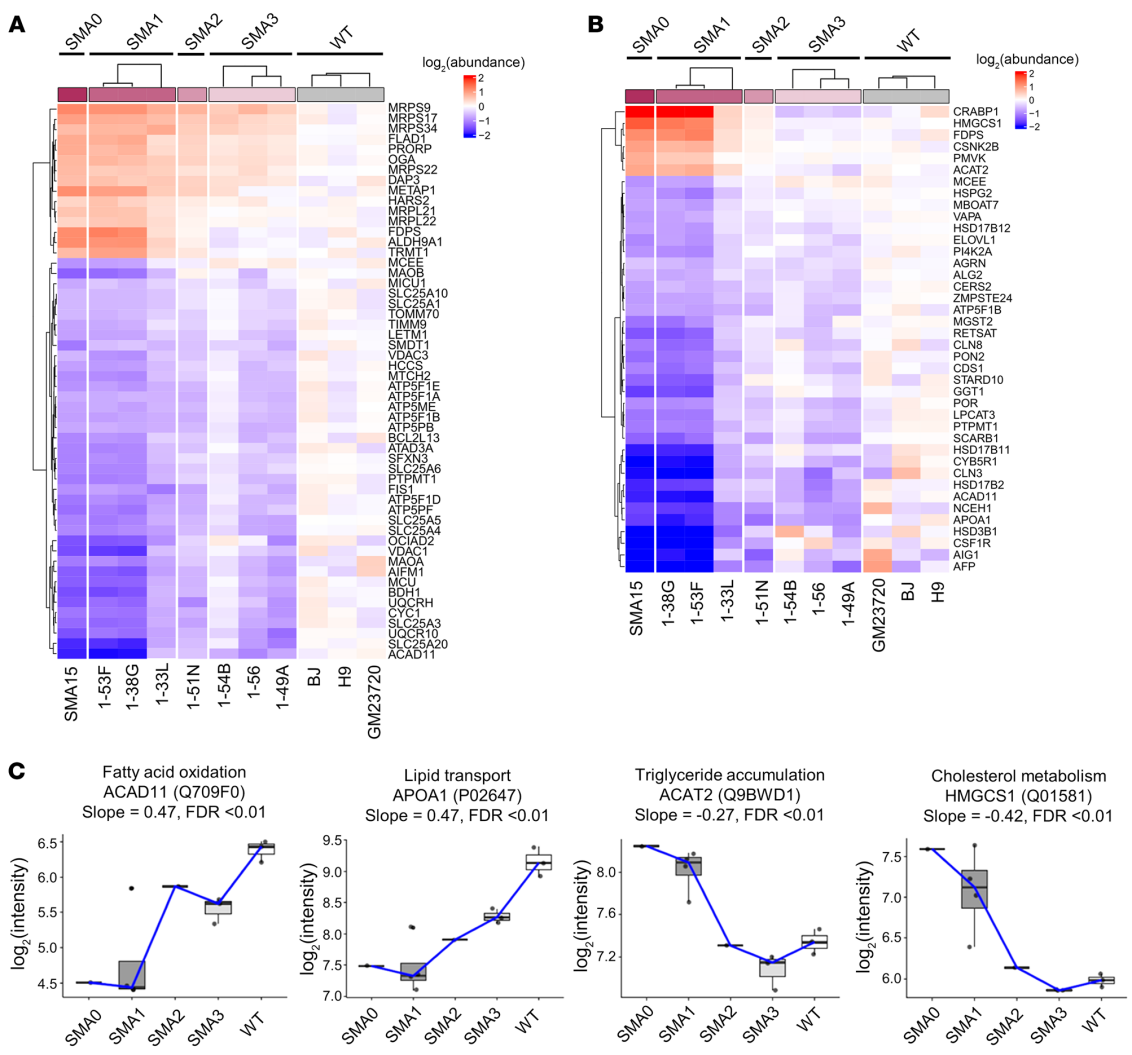
Proteins implicated in mitochondrial processes and lipid metabolism. Protein hits for (**A**) mitochondria and (**B**) lipid metabolism processes based on the UniProt database (see Supplemental Methods) were used to generate the heatmap. (**C**) Differentially expressed proteins between WT-, SMA3-, SMA2-, SMA1-, and SMA0-derived iHeps involved in fatty acid oxidation, lipid transport, triglyceride accumulation, and cholesterol metabolism, respectively. In the box-and-whisker plots, the line in the box represents the median, the bounds on box represent the interquartile range (IQR), and the whiskers represent 1.5 times IQR away from the bounds of the box (Q1 and Q3). Any points outside of the whiskers were considered outliers.

To further understand how mitochondrial dysfunction and lipid homeostasis may influence hepatic steatosis in SMA, we performed an in-depth analysis of dysregulated proteins related to mitochondrial processes and lipid homeostasis using GO methodology (Supplemental Figures 9 and 11). Top GO terms generated included “Mitochondrial ATP synthesis coupled proton transport,” “ATP biosynthetic process,” “Cholesterol metabolism,” and “Fatty acid metabolism” (Supplemental Figure 9C and Supplemental Figure 11B). Fifty-four protein hits matched to the MitoCarta3.0 mitochondrial protein and pathway inventory (https://www.broadinstitute.org/mitocarta/ Accessed March 27, 2023.). Most strikingly, mitochondrial proteins involved in mitochondrial protein translation and ribosomal complex were found to be upregulated, whereas several ATP complex V subunits were downregulated (Figure 4A). Top mitochondrial pathways affected were “Metabolism,” “Small molecule transport,” “Mitochondrial central dogma,” “OXPHOS,” and “Mitochondrial signalling” (Supplemental Figure 10). In addition, specific proteins associated with lipid transport (APOA1) and fatty acid oxidation (ACAD11) were downregulated and positively correlated with increasing SMA severity. The converse was true for proteins associated with cholesterol synthesis (HMGCS1) and triglyceride accumulation (ACAT2), which were upregulated (Figure 4, B and C). These findings suggest that mitochondrial dysfunction and lipid metabolism may contribute to the observed SMA liver phenotype.

### SMA iHeps show metabolic dysfunction.

**Figure 5:**
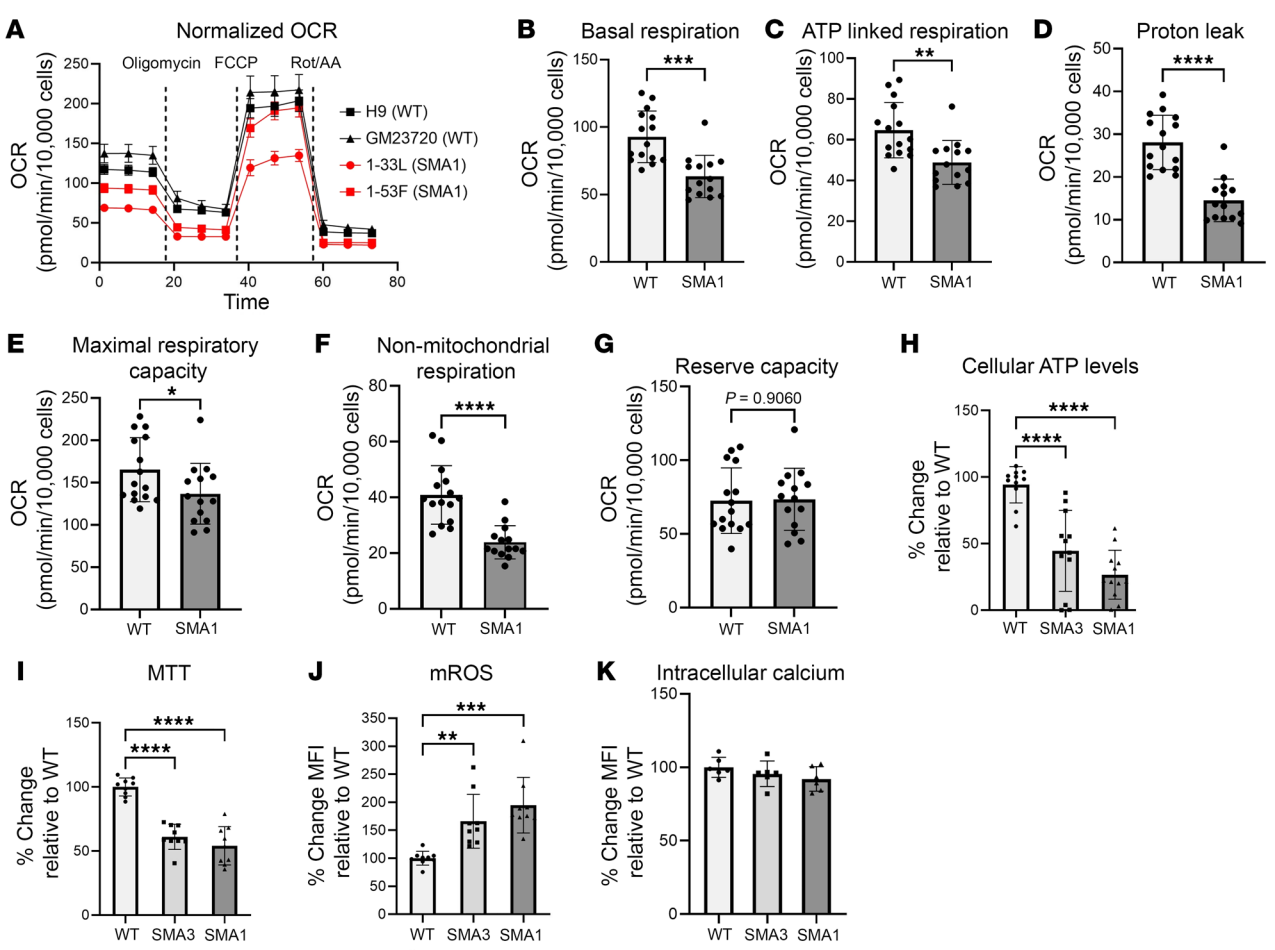
Functional assays showing metabolic dysfunction in day 24 SMA iHeps. (**A**) Measurements of oxygen consumption rate (OCR) between WT and SMA type 1 (SMA1) iHeps (error bars = SEM; H9 *n* = 8, GM23720 *n* = 7, 1-33L *n* = 6, 1-53F *n* = 8). H9 and GM23720 are biological replicates for WT, and 1-33L and 1-53F are biological replicates for SMA1. Analysis of (**B**) basal respiration, (**C**) ATP-linked respiration, (**D**) proton leak, (**E**) maximal respiratory capacity, (**F**) non-mitochondrial respiration, and (**G**) mitochondrial reserve capacity. In **B**–**G**, WT *n* = 15, SMA1 *n* = 14. Cellular assays in day 24 iHeps measuring (**H**) intracellular ATP (WT *n* = 11, SMA1 *n* = 12, SMA3 *n* = 12, each with 3 biological replicates). One outlier from WT was removed using the ROUT test with a maximum FDR of 1%. Data are from 4 independent experiments. (**I**) Cellular metabolic activity by MTT assay. (**J**) Mitochondrial ROS levels by MitoSOX assay. In **I** and **J**, WT *n* = 9, SMA1 *n* = 9, SMA3 *n* = 9, each with 3 biological replicates; data are from 3 independent experiments. (**K**) Intracellular cytosolic calcium levels by Fluo-4 AM assay (WT *n* = 6, SMA1 *n* = 6, SMA3 *n* = 6, each with 3 biological replicates; data are from 2 independent experiments). In **J** and **K**, flow cytometry was performed to obtain MFI, where 10,000 events were recorded and viable cells were then gated. Data are presented as mean ± SD. *P < 0.05, **P < 0.01, ***P < 0.001, ****P < 0.0001 by unpaired, 2-tailed Student’s *t* test (**B**–**G**) or 1-way ANOVA with Tukey’s multiple-comparison test (**H**–**K**).

Since dysregulation of mitochondrial ATP synthesis was implicated as an important disease pathway in the SMA liver phenotype, we tested whether mitochondrial bioenergetics were compromised in SMA1 iHeps using mitochondrial respirometry (35). Mitochondrial oxygen consumption rate (OCR) of WT and SMA1 iHeps was tested using a Seahorse analyzer and showed impairment of mitochondrial bioenergetics in SMA iHeps across multiple indicators (Figure 5, A–G), including ATP-linked respiration (Figure 5C). We then performed cellular metabolic assays on WT and SMA iHeps and showed that SMA iHeps had reduced intracellular ATP (Figure 5H) and succinate dehydrogenase activity using an MTT assay (Figure 5I). Defective mitochondrial respiration and ATP production can lead to increased mitochondrial oxidative stress (36) and compromised mitochondrial membrane potential (MMP) (37). SMA iHeps showed a decrease in MMP (Supplemental Figure 14) and an associated increase in mitochondrial ROS (mROS) production (Figure 5J). Mitochondria have an important role in buffering intracellular calcium, which is dysregulated in SMA astrocytes (38), SMA cardiomyocytes (39), SMA neurons (40), and amyotrophic lateral sclerosis, another motor neuron disease (41). However, we found no difference in intracellular calcium levels in SMA iHeps compared to WT (Figure 5K), suggesting that mitochondrial defects in SMA iHeps might not be mediated through calcium-dependent pathways (42). These findings show that multiple indicators of metabolic dysfunction are present in SMA iHeps.

### SMA iHeps show dysregulation of genes implicated in mitochondrial function, metabolism, and hepatic function, and critical proteins involved in mitochondrial electron transport chain and fatty acid oxidation.

Since SMN regulates gene transcription (43), we evaluated the expression of genes related to the changes in proteome analysis initially generated (Figures 3 and 4). We hypothesized that genes relating to metabolism and hepatic function, previously identified, may also be affected (Supplemental Figure 3A and Supplemental Figure 4A).

**Figure 6:**
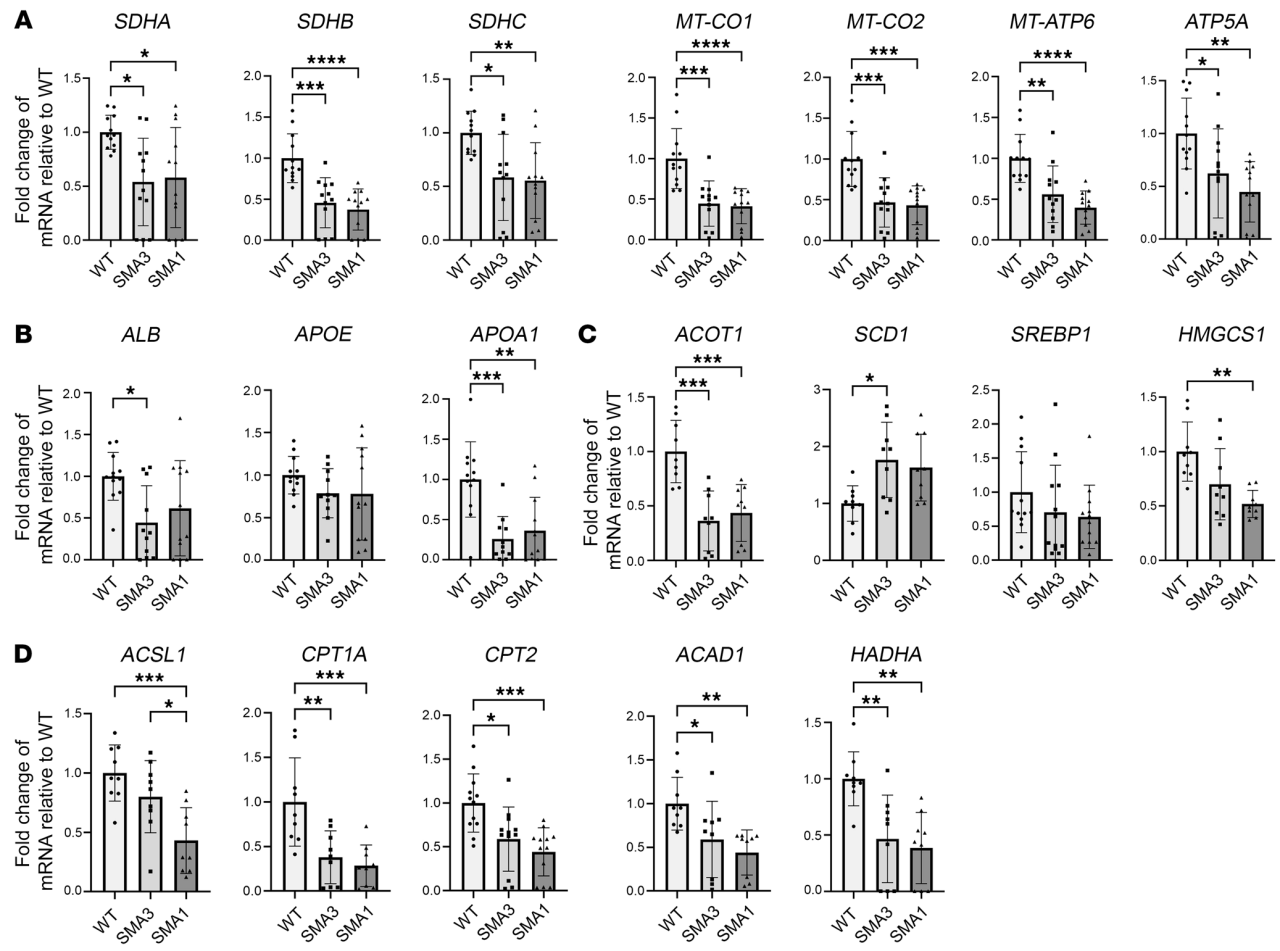
Day 24 SMA iHeps show dysregulation of genes implicated in mitochondrial function and lipid metabolism. (**A**) RT-qPCR of mitochondrial OXPHOS-related genes. (**B**) RT-qPCR of lipid transport genes. For *ALB*, *APOE*, and *APOA1*, one outlier from SMA3 was removed using the ROUT test with a maximum FDR of 1%. (**C**) RT-qPCR of lipid and cholesterol synthesis pathway genes. (**D**) RT-qPCR of β-oxidation pathway genes. Data are from 3 to 4 independent experiments, each with 3 biological replicates. In **A**–**D**, unless specifically indicated that outliers were removed, analysis of data from 3 independent experiments included 9 samples (*n* = 9) each for WT, SMA3, and SMA1 conditions. Similarly for analysis of data from 4 independent experiments, *n* = 12 for each condition. Data were analyzed using 1-way ANOVA with Tukey’s multiple-comparison test and are presented as mean ± SD. *P < 0.05, **P < 0.01, ***P < 0.001, ****P < 0.0001.

Genes coding for proteins involved in mitochondrial OXPHOS — succinate dehydrogenase complex flavoprotein subunits A, B, and C (*SDHA*, *SDHB*, and *SDHC*, respectively; code for proteins in complex II), mitochondrial cytochrome c oxidase subunits I and II (*MT-CO1* and *MT-CO2*, respectively; code for proteins in complex IV), and ATP synthase F0 subunit 6 and ATP synthase F1 subunit α (*MT-ATP6* and *ATP5A*) — all showed reduced transcription in SMA iHeps, but this was not correlated to severity of SMA phenotype (Figure 6A). Lipid transport–related genes *ALB* and *APOA1* showed reduced transcription, whereas *APOE* showed no change in SMA iHeps (Figure 6B). Lipid and cholesterol metabolism genes acyl-CoA thioesterase 1 (*ACOT1*) and 3-hydroxy-3-methylglutaryl-CoA synthase 1 (*HMGCS1*) showed reduced transcription, whereas stearoyl-CoA desaturase (*SCD1*), the rate-limiting enzyme that catalyses biosynthesis of monounsaturated fatty acids that serve as substrates for de novo lipogenesis (44), had increased transcription, while sterol regulatory element binding transcription factor 1 (*SREBP1*), which plays a key role in inducing lipogenesis in the liver, showed no change (Figure 6C). Fatty acid β-oxidation–related (FAO-related) genes carnitine palmitoyltransferase 1A (*CPT1A*), carnitine palmitoyltransferase 2 (*CPT2*), acyl-CoA synthetase long chain family member 1 (*ACSL1*), acyl-CoA dehydrogenase medium chain (*ACAD1*), and hydroxyacyl-CoA dehydrogenase trifunctional multienzyme complex subunit α (*HADHA*) (44) showed reduced transcription (Figure 6D).

**Figure 7:**
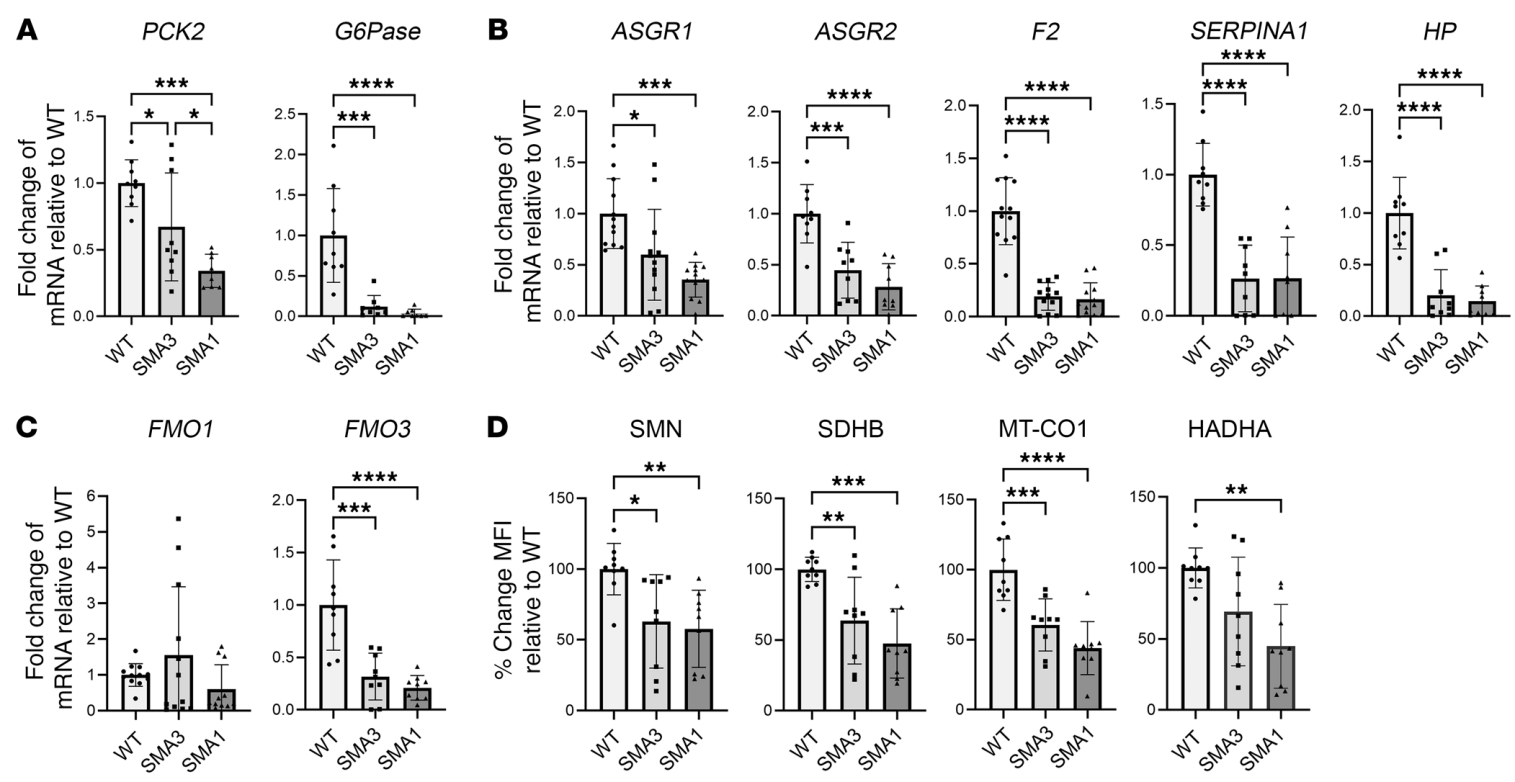
Day 24 SMA iHeps show dysregulation of genes implicated in gluconeogenesis and drug metabolism and critical proteins involved in mitochondrial electron transport chain and fatty acid oxidation. (**A**) RT-qPCR of gluconeogenesis pathway genes. For *PCK2*, one outlier from SMA1 was removed using the ROUT test with a maximum FDR of 1%. For *G6Pase*, one outlier from SMA3 and SMA1 was removed. (**B**) RT-qPCR of iHep function genes. (**C**) RT-qPCR of drug metabolism genes. For *FMO1*, one outlier from SMA1 was removed. In **A**–**C**, for all RT-qPCR, fold change results were derived using the comparative ΔΔCt method. (**D**) Flow cytometric analysis of critical proteins involved in mitochondrial electron transport chain (SDHB and MT-CO1) and fatty acid oxidation (HADHA), with correlation to SMN protein expression in day 24 SMA iHeps. MFI readings were obtained through the recording of 10,000 events followed by gating of the viable iHeps. In **A**–**D**, unless specifically indicated that outliers were removed, analysis of data from 3 independent experiments included 9 samples (*n* = 9) each for WT, SMA3, and SMA1 conditions. Similarly for analysis of data from 4 independent experiments, *n* = 12 for each condition. Data are presented as mean ± SD and were analyzed using 1-way ANOVA with Tukey’s multiple-comparison test. *P < 0.05; **P < 0.01; ***P < 0.001; ****P < 0.0001.

SMA patients are prone to becoming hypoglycemic after fasting (45, 46), a finding thought to be associated with altered gluconeogenesis. In line with this, gluconeogenesis-related genes phosphoenolpyruvate carboxykinase 2 (*PCK2*) and glucose-6-phosphatase catalytic subunit 1 (*G6Pase*) showed reduced transcription (Figure 7A).

Genes associated with hepatic function, including glycoprotein homeostasis (*ASGR1*, *ASGR2*) (47), clotting (*F2*), fibrosis and complement activation (*SERPINA1*) (48), and hemolysis (*HP*) (49) also showed reduced transcription (Figure 7B).

Finally, *FMO1* and *FMO3*, which encode flavin-containing dimethylaniline monooxygenase-1 and -3, respectively, were analyzed, as these enzymes are the primary metabolizers of risdiplam, an approved therapeutic SMN splice modifier. *FMO3* transcription was reduced, but *FMO1* was unchanged (Figure 7C).

To evaluate concordance of hepatic protein expression with gene transcription and show changes in protein levels at an individual iHep cell level, expression of key proteins SDHB, MT-CO1, HADHA, and SMN in iHeps was analyzed using flow cytometry (Figure 7D). SDHB and MT-CO1 were specifically selected as important members of the electron transport chain and HADHA due to its critical role in FAO.

Concordant with reduced gene transcription, SDHB, MT-CO1, and HADHA all showed reduced expression in SMA iHeps, which associated with reduced SMN protein expression. Concordance of gene-protein expression was also noted for ATP5A and APOA1, key proteins involved in mitochondrial OXPHOS and lipid metabolism, which were hits from proteome analysis (Figure 4), were downregulated in their gene transcription (Figure 6, A and B).

These findings demonstrate that defects in SMA iHeps encompass perturbations in mRNA expression for genes involved in mitochondrial OXPHOS, lipid metabolism, gluconeogenesis, drug metabolism, and hepatic function, and dysregulated expression of key proteins at a single-cell level. These are similar in SMA1 and SMA3.

### SMA phenotype can be rescued by SMN repletion in SMA1 iHeps.

We hypothesized that primary defects in SMA liver were caused by hepatocyte SMN protein deficiency and performed CRISPR/Cas9-mediated genome editing of endogenous *SMN2* in an SMA1 iHep to study SMN-dependent effects without the confounding effect of the different genetic backgrounds of SMA patients. We converted c.840 T>C in exon 7 to engineer an *SMN1*-like gene that permanently restored functional SMN protein expression levels in the SMA1 iHeps (Supplemental Figure 15A) to WT levels when both *SMN2* genes were edited to create isogenic WT clones, and to 50% of WT levels when only 1 *SMN2* gene was edited to create isogenic carrier clones (Supplemental Figure 15B).

**Figure 8:**
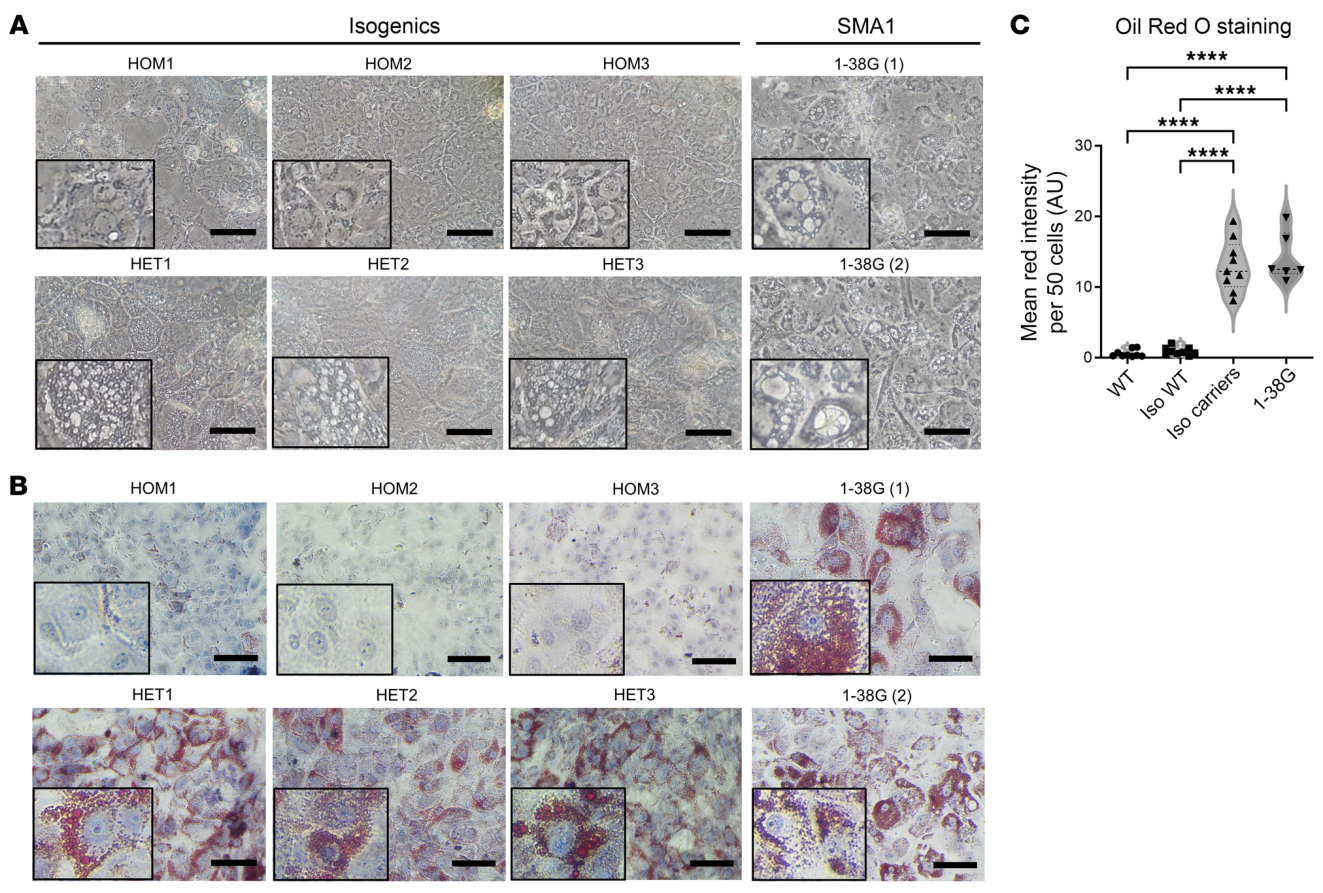
Rescue of lipid accumulation with SMN repletion in day 24 SMA type 1 iHeps. (**A**) Bright-field microscopy images of day 24 isogenic WT, isogenic carriers, and 1-38G (SMA1) iHeps. Scale bars: 50 μm. Boxed portions represent zoomed-in segments of the original image to showcase vacuole enlargement more clearly. (**B**) Oil Red O staining of day 24 isogenic WT, isogenic carriers, and 1-38G iHeps, showing decrease in lipid accumulation after repletion of SMN. Boxed portions represent zoomed-in segments of the original image to showcase oil red staining of triglycerides and lipids with more clarity. Scale bars: 50 μm and 25 μm (zoomed-in images). (**C**) The mean red intensity of 50 cells is presented. WT, isogenic (Iso) WT, and Iso carriers have 3 biological replicates each. Data are representative of 3 independent experiments (WT *n* = 9, Iso. WT *n* = 9, Iso carriers *n* = 9, 1-38G *n* = 6). Data were analyzed using 1-way ANOVA with Tukey’s multiple-comparison test and are presented as mean ± SD. ****P < 0.0001.

**Figure 9:**
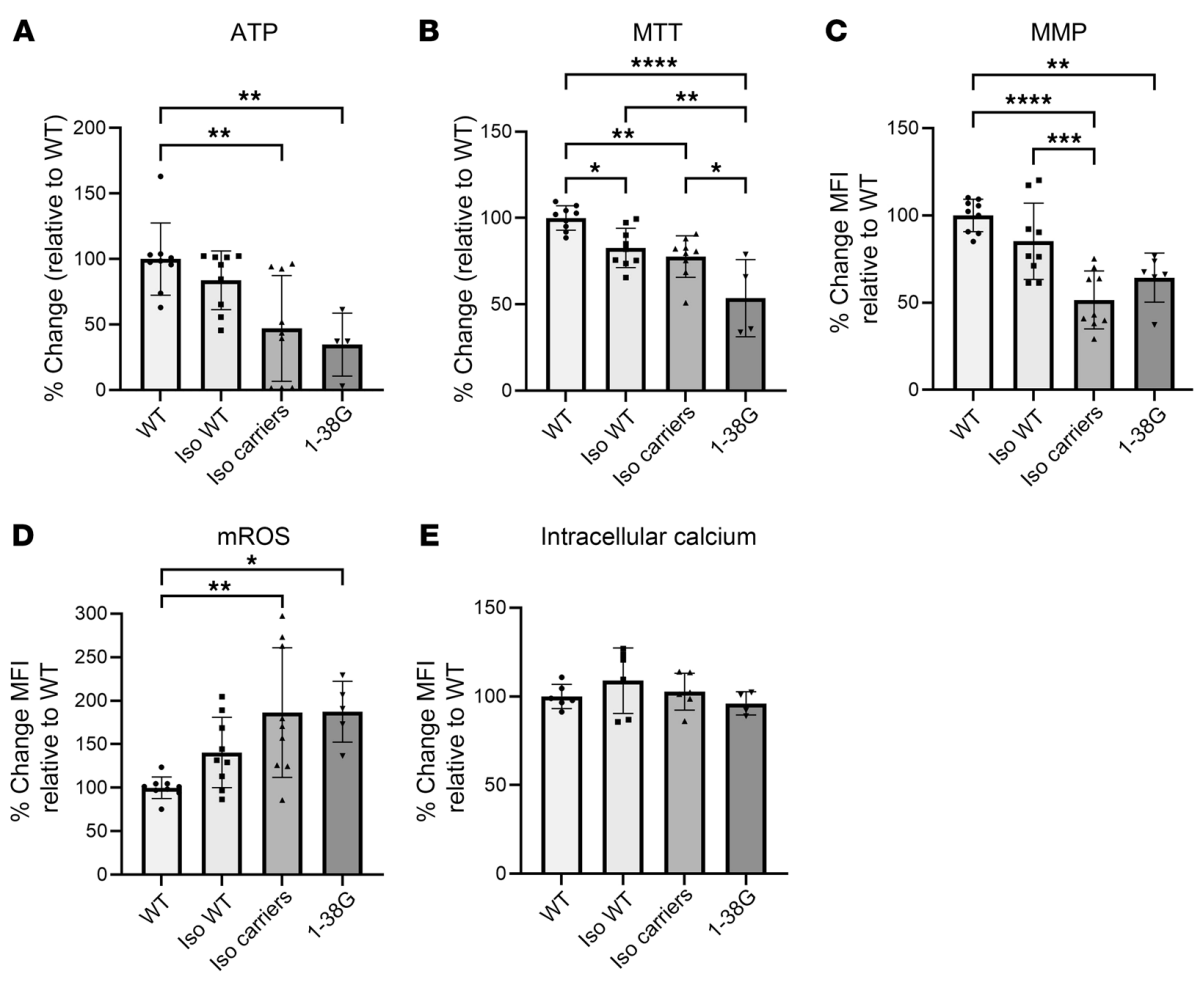
Rescue of metabolic dysfunction with SMN repletion in day 24 SMA type 1 iHeps. (**A**–**D**) Cellular assays in day 24 iHeps. (**A**) intracellular ATP (WT *n* = 9, isogenic [Iso] WT *n* = 9, Iso carriers *n* = 9, 1-38G *n* = 4). (**B**) Cellular metabolic activity by MTT assay (WT *n* = 9, Iso. WT *n* = 9, Iso carriers *n* = 9, 1-38G *n* = 4). (**C**) Mitochondrial membrane potential (MMP) by TMRM assay (WT *n* = 9, Iso WT *n* = 9, Iso carriers *n* = 9, 1-38G *n* = 6). (**D**) Mitochondrial ROS levels by MitoSOX assay (WT *n* = 9, Iso. WT *n* = 9, Iso carriers *n* = 9, 1-38G *n* = 5). Data in **A**–**D** are from 3 independent experiments. WT, Iso WT, and Iso arriers have 3 biological replicates each. (**E**) Intracellular cytosolic calcium levels by Fluo-4 AM assay. Data are from 2 independent experiments (WT *n* = 6, Iso. WT *n* = 6, Iso carriers *n* = 6, 1-38G *n* = 4). In **C**–**E**, MFIs for TMRM, MitoSOX, and Fluo-4 AM assays were obtained using flow cytometry, where 10,000 events were recorded, and the viable cells were then gated. In **A**–**E**, all results were quantified as a percentage relative to the mean of the WTs. Data were analyzed using 1-way ANOVA with Tukey’s multiple-comparison test and are presented as mean ± SD. *P < 0.05; **P < 0.01; ***P < 0.001; ****P < 0.0001.

**Figure 10:**
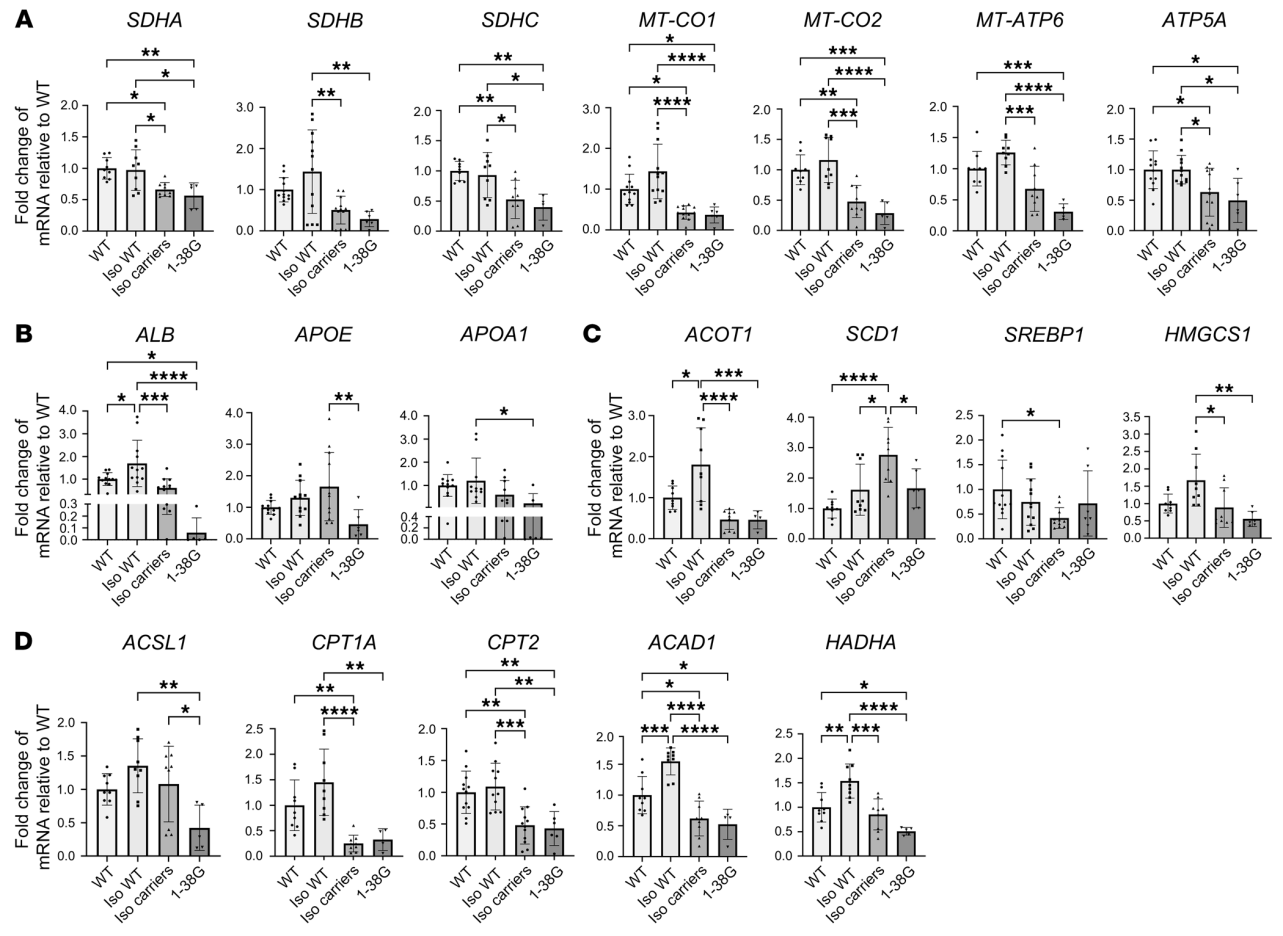
Rescue of genes implicated in mitochondrial function and lipid metabolism with SMN repletion in day 24 SMA type 1 iHeps. (**A**) RT-qPCR of mitochondrial OXPHOS-related genes. (**B**) RT-qPCR of lipid transport genes. For *ALB*, one outlier from 1-38G was removed using the ROUT test with a maximum FDR of 1%. For *APOA1*, 2 outliers from isogenic (Iso) carriers and 1 outlier from Iso WT was removed. (**C**) RT-qPCR of lipid and cholesterol synthesis pathway genes. For *SREBP1*, one outlier from Iso carriers was removed. (**D**) RT-qPCR of fatty acid β-oxidation pathway genes. For *CPT2*, one outlier from Iso. WT was removed. In **A**–**D**, data are from 3 to 4 independent experiments. Unless specifically indicated that outliers were removed, analysis of data from 3 independent experiments included 9 samples (*n* = 9) each for WT, Iso WT, and Iso carrier conditions. Similarly for analysis of data from 4 independent experiments, *n* = 12 for each condition. For 1-38G, *n* ≥ 4 for all experiments. Data were analyzed using 1-way ANOVA with Tukey’s multiple-comparison test and are presented as mean ± SD. *P < 0.05; **P < 0.01; ***P < 0.001; ****P < 0.0001.

**Figure 11:**
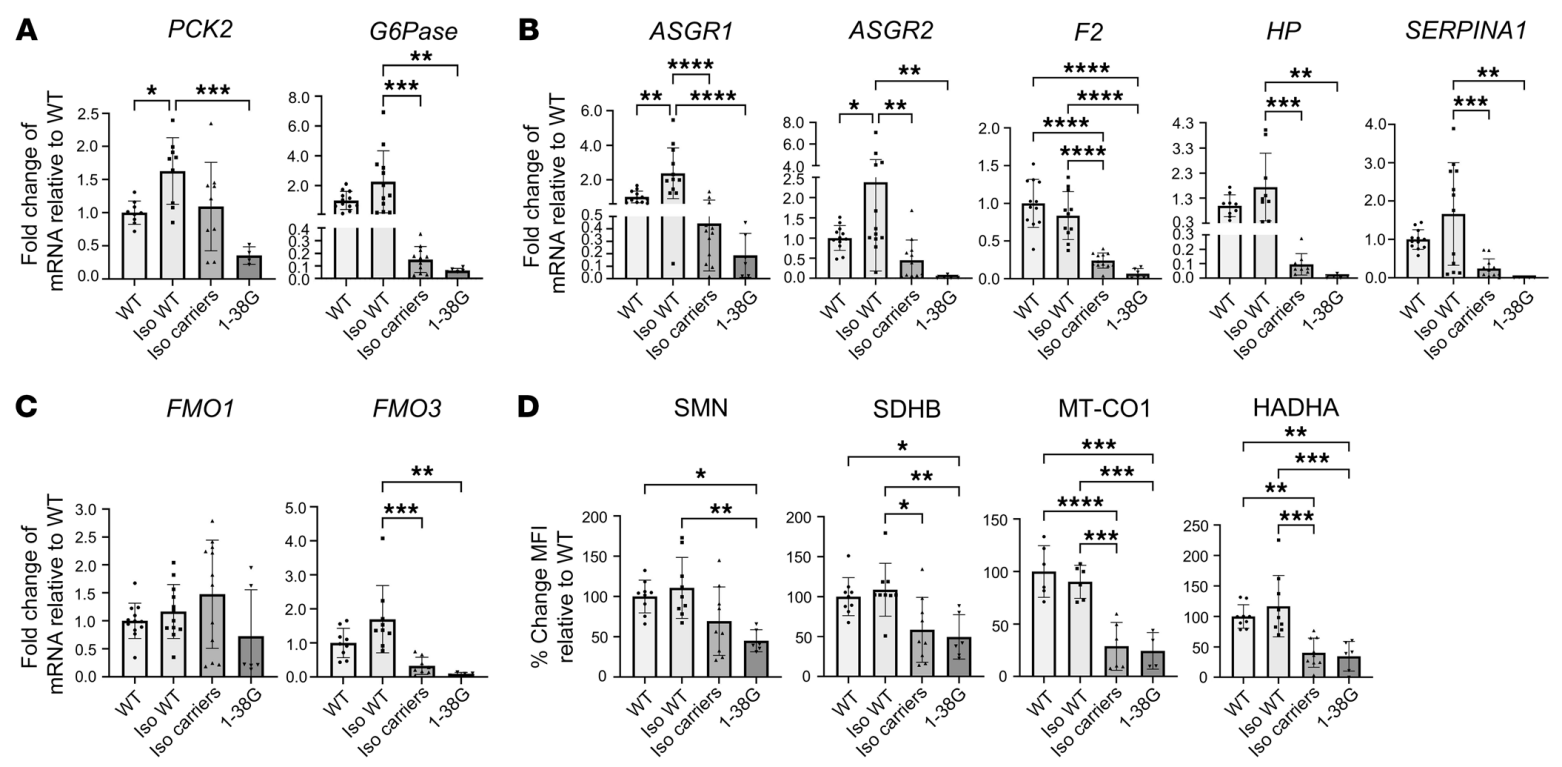
Rescue of genes implicated in gluconeogenesis and drug metabolism and critical proteins involved in mitochondrial electron transport chain and fatty acid oxidation with SMN repletion in day 24 SMA type 1 iHeps. (**A**) RT-qPCR of gluconeogenesis pathway genes. For *G6Pase*, one outlier from isogenic (Iso) carrier and 1 outlier from 1-38G was removed using the ROUT test with a maximum FDR of 1%. (**B**) RT-qPCR of iHep function genes. For *ASGR2*, one outlier from 1-38G was removed. For *F2*, one outlier from Iso WT was removed. For *SERPINA1*, one outlier from 1-38G was removed. (**C**) RT-qPCR of drug metabolism genes. For *FMO3*, one outlier from Iso carriers was removed. (**A**–**C**) For all RT-qPCR, fold change results were derived using the comparative ΔΔCt method. Data are from 3 to 4 independent experiments. Unless specifically indicated that outliers were removed, analysis of data from 3 independent experiments included 9 samples (*n* = 9) each for WT, Iso WT, and Iso carrier conditions. Similarly for analysis of data from 4 independent experiments, *n* = 12 for each condition. For 1-38G, *n* ≥ 4 for all experiments. (**D**) Flow cytometric analysis of critical proteins involved in mitochondrial electron transport chain (SDHB and MT-CO1) and fatty acid oxidation (HADHA) with correlation to SMN protein expression. MFI was obtained by recording 10,000 events and viable iHeps were then gated for. Data are from 2 to 3 independent experiments. Analysis of data from 2 independent experiments included 9 samples (*n* = 6) each for WT, Iso WT, and Iso carrier conditions. Similarly for analysis of data from 3 independent experiments, *n* = 9 for each condition. For 1-38G, *n* ≥ 4 for all experiments. In **A**–**D**, data were analyzed using 1-way ANOVA with Tukey’s multiple-comparison test and are presented as mean ± SD. *P < 0.05; **P < 0.01; ***P < 0.001; ****P < 0.0001.

Intracellular vacuole phenotype (Figure 8A) and ORO staining (Figure 8, B and C) seen in SMA1 iHeps and isogenic carriers was completely rescued to WT levels by repletion of SMN protein levels and creation of isogenic WT iHeps (refer to Figure 1C for WT bright-field and Figure 2A for WT ORO staining images). Mean ORO staining intensity per 50 cells in each line is presented in Supplemental Figure 5B. Normalization of mean ORO staining intensity to cell number is presented in Supplemental Figure 5D. Intracellular ATP, MTT, MMP, and mROS levels were also rescued to near WT levels (Figure 9, A–E). Transcription of genes related to mitochondrial OXPHOS (Figure 10A), lipid transport (Figure 10B), lipid and cholesterol metabolism (Figure 10C), FAO (Figure 10D), gluconeogenesis (Figure 11A), hepatic function (Figure 11B), drug metabolism (Figure 11C), and expression of key proteins in mitochondrial electron chain transport and FAO, were also rescued with repletion of SMN protein to WT levels on an isogenic background (Figure 11D).

Partial rescue of SMN protein expression in isogenic carriers did not rescue steatosis in iHeps nor did it rescue most genes with dysregulated transcription in SMA1, suggesting that presence of only 1 *SMN1*-like gene cannot fully compensate for the SMA1 phenotype. This may support a “threshold” model in which cells and tissues have differential requirements for SMN and corresponding susceptibilities to SMN depletion (14).

These specific SMN repletion and rescue findings confirm that the described cellular defects are caused by a hepatocyte-aintrinsic deficiency in SMN.

## Discussion

In this study, we provide evidence across the spectrum of SMA clinical phenotypes for susceptibility to hepatic steatosis in an SMA patient cohort and in vitro human models of SMA. Consistency of liver phenotype in patients and a preclinical hiPSC model provides evidence that SMN depletion predisposes SMA patients to fatty liver and liver dysfunction. Furthermore, our findings show that this is a primary liver defect resulting from hepatocyte-intrinsic SMN protein deficiency. Additionally, although the size of our patient cohort is small, the fact that steatosis was reported in a majority of patients undergoing treatment suggests that hepatopathology may not be adequately targeted by current therapies given at the times when these patients received treatment. Of the current therapies, onasemnogene abeparvovec and risdiplam may be expected to increase SMN levels in the liver, as they act systematically. More studies are required to understand the therapeutic window for SMN-repleting therapies in extraneuronal tissues in addition to motor neurons and how these relate to individualized therapies.

The strategy of genome editing *SMN2* to restore native SMN protein expression has been most recently used to rescue SMA phenotypes in mice (50). Here, we used CRISPR/Cas9 genome editing to rescue SMN protein expression from endogenous *SMN2* on an isogenic background, and showed that SMN protein repletion is sufficient to rescue this hepatocyte-intrinsic SMA phenotype. To our knowledge, this is the first time such findings have been reported in SMA.

**Figure 12:**
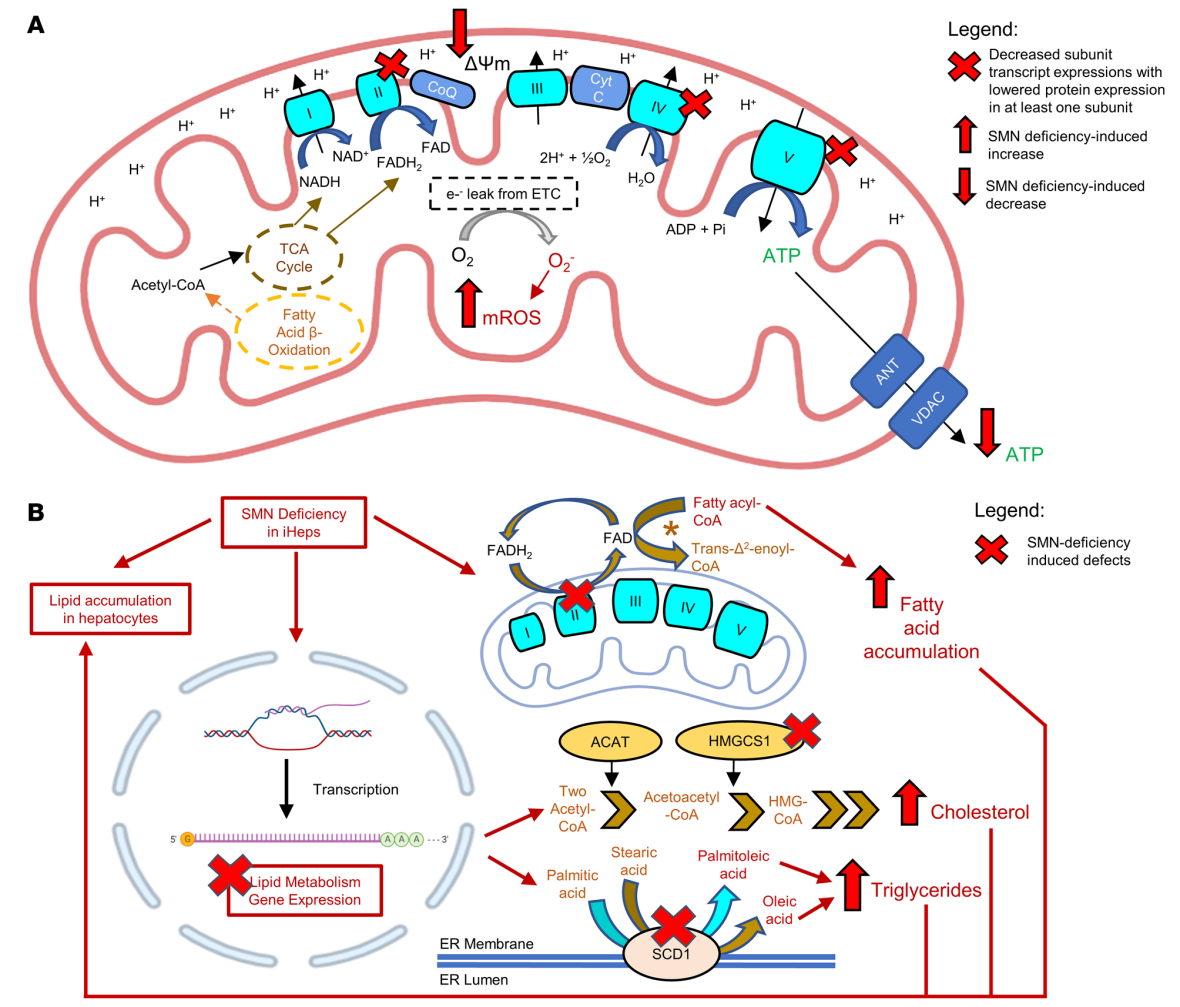
Schematic diagram of proposed mitochondrial dysfunction and lipid metabolism defects in SMA iHeps. (**A**) Mitochondrial dysfunction in SMA iHeps. Dysfunction/dysregulation in mitochondrial complexes II, IV, and V, and voltage-dependent anion-selective channel protein (VDAC), as a result of decreased mRNA and/or protein subunit expression. (**B**) Lipid metabolism defects in SMA iHeps. Lipid metabolism gene expression dysregulation includes enzymes involved in fatty acid β-oxidation, lipid synthesis, and cholesterol synthesis pathways. Increased fatty acid accumulation due to cross-talk defects between mitochondria and fatty acid β-oxidation, increased cholesterol synthesis due to increased protein expression of HMGCS1, and increased triglyceride synthesis due to increased expression of *SCD1* mRNA may account for overall lipid accumulation in SMA iHeps, as observed from Oil Red O staining assay. Portions of the figure were created with BioRender.com.

Overall, we provided insights into the mechanisms that are responsible for fatty liver and liver dysfunction in SMA patients, and generated a hypothetical model to link these. Here, downregulation of genes and proteins relating to mitochondrial complexes II, IV, and V and enzymes related to FAO, lipid synthesis, and cholesterol synthesis drive abnormal lipid accumulation in SMA liver (Figure 12). Although mRNA-protein expression discrepancy was noted for HMGCS1, this may be attributed to additional levels of regulation existing between transcript and protein, as Golgi/ER transport and protein synthesis pathways were affected (Figure 3, C and D). We postulated that the extent of lipid accumulation identified in SMA iHeps would have functional consequences. Indeed, there were changes in gene transcription regulating additional hepatic functions relating to serum glycoprotein homeostasis, coagulation, complement, hemolysis, gluconeogenesis, and drug metabolism. These findings were similar across various SMA genotypes. Previous studies in motor neurons and skeletal muscle have shown mitochondrial dysfunction (51) and altered ER to Golgi transport (52, 53) in SMA. Concordant with protein hits identified in our proteome analysis, proteins involved in the mitochondrial OXPHOS pathway (Figure 3B) — ATP synthase peripheral stalk-membrane subunit b (ATP5F1), ATP synthase F1 subunit α (ATP5A), ATPase H^+^-transporting V1 subunit A (ATP6V1A), and cytochrome c1 (CYC1) — were found to be dysregulated in SMN^–/–^ mouse hippocampal neuronal cells (54) and motor neurons in a mouse model of SMA (55). These findings of similar changes validate our model’s ability to capture elements of SMA patients’ conditions and support a multisystemic view of changes in cellular processes in SMA.

Although most SMA patients are normally reported to be clinically asymptomatic in liver function, this may reflect the fact that liver function is not routinely checked in SMA patients, and if symptoms relating to liver dysfunction are reported by patients, they are not usually connected as related to the SMA phenotype. Furthermore, extraneuronal organs, such as liver, are mitotic and have a tremendous capacity to regenerate and cope with disease, unlike the central nervous system, which frequently masks underlying pathology. While ultrasonic evidence of liver steatosis was present in a number of our SMA patients, this phenotype was present on histology in all SMA iPSC–derived iHeps. This suggests that histological examination may be more sensitive than ultrasonography in detecting liver abnormalities in patients. SMN deficiency may predispose hepatocytes to functional impairment at a cellular level and increase susceptibility to multiple injuries induced by age and environmental stresses. This would be particularly relevant for the newly aging demographics of treated SMA patients (10, 13), and has implications for adverse drug reactions involving the liver, such as with gene therapy. Furthermore, these data add to accumulating evidence demonstrating cell-intrinsic defects in response to SMN depletion, including muscle (56), pancreas (57), Schwann cells (58, 59), and endothelial cells (60).

Drugs metabolized by the liver, such as risdiplam, may not reach optimal pharmacokinetics or may cause more significant hepatotoxicity or other side-effects if their primary metabolizing enzymes are affected. We have shown that *FMO3* transcription is SMN dependent and reduced in SMA iHeps. FMO3 is the main metabolizer of risdiplam and makes up 75% of its metabolism (61). Risdiplam is a lifelong treatment and if the drug induces its own metabolism by increasing SMN protein expression, risdiplam dosage may have to be increased with longer duration of treatment to maintain clinical efficacy as children with SMA age and grow. If there is already subclinical liver damage in SMA, drugs known to cause mild hepatotoxicity may cause precipitous liver failure, with severe clinical consequences including mortality. Gene therapy is known to cause mild hepatotoxicity that resolves with steroid treatment (10). However, subacute liver failure has been reported in 2 children with SMA1 following gene therapy, with elevations in AST, ALT, GGT, and INR consistent with hepatocellular damage and defects in hepatic synthetic function, despite receiving steroids before and after infusion as per current standard of care (62). More concerning has been the death of 2 children from acute liver failure in 2022 after receiving gene therapy. This has led to guidelines (63) for comprehensive pretreatment screening for liver disease in candidate SMA patients considering gene therapy (62). Up to now, there has typically been only perfunctory examination of liver function and imaging in SMA clinics. Future studies could look into liver phenotypes of patients with severe adverse reactions to gene therapy, or patients who are nonresponders to risdiplam treatment, to allow early identification of vulnerable patients so appropriate clinical management plans can be made.

Interestingly, while extraneuronal pathology of SMA may be asymptomatic and not well understood, there is evidence to suggest that it may have an important role in survival of motor neurons. Rescue of SMN solely in peripheral tissues, including liver, in mouse models of SMA, markedly prolongs overall survival, improves motor neuron survival, and increases preservation of neuromuscular junctions (64–66). Conversely, selective depletion of SMN in motor neurons alone results in a milder SMA phenotype as compared with systemic depletion (67), while selective restoration of SMN in neural tissue leads to only partial rescue of the SMA phenotype (68). It is possible that effects on SMA neuromuscular function could be mediated through peripheral organ secretion of neurotrophic factors such as IGF-1, which, together with its binding protein IGFALS has reduced expression in patients and mouse models of SMA (64, 69). Further studies to investigate whether non–motor neuron, cell-autonomous SMN rescue in liver and other peripheral organs has a role in motor neuron function and overall survival in SMA patients would increase our understanding of the pathology and natural history of SMA, and clinical implications of extraneuronal and treatment-modified phenotypes.

A limitation of our observational clinical study was the retrospective design and small patient numbers. However, we have provided proof of concept that ultrasonic evidence of fatty liver can be determined in SMA patients, which will enable the design of future prospective clinical studies using ultrasound to investigate prevalence of fatty liver in SMA. Liver ultrasonography is a noninvasive method of liver screening compared with liver biopsy. As a potential clinical biomarker for liver dysfunction in SMA, liver ultrasound is less painful and less expensive compared with liver biopsy, possibly more sensitive than clinically available serum markers of liver function, and widely available. Future studies may include combining imaging with expanded blood testing to discover new biomarkers associated with SMA disease processes. This will be of interest if combined with longitudinal patient follow-up for identifying patients with increased risk of adverse drug effects. Another limitation is that mitochondrial size and morphology could have confounded our analysis of MMP and mROS levels (70). However, we have controlled for this using confocal microscopy (Supplemental Figure 14, A–E). Together with defective mitochondrial bioenergetics observed in SMA iHeps, increased mitochondrial oxidative stress level and reduced MMP suggest impaired function of mitochondria in SMA hepatocytes.

Further studies should attempt to dissect underlying molecular mechanisms and correlate these with clinical phenotypes, delineate the therapeutic window of opportunity in patients, and identify new therapeutic targets. It is the opinion of a number of researchers and clinicians that SMA therapies that increase SMN protein expression must target extraneuronal organs for optimal management of SMA (13–15, 19, 71, 72). These treatments may synergize with SMN-independent therapies by acting in combination on several molecular pathways. Myostatin inhibitors are being studied in a number of clinical trials such as TOPAZ (73), SAPPHIRE (74), RESILIENT (75), and MANATEE (76) to see if they can rescue residual muscle defects in SMA patients and maximize clinical benefit with concurrent SMN-directed treatment. Our study suggests that drugs that treat mitochondrial dysfunction or increase FAO, such as coenzyme Q10, riboflavin, antioxidants like vitamin C and α-lipoic acid, and carnitine, may also synergize with SMN-repleting therapies (77).

Our work highlights the importance of understanding extraneuronal phenotypes of SMA in human models, and details how hiPSCs and genome editing technology can be used to determine whether these phenotypes are cell intrinsic and SMN dependent. It also provides evidence that SMA should be considered and treated as a multicellular and multiorgan disease. Early screening and preventive treatment of fatty liver and other extraneuronal phenotypes is imperative to prevent future comorbidities, and international clinical consensus is vital in establishing systematic clinical surveillance programs and therapeutic strategies that incorporate extraneuronal phenotypes of SMA.

## Methods

### Sex as a biological variable.

Our study examined male and female patients, and similar findings are reported for both sexes.

### Patient data.

In this small single-center retrospective cohort study conducted at Boston Children’s Hospital/Harvard Medical School, a specialty international referral center for SMA, all pediatric and adult SMA patients without any past medical history of liver disease, who were seen physically in clinic from 2020 to 2022, and who had received hepatic sonography or fibroscan, were included (*n* = 8). Liver enzymes and serum markers of liver synthetic function were reviewed.

Sonographic or fibroscan steatosis grade was determined by an ultrasonographer or pediatric gastroenterologist.

Liver steatosis was graded as follows (78): grade I (mild), slightly increased liver brightness relative to that of the kidney with normal visualization of the diaphragm and intrahepatic vessel borders; grade II (moderate), increased liver brightness relative to that of the kidney with slightly impaired visualization of the intrahepatic vessels and diaphragm; and grade III (severe), markedly increased liver brightness relative to that of the kidney with poor or no visualization of the intrahepatic vessel borders, diaphragm, and posterior portion of the right lobe of the liver.

### Stem cell culture and maintenance.

WT/non–SMA patient–derived stem cells (BJ, H9, and GM23720) from Corning were used as a control group for comparison with patient-derived SMA cell lines obtained from Lee Rubin at Harvard University (79). Details of SMA patient–derived hiPSCs used for the stem cell model are reported in Table 2. CRISPR editing of the 1-38G clone was performed to engineer a c.840 T>C transition in exon 7 of *SMN2* to generate the *SMN1*-like gene and derive 3 isogenic WT and 3 isogenic carrier lines (Table 3). Matrigel (354277, Corning) was used to coat plates (37°C for 30 minutes) prior to seeding of hiPSCs. The matrigel was diluted in ice-cold Advanced DMEM/F-12 (12634010, Thermo Fisher Scientific) as per manufacturer’s instructions. iPS Brew XF (130-104-368, Miltenyi Biotec) was used as the stem cell culture medium for hiPSC maintenance. hiPSCs were thawed and diluted with Advanced DMEM/F-12. After centrifugation, the supernatant was discarded and the pellet was resuspended in fresh culture medium and seeded onto the precoated wells. Culture medium was changed on a daily basis and hiPSCs were split using ReLeSR (05872, STEMCELL Technologies) every 5–6 days when hiPSCs reach 70%–80% confluence.

### CRISPR/Cas9 genome editing.

To derive isogenic control (repletion of 2 *SMN1*-like gene copies) and isogenic carrier lines (repletion of 1 *SMN1*-like gene copy), we performed CRISPR/Cas9 genome editing in the 1-38G patient-derived hiPSC line (SMA1). The methodology for conducting the CRISPR/Cas9 genome editing involved electroporation of sgRNA into the 1-38G hiPSCs, checking of editing efficiency, isolation of clonal colonies, genotyping, and off-target analysis of the clones. Finally, the acquired isogenic lines from the parental 1-38G line were frozen in cryovials at –80°C overnight and subsequently transferred to liquid nitrogen for long-term storage. The detailed methodology for how the CRISPR/Cas9 genome editing was performed can be found in the Supplemental Methods. The primers used for amplification and sequencing are listed in Supplemental Table 1.

### iHep differentiation.

After splitting hiPSCs into wells in preparation for iHep differentiation, they were maintained in stem cell culture medium for 2 days to attain approximately 60% confluence. On day 0, the cells were cultured in RPMI/B27 basal differentiation medium (12-702F, Lonza), supplemented with 100 ng/mL Human Activin A (130-115-013, Miltenyi Biotec), 3 μM CHIR99021 (130-106-539, Miltenyi Biotec), and 10 μM LY 294002 (L-7962, LC Laboratories) for 48 hours. On day 3, the cells were supplemented with 50 ng/mL Human Activin A in the basal differentiation medium. On days 6 and 8, the cells were supplemented with 20 ng/mL Human BMP-4 (130-111-168, Miltenyi Biotec) and 10 ng/mL Human FGF-10 (130-127-858, Miltenyi Biotec) in the basal differentiation medium. From day 10 onwards, the RPMI/B27 basal differentiation medium was replaced with hepatocyte culture media (CC-3198, Lonza). On days 10, 13, 15, 17, 20, and 22, the cells were supplemented with 30 ng/mL Human Oncostatin M IS (130-114-942, Miltenyi Biotec) and 50 ng/mL Human HGF (130-103-437, Miltenyi Biotec). On day 24, the iHeps were harvested for further processing. A schematic representation of the differentiation protocol is presented in Supplemental Figure 2.

### Urea assay.

Conditioned hepatocyte culture media from the iHeps were collected and quantified for urea concentration using the QuantiChrom Urea Assay kit (BioAssay Systems). The optical density from each sample at 430 nm was measured and recorded using the Synergy H1 Microplate Reader (BioTek).

### ORO assay.

The ORO assay kit was obtained from Sigma-Aldrich. On day 24, culture media were removed and cells were washed with PBS. Next, cells were fixed with 3.7% paraformaldehyde (Sigma-Aldrich) at 37°C for 30 minutes. Cells were washed with ultrapure water and incubated with 60% isopropanol at 37°C for 5 minutes. Isopropanol was removed and cells were incubated with ORO working solution at 37°C for 15 minutes. Next, ORO was discarded, and cells were washed with ultrapure water until no excess stain remained. Next, cells were incubated with a sufficient volume of hematoxylin at 37°C for 1 minute. After incubation, hematoxylin was discarded and cells were washed with ultrapure water before visualizing under a bright-field microscope (Olympus). ORO images were processed using ImageJ software (NIH).

### Measurement of OCR using Seahorse XFe96 analyzer.

The OCR of the day 28 iHeps was measured using a Seahorse Biosciences XFe96 Extracellular Flux Analyzer (Agilent Technologies). Day 24 iHeps were plated on collagen-coated Seahorse 96-well plates at a cell density of 2.5 × 10^5^ cells per well. The cells were allowed to recover in the 96-well plates for 4 days. Prior to the measurement of OCR, the iHeps were incubated in Seahorse XF DMEM basal media (Agilent Technologies) supplemented with 25 mM glucose (Sigma-Aldrich) and 1 mM sodium pyruvate (Gibco). The respiration profile of the iHeps was subsequently measured by the sequential injection of the following compounds: 2 μM oligomycin, 1 μM FCCP, and 1 μM rotenone/antimycin A. Each compound treatment lasted 18 minutes, with OCR measurements taken every 6 minutes. Upon completion of the Seahorse assay, the iHep cultures were immediately fixed with 4% paraformaldehyde. The iHep cultures were stained with Hoescht 33342 (Thermo Fisher Scientific) followed by high-content imaging (Perkin Elmer). The final OCR values were subsequently normalized by cell count obtained to ensure consistency across OCR measurements.

### MTT assay.

iHeps were trypsinized with Trypsin-EDTA (Biowest) and trypan blue assay was performed to seed 10,000 cells per well in 96-well plates. MTT assay was performed after 24 hours. MTT (3-[4,5-dimethylthiazol-2-yl]-2,5-diphenyl-tetrazolium bromide) (M2003, Sigma-Aldrich) was prepared with PBS. Spent media from 96-well plates were removed and 90 μL of fresh hepatocyte culture media was added per well. Ten microliters of 12 mM MTT was added and iHeps were incubated at 37°C for 1 hour in the dark. After 1 hour, hepatocyte culture media and MTT were removed and 100 μL of dimethyl sulfoxide (DMSO) was added. iHeps were incubated at room temperature for 1 hour in the dark. Absorbance of each well at 595 nm was measured and recorded using a Synergy H1 Microplate Reader.

### ATP assay.

The Molecular Probes ATP Determination Kit (Invitrogen) was used. iHeps were collected via trypsinization before trypan blue assay to normalize cell number. Upon centrifugation and removal of supernatant, cells were lysed and centrifuged again to collect lysate supernatant. Supernatant was utilized for ATP assays as per the manufacturer’s instructions. Standards of various dilutions were prepared to obtain a standard curve. Luminescence readings of the samples were performed using the Synergy H1 Microplate Reader.

### Flow cytometry.

Live cells were used to assess intracellular calcium levels, MMP, and mitochondrial superoxide production. Cells were trypsinized, and cell pellets were washed with PBS. Intracellular free calcium was quantified using Fluo-4 (Invitrogen). MMP was quantified using TMRM (Sigma-Aldrich), and mitochondrial superoxide production was quantified using MitoSOX (Invitrogen).

Fixed cells were used to assess intracellular SMN (sc-32313, Santa Cruz Biotechnology), SDHB (sc-271548, Santa Cruz Biotechnology), MT-CO1 (ab154477, Abcam), and HADHA (sc-374497, Santa Cruz Biotechnology) protein levels. Detached iHeps were incubated for 30 minutes with 3.7% paraformaldehyde before permeabilization for 30 minutes. Blocking was done with 1% BSA before a 30-minute incubation with SMN, SDHB, MT-CO1, and HADHA antibodies.

Measurements were performed on Cytoflex LX flow cytometer (Beckman Coulter Life Sciences) or LSRFortessa cell analyzer (BD Biosciences), using 1,000,000 cells per sample. However, only 10,000 events per sample were recorded. Raw data were processed using CytoFlex and FlowJo version 10.5.3.

### Western blot.

Cells were lysed with Pierce RIPA Lysis and Extraction buffer (Thermo Fisher Scientific). Supernatant was collected after centrifugation at 13,000*g* and 4°C for 20 minutes. Protein concentration was determined using a bicinchoninic acid (BCA) protein assay kit (Sigma-Aldrich). Samples were then heated at 95°C for 5 minutes before being loaded and resolved in precast SDS-polyacrylamide gels (Bio-Rad). Proteins were electrotransferred to a nitrocellulose membrane (Bio-Rad) in transfer buffer containing 48 mM Tris-HCl, 39 mM glycine, 0.037% SDS, and 20% methanol, at 4°C for 1 hour. Nonspecific binding to the membrane was blocked with 2.5% nonfat milk in TBS (20 mM Tris-HCl, 150 mM NaCl, and 0.1% Tween 20) for 1 hour at room temperature.

Membranes were incubated overnight (for at least 16 hours) at 4°C with either β-actin (A5441, Sigma-Aldrich) or SMN antibody in TBS containing 5% BSA at the dilutions specified by the manufacturer. Binding of primary antibodies was followed by incubation with secondary goat anti–mouse IgG–HRP antibody (32430, Thermo Fisher Scientific) in 2.5% nonfat milk for 1 hour at room temperature. The blots were visualized with SuperSignal West Femto Maximum Sensitivity Substrate (Thermo Fisher Scientific) using an iBright Imaging system (Invitrogen). Blot images were processed using ImageJ analysis software.

### RT-qPCR.

TRIzol Reagent (Invitrogen) for RNA extraction was utilized as per manufacturer’s instructions. cDNA was synthesized from reverse transcription of 1,000 ng of RNA (High-Capacity cDNA Reverse Transcription Kit; Applied Biosystems). Reverse transcription was performed in a T-Personal Thermocycler (Biometra) with conditions of 25°C for 10 minutes, 37°C for 120 minutes, followed by 85°C for 5 minutes. RT-qPCR was carried out to quantify mRNA expression of various target genes using SYBR Green Gene Expression Assay Probes (Applied Biosystems) and SYBR Green Universal PCR Master Mix (Applied Biosystems). *GAPDH* was used as the housekeeping gene for both nuclear and mitochondrial encoded genes of interest. The RT-qPCR was performed in the 7500 Real-time PCR System (Applied Biosystems) with conditions of 95°C for 10 minutes, followed by 95°C for 15 seconds and 60°C for 1 minute, for 40 cycles. Subsequently, the relative mRNA expression for the respective genes of interest was quantitated via the comparative CT (ΔΔCT) method. Relevant qPCR quality controls are presented in Supplemental Table 3. All primer sequences can be found in Supplemental Table 2.

### Proteomics.

Cell pellets of WT and SMA iHeps were harvested on day 24, prepared appropriately, and sent for tandem mass spectrometry analysis. Proteomic data from the samples were acquired using an Orbitrap Fusion Eclipse mass spectrometer (Thermo Fisher Scientific) in data-dependent mode. Detailed descriptions of sample preparation and tandem mass spectrometry analysis can be found in Supplemental Methods.

### Statistics.

For all groups, the results are presented as mean ± standard deviation (SD). Bar graphs were processed using GraphPad Prism 9.3.1 software. Comparisons involving 2 sample groups were performed using unpaired, 2-tailed Student’s *t* test. Comparisons involving 3 or more sample groups were performed using 1-way ANOVA test with Tukey’s multiple-comparison test. A *P* value of less than 0.05 was considered to be statistically significant. Outliers were assessed using the ROUT test with a maximum false discovery rate (FDR) of 1%. Identified outliers were then excluded from all statistical analyses.

### Study approval.

Study was approved by the Human Biomedical Research Office, Agency for Science, Technology and Research, Singapore and Boston Children’s Hospital Clinical Study of Spinal Muscular Atrophy protocol number 05-02-028.

### Data availability.

All data can be requested from the corresponding author. The proteomics data set is publicly available in the Japan ProteOme Standard Repository (jPOSTrepo) with the identifier PXD045401. Raw data underlying the results of this study can be found in the Supporting Data Values file.

## Author contributions

CJJY, BTD, and WYO designed the research studies. DMKL, YKN, LCW, HWLK, TZ, ZJK, TT, GN, RMS, WYO, and CJJY conducted experiments and acquired and analyzed data. RMG, SYN, AKKT, and LLR provided reagents. DMKL, YKN, LCW, GN, RMG, SHP, LLR, BTD, and CJJY wrote the manuscript. Co–first authors DMKL and YKN contributed to conducting experiments, acquiring data, analyzing data, and writing the manuscript. DMKL supervised YKN on performing experiments and is therefore listed first.

## Supplementary Material

Supplemental data

Unedited blot and gel images

Supporting data values

## Figures and Tables

**Table 1:**
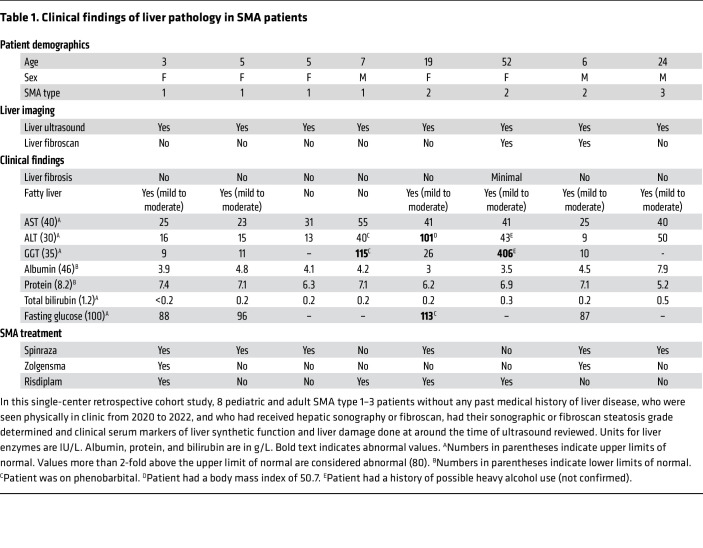
Clinical findings of liver pathology in SMA patients

**Table 2:**
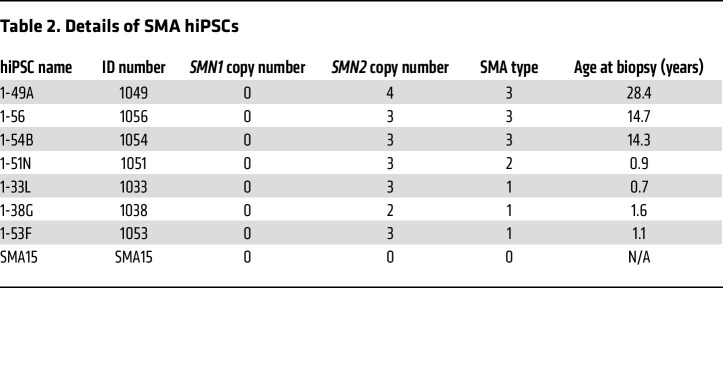
Details of SMA hiPSCs

**Table 3:**
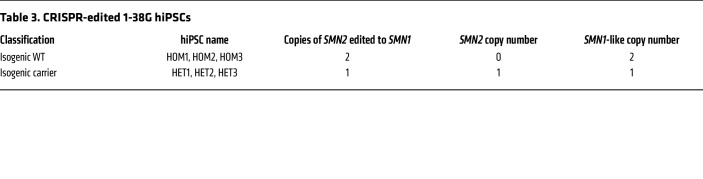
CRISPR-edited 1-38G hiPSCs

## Version 1 05/09/2024

In-Press Preview

## Version 2 06/17/2024

Electronic publication
