# Supplementary material for: Hepatocyte-intrinsic SMN deficiency drives metabolic dysfunction and liver steatosis in spinal muscular atrophy
Source: J Clin Invest. 2024 May 9;134(12):e173702. doi: 10.1172/JCI173702 (PMC11178536; doi:10.1172/JCI173702)
Supplement: Supplemental data [file jci-134-173702-s125.pdf]

Supplemental Information

Hepatocyte-intrinsic SMN deficiency drives metabolic dysfunction and liver steatosis in spinal muscular atrophy

Damien Meng Kiat Leow [1], Yang Kai Ng [1, 2], Loo Chien Wang[2], Hiromi W. L. Koh[2], Tianyun Zhao[2], Zi Jian Khong[2], Tommasco Tabaglio[2], Gunaseelan Narayanan[3], Richard M. Giadone[4], Radoslaw M. Sobota[2], Shi Yan Ng[1, 2, 5], Adrian Kee Keong Teo[1, 2], Simon H. Parson[6], Lee L. Rubin[4], Wei YiOng [1], Basil T. Darras[7], Crystal J. J. Yeo[*2,3, 5-9]

[1] Yong Loo Lin School of Medicine, National University of Singapore, Singapore 117597,Republic of Singapore

[2] Institute of Molecular and Cell Biology (IMCB), Agency for Science, Technology and Research (A*STAR), 61 Biopolis Drive, Proteos, Singapore 138673, Republic of Singapore

[3] Duke-National University of Singapore Medical School, Singapore 169857, Republic ofSingapore

[4] Department of Stem Cell and Regenerative Biology, Harvard University, Cambridge MA02138, USA

[5] National Neuroscience Institute, Singapore 308433, Republic of Singapore.

[6] Institute of Education in Healthcare and Medical Sciences, School of Medicine, MedicalSciences and Nutrition, University of Aberdeen, Aberdeen, AB51 7HA, Scotland

[7] Department of Neurology, Boston Children's Hospital, Harvard Medical School, Boston,Massachusetts, USA.

[8] Department of Neurology, Feinberg School of Medicine, Northwestern University, Chicago, IL, USA.

[9] Lee Kong Chian School of Medicine, Nanyang Technological University, Singapore 308232,Republic of Singapore.

Contents

Supplemental Information 1
Hepatocyte-intrinsic SMN deficiency drives metabolic dysfunction and liver steatosis in spinal muscular atrophy 1
Supplemental Methods 3
CRISPR/Cas9 Genome Editing 3
RT-qPCR Primers and Primer Efficiency 5
Oil Red O Image Analysis 7
Proteomics 8
Confocal imaging of iHeps 10
Supplemental Figures 12
Supplemental Figure 1 12
Supplemental Figure 2 13
Supplemental Figure 3 14
Supplemental Figure 4 15
Supplemental Figure 5 16
Supplemental Figure 6 17
Supplemental Figure 7 18
Supplemental Figure 8 19
Supplemental Figure 9 20
Supplemental Figure 10 21
Supplemental Figure 11 22
Supplemental Figure 12 23
Supplemental Figure 13 24
Supplemental Figure 14 25
Supplemental Figure 15 26
References 27
Supplementary Table S4 28

Supplemental Methods

CRISPR/Cas9 Genome Editing

Electroporation of sgRNA

70% confluent culture was used for electroporation. Cells were dissociated with TrypLE solution(Gibco) to obtain single cell suspension, and viability determined using Trypan Blue staining. 37.5 pmole sgRNA (IDT), 1 µg WT Cas9 protein (TrueCut™ Cas9 Protein v2; Invitrogen) and 1.25 µlR buffer (Neon transfection kit, Invitrogen) was mixed and incubated at room temperature for 20minutes to form RNP complexes. 0.5 µg ssODN donor (IDT) was then added and RNP complexeswere electroporated into 200,000 cells using the Neon transfection system (Invitrogen) following manufacturer's instructions. sgRNA spacer sequence TACAGGGTTTTAGACAAAAT was used. ssODN sequence TGTGAGCACCTTCCTTCTTTTTGATTTTGTCTGAAACCCTGTAAGGAAAATAAAGG AAGTTAAAAAAAATAGCTATATAG was used. Electroporated cells were seeded between 500 to 5,000 cells/cm^2. On Day 3 post-electroporation, genomic DNA was extracted and editing efficiency was checked in the cell pool through PCR and Sanger sequencing. Genomic DNA was isolated using Quickextract™ DNA extraction solution (QE09050, Lucigen). PCR was performedusing the Q5® high-fidelity PCR kit (M0491L, NEB), following manufacturer's instructions. Sanger sequencing was outsourced to Bio Basic Asia Pacific Pte Ltd.

Isolating clonal colonies

On Day 5 post-electroporation, clonal colonies were transferred individually to each well in a 24-well dish. Colonies were expanded for a week before genomic DNA was isolated using Quickextract™ DNA extraction solution (QE09050, Lucigen).

Genotyping of clones

Genomic DNA was used for PCR and Sanger sequencing the region of interest. PCR forward primer GGTCTCAAGTGATCCCCCTACC, reverse primer TCTGCTGGTCTGCCTACTAGTG and sequencing primer GCATGAGCCACTGCAAGAAAAC were used. Sanger sequencing data of clones were analyzed using online tool ICE Analysis v2.0 (https://ice.synthego.com/#/) to identify corrected clones.

Off-target analysis in clones

The top 10 off-target sites for the sgRNA (predicted by Benchling) were Sanger sequenced to check for off-target effects. Sequencing data were analyzed using online tools ICE Analysis v2.0 (https://ice.synthego.com/#/). The primers used for amplification and sequencing are listed in **Table S1**.

Cryopreservation

Cells were manually cut into clumps using a pipette tip. Clumps were then scraped using a cell scraper and transferred into cryovials. Cell clumps were short spun and supernatant removed. Cell clumps were resuspended in Cryostor® freezing medium (Stemcell Technologies). Cells were kept at -80°C overnight and transferred to liquid nitrogen for long-term storage.

Supplementary Table S1. Sequencing and PCR primers for off-target sites

Site No.	Sequence	PAM	Chromosome	Strand	Position	Sequencing Primer	PCR Forward Primer	PCR Reverse Primer
1	AGTCTGTGTAAAACCCTGTA	AGG	chr1	1	107759751	ACTCCATAGAATTTTGAGAGGG	ACTATGTTTGGCACAGAGAAGCC	AGTTTCCAAGGCATCCTGGAAC
2	ACTTGGTCTGAAACCCTGTA	TAG	chr14	-1	66452231	TGTCTTAAGAGAACCTTTGACTG	TTGCCTCTTACCAGAACCCCAG	TGCCCTGCTCTGCTTTCAAATC
3	ATTCAGTCTTAAACCCTGTA	GGG	chr4	-1	164954141	TCCTGGTGCAATCCTCACTCAG	CACCCCATCACAACCATTCTGC	CATGCTGCCAACCATGTGTAGG
4	AGCCTGTGTAAAACCCTGTA	GAG	chr2	-1	241204863	GTGTCCAGAGTCTAGGGCAGAG	TTTCACAGCAGTCACTTTGGCC	AGGGAACACTCACGTGGGTATG
5	ATCTTGTCTAAAAACCTGTA	TGG	chr3	1	99910195	GGGGCAAAATGTGTATTCAGAGC	GTCTGCAACCCCTTTCCTGTTC	AACATGCATCTGCCACTTCACC
6	TTTTTGACTATAACCCTGTA	TGG	chrX	-1	117007482	AGCACAAACACTTGACACATTCC	TCATCACCACAATGCATGCAGG	CCTCTTGGTGCCTAGCAGAAAC
7	CATTTGGGTAAAACCCTGTA	TAG	chrX	1	12823237	TGACTTTTAACAGGTACAGGTG	AGTGTCCAGCAAGCCTACTCTG	CCCCCAAAATGTGTCAGTGTCC
8	ATTTTGTGTGAAGCCCTGTA	AGG	chr3	-1	27994872	TCTCTGAAACTTTAATGTGACTTG	ACTTGCACTAGAGGAAGTCTAAATTCC	TGACGAGTTAGTGGGTGCAGTG

9	ATTCT GGGTT AAACC CTGTA	TGG	chr6	-1	147690888	CAACAAA GTATGGT GGAAATT CAG	GGAGTTCA AGAAAAG CCTGGCC	AGCTGGA AGGAAAC AAAGGCAC
10	CATTT GTCTC AAACC CTGTG	GAG	chr20	-1	52447631	CCTACTG CAGTCCT ACACACA G	AGTTCTTC CCATGTCT CCCAGC	GCTGGGAT TACAAGC ATGAGCC

RT-qPCR Primers and Primer Efficiency

Supplementary Table S2. Primers used for RT-qPCR.

S/N	Primers (*Homo Sapiens*)	Sequences (5' to 3')
1	*GAPDH*	Forward: GCAAATTCCATGGCACCGT<br>Reverse: GCCCCACTTGATTTTGGAGG
2	*SDHA*	Forward: GAGATGTGGTGTCTCGGTCCAT<br>Reverse: GCTGTCTCTGAAATGCCAGGCA
3	*SDHB*	Forward: GCAGTCCATAGAAGAGCGTGAG<br>Reverse: TGTCTCCGTTCCACCAGTAGCT
4	*SDHC*	Forward: CTGTTGCTGAGACACGTTGGT<br>Reverse: ACAGAGGACGGTTTGAACCTA
5	*MT-CO1*	Forward: CCGTCCTAATCACAGCAGTCCTA<br>Reverse: TGAGGTTGCGGTCTGTTAGTAGT
6	*MT-CO2*	Forward: CCGCCATCATCCTAGTCCTCAT<br>Reverse: GATCGTTGACCTCGTCTGTTATGT
7	*ATP5A*	Forward: TTTGAATCCCTATGAAGCGTT<br>Reverse: CCTTGGGTATTGCTTAATCGACC
8	*MT-ATP6*	Forward: TGATCATTCTATTTCCCCCTCT<br>Reverse: GTCATTGTTGGGTGGTGATT
9	*ALB*	Forward: GTTGCATGAGAAAACGCCAGT<br>Reverse: GTCGCCTGTTCACCAAGGAT
10	*ASGR1*	Forward: GAGAGAGACGTTCAGCAACTTC<br>Reverse: GGGACTCTAGCGACTTCATCTT
11	*ASGR2*	Forward: GCTTTCAGCAACTTCTCCTC<br>Reverse: TTCTCCAGCTTGGCTCCTA
12	*APOE*	Forward: TCGCTTTTGGGATTACCT<br>Reverse: CTCCTTCATGGTCTCGTC
13	*APOA1*	Forward: ATGTGTCCCAGTTTGAAG<br>Reverse: CCCTCTGTCTCCTTTTCC
14	*F2*	Forward: TTGCTGCATGTCTGGAAGGTA<br>Reverse: GGATGGGTAGTGGAGTTGATT
15	*SERPINA1*	Forward: CCAAGGCCGTGCATAAGG<br>Reverse: GGCCCCAGCAGCTTCAG
16	*HP*	Forward: TTTTTGCAGTGGACTCAGG<br>Reverse: GCGCAGTTTGTAGTAGTT
17	*HNF4A*	Forward: CTCCATCAATGCGCTCCT<br>Reverse: CTTCATGGACTCACACACATCT
18	*AFP*	Forward: AGTGAGGACAAACTATTGGCCT<br>Reverse: ACACCAGGGTTTACTGGAGTC
19	*OCT4*	Forward: AGAAGTGGGTGGAGGAAG<br>Reverse: ACGAGGGTTTCTGCTTTG

20	*NANOG*	Forward: ATGCCTCACACGGAGACTGT<br>Reverse: AGGGCTGTCCTGAATAAGCA
21	*SOX2*	Forward: GAAAGAAAGGGAGAGAAGTTTGAG<br>Reverse: GCAAACTGGAATCAGGATCAA
22	*CPT1A*	Forward: CCAGACGAAGAACGTGGTCA<br>Reverse: ATCTTGCCGTGCTCAGTGAA
23	*CPT2*	Forward: GTAGCACTGCCGCATTCAAG<br>Reverse: GCCATGGTACTTGGAGCACT
24	*ACSL1*	Forward: GACGAGCCCTTGGTGTATTT<br>Reverse: TTCATAGGGTTGGTCTGGTTTC
25	*ACAD1*	Forward: TGTGCCTATTGTGTAACAGAACCT<br>Reverse: TCCATTGGTGATCCACATCTTCTGAC
26	*ACOT1*	Forward: GGTGACCAAAGATGGCTATG<br>Reverse: TGACCTACCAGGAACAGGAA
27	*HADHA*	Forward: ACTGCTGTCCTCTTCAGCTCAA<br>Reverse: ACTGACTGAGCGAGGCATGA
28	*SCD1*	Forward: CCGGGAGAATATCCTGGTTT<br>Reverse: GCGGTACTCAACTGGCAGAGT
29	*SREBP1*	Forward: ACAGTGACTTCCCTGGCCTAT<br>Reverse: GCATGGACGGGTACATCTTCAA
30	*HMGCS1*	Forward: TGTACACATCTTCAGTATATGGTTCCC<br>Reverse: AAGAAAACACTCCAATTCTCTTCCCT
31	*PCK2*	Forward: AGCCTCTTCCACCTGGTGTT<br>Reverse: AATCGAGAGTTGGGATGTGC
32	*G6Pase*	Forward: GGGAAAGATAAAGCCGACCTAC<br>Reverse: CAGCAAGGTAGATTCGTGACAG
33	*FMO1*	Forward: ACCTGGCGGAAAAGGTGT<br>Reverse: CATGTTCTGAAAGCGTGTCAT
34	*FMO3*	Forward: TTCCCACAGTTGACCTCC<br>Reverse: CCATTTGCGCTTTTTCTCC
35	*ACTB*	Forward: AGATGAGATTGGCATGGCTTTA<br>Reverse: GGACTTCCTGTAACAACGCATC
36	*HPRT*	Forward: TGCTGAGGATTTGGAAAGGG<br>Reverse: ACAGAGGGCTACAATGTGATG

Supplementary Table S3. Evaluation of RT-qPCR primer efficiencies.

S/N	Gene Name	PCR Primer Efficiency (CT range)	R^2	Intra-assay Variation (% CV)	Range of CT values across all iHeps/ positive control/ negative control	
					Min.	Max.
1	*GAPDH*	91% (20.2-35.0)	0.99	0.8	15.2	30.6
2	*BETA-ACTIN*	99% (19.0-36.5)	0.99	0.6	18.0	22.4
3	*HPRT*	95% (19.9-34.9)	0.98	0.9	19.4	26.9
4	*SDHA*	92% (25.5-37.0)	0.98	0.4	19.8	35.2
5	*SDHB*	91% (24.6-36.3)	0.98	1.0	19.2	33.2
6	*SDHC*	109% (25.5-35.8)	0.98	0.6	19.2	36.7
7	*MT-ATP6*	93% (17.9-35.8)	0.99	0.5	11.3	23.6
8	*ATP5A*	98% (27.3-38.5)	0.98	0.7	20.6	31.5
9	*MT-CO1*	86% (15.8-35.1)	0.99	1.1	12.0	24.1
10	*MT-CO2*	87% (18.1-36.5)	0.99	0.4	12.6	24.5
11	*OCT4*	103% (21.1-35.2)	0.98	0.6	22.9	36.6

12	*SOX2*	110% (24.0-37.0)	0.98	0.2	27.4	39.6
13	*NANOG*	96% (24.5-35.7)	0.98	0.6	23.6	32.1
14	*ASGR1*	92% (24.5-35.9)	0.98	< 0.1	19.3	33.8
15	*ASGR2*	88% (24.4-35.1)	0.99	1.0	18.0	37.6
16	*ALB*	93% (23.9-34.3)	0.98	1.0	14.6	35.6
17	*HNF4A*	94% (27.2-34.7)	0.98	0.6	22.5	39.1
18	*HP*	92% (27.8-38.0)	0.99	0.8	21.4	37.9
19	*AFP*	87% (23.0-38.3)	0.99	0.4	12.8	32.6
20	*F2*	110% (25.7-35.4)	0.98	0.6	20.5	38.6
21	*SERPINA1*	105% (18.4-38.6)	0.98	0.8	17.1	36.1
22	*ACAD1*	101% (28.6-35.8)	0.98	0.4	22.5	35.3
23	*CPT1A*	86% (28.0-38.6)	0.99	0.4	20.4	36.6
24	*CPT2*	93% (19.7-37.0)	0.98	0.5	23.5	38.0
25	*HADHA*	110% (24.3-37.4)	0.98	0.6	18.9	32.4
26	*APOE*	93% (26.1-37.2)	0.98	0.8	20.5	28.7
27	*APOA1*	91% (22.0-37.1)	0.99	0.5	17.3	38.2
28	*ACOT1*	91% (26.1-35.8)	0.98	0.6	20.7	36.5
29	*PCK2*	102% (24.8-35.5)	0.98	0.6	19.6	35.7
30	*ACSL1*	97% (27.7-35.1)	0.98	0.4	22.2	31.6
31	*SCD1*	94% (25.5-37.6)	0.98	0.1	17.9	25.3
32	*SREBP1*	88% (20.6-37.0)	0.98	1.1	28.3	39.7
33	*G6PASE*	94% (24.1-36.0)	0.98	0.7	25.2	39.2
34	*FMO1*	98% (27.4-37.0)	0.98	0.8	24.3	38.5
35	*FMO3*	91% (24.7-37.0)	0.98	1.1	29.8	39.9
36	*HMGCS1*	100% (24.9-37.0)	0.98	1.8	20.9	24.7

The amplification efficiency for each primer threshold cycle (Ct) and the logarithm of the initial cDNA concentrations were plotted to calculate the slope (S) of each primer pair. Standard curves were generatedfrom at least four dilution points for each primer pair. RT-qPCR reactions for each sample were run in duplicate, with standard deviations <0.85. Primer concentration (µM), standard deviation (SD), co-variance (*CV*), amplification efficiency (*E*) and correlation coefficient (R^2) * qPCR efficiency ($E = 10^{(-1/\text{slope})} - 1$) and correlation coefficient (R^2) were determined by standard curve by excel data.

Oil Red O Image Analysis

All histological analyses were done by a blinded researcher. Oil Red staining was measured using morphometry (ImageJ, version 2.7.0). Images taken with a microscope (three fields per sample) were randomly selected in ImageJ and transformed to black and white using "Image-type" submenu. Next, threshold function was applied using either the default or automatic settings to only choose the dark portion of the image (i.e., image area containing Oil Red staining). "Analyze- Analyze particles" submenu was used to calculate total Oil Red staining area. Cell counting per microscopic field was done by a blinded researcher based on haematoxylin stain. Total red intensity was normalised to cell numbers per microscopic field. The same image acquisition parameters and analysis methods were used for quantification of all samples.

Proteomics

Sample Preparation for Proteomics

Cell pellets were collected after 24 days of hepatocyte differentiation from human stem cells. Cell pellet samples were lysedusing 8 M urea in 50 mM Tris for protein denaturation followed by probe sonication (Vibracell, Sonics and Materials, Inc.) for 3 cycles of 15 s pulse at 30% amplitude. Total protein amount in all samples were quantified by bicinchoninic acid assay (BCA) measurement and 50 µg of protein was taken for downstream experiment. Samples were reduced using a final concentration of 10 mM tris (2-carboxyethyl) phosphine (TCEP) and alkylated using a final concentration of 55 mM 2-chloroacetamide (CAA) at 25 °C for 30 min in the dark. This was followed by digestion with 1.5 µg of endoproteinase LysC for 4 h and 1.5 µg trypsin overnight at 25 °C. Digestion was terminated by adding 1% (v/v)final concentration of trifluoroacetic acid (TFA) to the samples, followed by desalting with OasisHLB plate (1 cc/30 mg sorbent, Waters Corporation). The wells were activated with 100% (v/v) acetonitrile and equilibrated with 0.1% (v/v) formic acid in water. Peptides were then loaded ontothe wells and washed once with 0.1% (v/v) formic acid in water. Bound peptides were eluted with65% acetonitrile in 0.1% formic acid into a 2-ml deep well plate and transferred to 1.5-ml Eppendorf tubes later for drying.

Desalted peptides were resuspended with 100 mM triethylammonium bicarbonate (TEAB) and grouped as two sets to be labelled with isobaric tandem mass tags (TMT16-plex). The labelling was done for 2 h at 25 °C. Samples were then fractionated by high-pH reversed- phase at different concentrations of acetonitrile in 10 mM ammonium formate, pH (14%, 18%, 21%, 24%, 27%,32% and 60%). Eluted peptides were dried by centrifugal evaporation for MS analysis.

Tandem Mass Spectrometry Analysis

Dried samples were resuspended in 10 µl of 2% (v/v) acetonitrile containing 0.06% (v/v) trifluoroacetic acid and 0.5% (v/v) acetic acid and transferred to an autosampler plate. Online chromatography was performed in an EASY-nLC 1000 (Thermo Fisher Scientific) liquid chromatography system using single-column setup and 0.1% formic acid in water and 0.1% formicacid in 99% acetonitrile as mobile phases. Fractions were injected and separated on a reversed- phase C18 analytical column (Easy-Spray, 75 µm inner diameter × 50 cm length, 2 µm particle size, Thermo Fisher Scientific) maintained at 50 °C and using a 2-27% (v/v) acetonitrile gradientover 45 min, followed by an increase to 55% over the next 15 min, and to 95% over 5 min. The final mixture was maintained on the column for 5 min to elute all remaining peptides. Total run duration for each sample was 70 min at a constant flow rate of 300 nl/min.

Data was acquired using an Orbitrap Fusion Eclipse mass spectrometer (Thermo Fisher Scientific)using data-dependent mode. Samples were ionized using 2.1 kV and 300 °C at the nanospray source. Positively-charged precursor signals (MS1) were detected using an Orbitrap analyzer set to 60,000 resolution, automatic gain control (AGC) target of 400,000 ions, and maximum injectiontime (IT) of 100 ms. Precursors with charges 2-7 and having the highest ion counts in each MS1 scan were further fragmented using higher-energy collision-induced dissociation (HCD) at 42% normalized collision energy. Fragment signals (MS2) were analysed by the ion trap analyzer at anAGC of 75,000 and maximum IT of 50 ms. Precursors used for MS2 scans were excluded for 90 s to avoid re-sampling of high abundance peptides. The MS1–MS2 cycles were repeated every 3s until completion of the run.

Proteomics Data Analysis

Proteins were identified using Proteome Discoverer™ (v3.0, Thermo Fisher Scientific). Raw mass spectra were searched against human primary protein sequences retrieved from UniProt (11 June 2019). Carbamidomethylation on Cys and TMT16-plex wereas set as a fixed modification; deamidation of Asn and Gln, acetylation on protein N termini, and Met oxidation were set as dynamic modifications for the search. Trypsin/P was set as the digestion enzyme and was allowed up to three missed cleavage sites. Precursors and fragments were accepted if they had a mass error within 10 ppm and 0.06 Da, respectively. Peptides were matched to spectra at a false discovery rate (FDR) of 1% (strict) and 5% (relaxed) against the decoy database. Database search identified a total of 4,306 human protein groups within the search parameters used (see “Total protein ID” in **Supplementary Table S4**). Search results were exported and further processed using an in-house R script that utilizes *limma* for differential analysis, similar to a study by Ritchie *et al.*, 2015. For quantitative analysis, no imputation was performed for proteins that showed missing value in any of their TMT channels and they were removed from downstream processing. Median normalization was carried out across the samples to equalize the medians and eliminate potential differences arising from differing sample loading amounts (**Supplementary Fig. S13A**). Principal component analysis (PCA) was used to visualize the variability of the samples and determine outliers (**Supplementary Fig. S13B**). Normalized data was applied to *limma* R package for differential expression analysis using linear model. Statistical significance is defined at false discovery rate (FDR) < 0.05 and a fold-change requirement of equal to or greater than 1.5-fold difference is applied to differential proteins. For comparison sets where there were ≤ 200 proteins, it was not possible to adjust for multiple hypotheses and *p*-values < 0.01 were used for significant differences. Of the 6567 proteins in the cell pellet samples that showed significant differences in expression in at least one of the SMA

phenotypes (SMA0, SMA1, SMA2, or SMA3) compared to WT from the linear model analysis, we further investigated whether these proteins showed increasing or decreasing trends with known clinical severities. We fitted a linear regression on the expression of the proteins against the phenotypic groups as ordinal variables with increasing severity (from WT to SMA0) and tested the hypothesis if the slope is equal to zero, which would indicate that there is no correlation of the proteins with disease severity. At a stringent FDR of < 0.01, the analysis revealed 343 differentially-expressed proteins exhibiting trends correlating with progressive severity of the disease (**Supplementary Table S4**).

For the iHeps cell pellet samples, proteins with significant differences were visualized for their interactions using STRING database (https://string-db.org/). Briefly, the significantly different genes were submitted and matched to the *Homo sapiens* database to correctly identify available proteins. Default settings were used except for the following: meaning of network edges was set to "evidence" and "textmining" was removed from active interaction sources to keep experimentally-validated edges only with confidence cut-off at 0.400. *k*-means clustering was then performed to separate the network into three distinct clusters (**Fig. 3A** and **Supplementary Fig. S9A and S12**). Proteins in individual clusters were analyzed for Gene Ontology (GO) biological processes term enrichment using the ShinyGO app hosted by South Dakota State University (http://bioinformatics.sdstate.edu/go/, v0.77). The top 20 pathways were shown, and pathways were selected by their $-\log_{10}$(FDR) scores and sorted by fold enrichment in decreasing order (**Supplementary Fig. S6 - 8**).

Following ShinyGO analysis, it was found that mitochondria-related processes were enriched for Cluster 1 (**Figure 3A, red**) proteins. Following up on this, proteins from the 343 differentially-expressed list were matched to only those that are associated with mitochondria based on the subcellular localization on the UniProt database (1,293 mitochondrial proteins out of 20,424 proteins in the proteome) and mitochondrial pathway using MitoCarta3.0 mitochondrial pathway inventory (https://www.broadinstitute.org/mitocarta/). ShinyGO enrichment analysis was performed again for this subset of 54 proteins. Proteins directly involved in mitochondrial complex V (ATP synthase) and lipid homeostasis (Lipid transport, triglyceride accumulation, fatty acid oxidation and cholesterol metabolism) respectively based on UnitProt database, were also analysed using STRING database.

Confocal imaging of iHeps

Immunocytochemistry was employed to visualize mitochondrial density and mitochondrial membrane potential. WT BJ iPSCs and SMA1 GM24468 iPSCs (Zero *SMN1* copy number, two

SMN2 copy number and age of biopsy at age of three) were (Coriell, New Jersey, United States) were seeded onto 13mm coverslips in respective 6-well plates prior to hepatocyte differentiation illustrated in **Figure 1**. After at least 24 days of differentiation, iHeps were washed thrice with 1X PBS before incubationwith 50nM of MitoTracker^{TM} Green FM (Thermo Fisher Scientific) and tetramethylrhodamine methyl ester perchlorate (TMRM) (Sigma Aldrich) for 15min. Three 1X PBS washes were performed before addition of ProLong™ Gold Antifade Mountant with DAPI (Thermo Fisher Scientific) and visualized with Olympus FV3000 confocal microscope (Tokyo, Japan). Percentages of normalized TMRM intensity to MitoTracker^{TM} (TMRM/MitoTracker^{TM}) were plotted using ImageJ v1.51 and GraphPad prism 9 software. The same image acquisition parameters and analysis methods were used for quantification of all samples similar to Stuhr et al.[1]

Supplemental Figures

Supplemental Figure 1

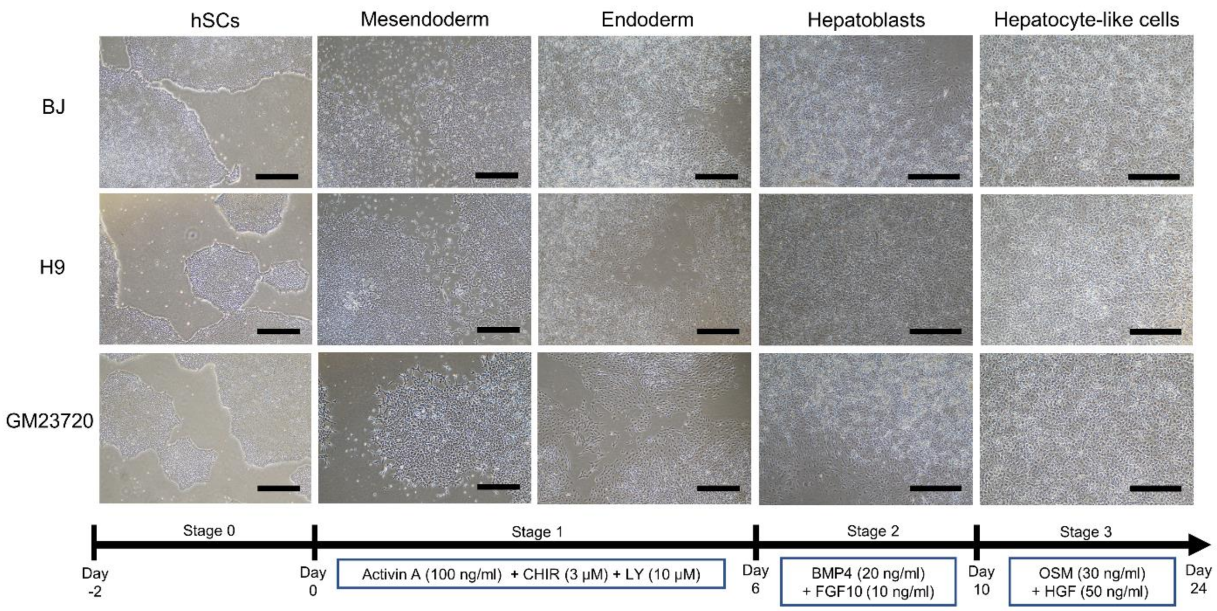

Figure S1. Differentiation of patient-derived stem cells to Day 24 iHeps. Morphological representation of WT stem cells through the iHep differentiation with schematic of differentiation over 24 days. Day 0: Stem cells; Day 2: Mesendoderm; Day 6: Definitive endoderm; Day 10: Hepatoblasts; Day 24: Hepatocyte-like cells (iHeps). Scale bar for hSCs, mesendoderm and endoderm: 500 μm. Scale bar for hepatoblasts and hepatocyte-like cells: 250 μm.

Supplemental Figure 2

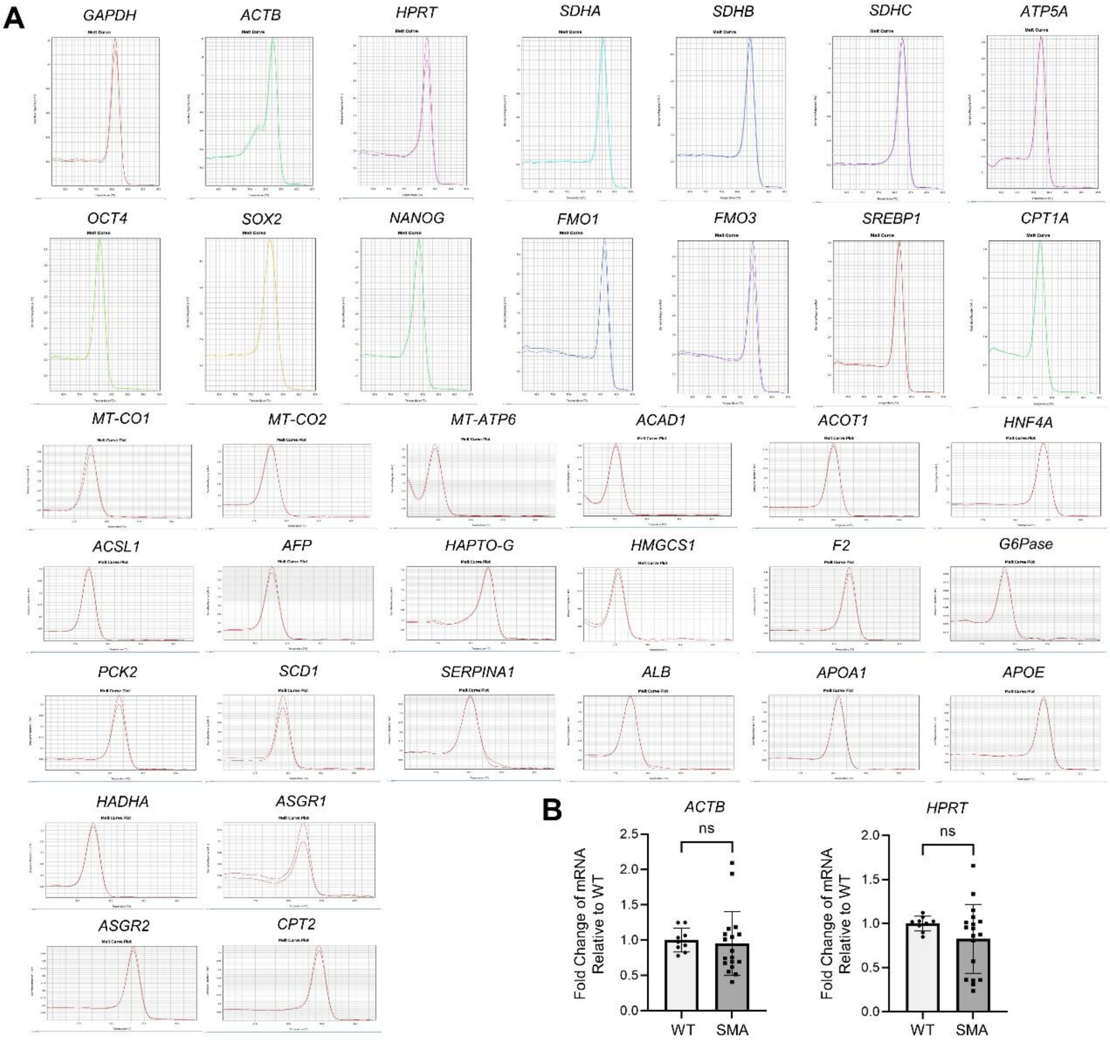

Figure S2. Melt curve analysis of RT-qPCR primers and housekeeping gene validation. (A) From the melt curves which plot for the derivative reporter against temperature, we observe that all our RT-qPCR primers were able to produce a single amplification product as seen from the single peaks. **(B)** We verified that two other housekeeping genes (*ACTB* and *HPRT*), when normalized to *GAPDH* mRNA expression, showed no significant difference between the WT and SMA sample groups. SMA: SMA3 and SMA1 iHep lines combined. WT n=9, SMA3 n=9, SMA1 n=9, each with three biological replicates, from three independent experiments. This demonstrates that *GAPDH* mRNA expression between the WT and SMA lines have no significant difference and is appropriate as a housekeeping gene for the context of this study. Data were analyzed using unpaired two tailed Student's t-test. No outliers were detected using the ROUT test with a maximum false discovery rate (FDR) of 1%. Data are presented as mean (±SD). ns: non-significant.

Supplemental Figure 3

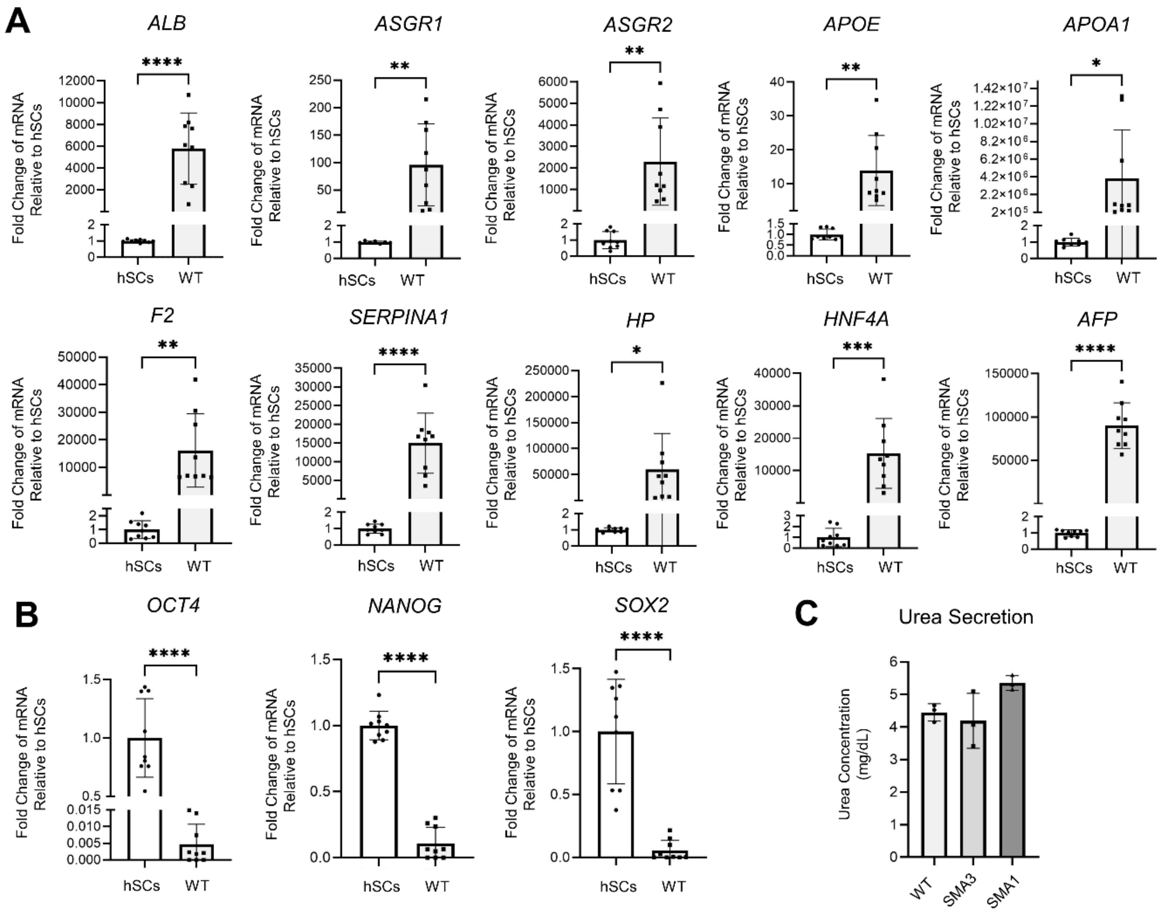

Figure S3. WT iHeps differentiation. (A) RT-qPCR analysis of hepatocyte biomarkers between Day 24 WT iHeps and Day 0 WT stem cells. For *ASGR1*, two outliers from hSCs were removed. For *ASGR1*, WT hSC n=7 and WT iHeps n=9. For all other hepatocyte biomarker genes, WT hSC n=9 and WT iHeps n=9. WT hSCs and WT iHeps data include three biological replicates each, and are from three independent experiments. **(B)** RT-qPCR analysis of stem cell biomarkers in Day 24 WT iHeps and Day 0 WT stem cells. WT hSCs n=9 and WT iHeps n=9, each with three biological replicates and from three independent experiments.. **(A - B)** For all RT-qPCR, fold change results were derived using the comparative ΔΔCt method. **(C)** Urea expression assay of Day 24 WT, SMA3 and SMA1 iHeps, via urea detection in conditioned cell culture media. WT n=3, SMA3 n=3, SMA1 n=3, (three biological replicates). **(A - C)** Data sets involving two sample groups were analyzed using unpaired two-tailed Student's t-test, while those with three or more groups were analyzed using One-Way ANOVA test with Tukey's multiple comparison test. Any outliers were detected and removed using the ROUT test with a maximum false discovery rate (FDR) of 1%. Data are presented as mean (±SD). * p-value < 0.05; ** p-value < 0.01; *** p-value < 0.001; **** p-value < 0.0001.

Supplemental Figure 4

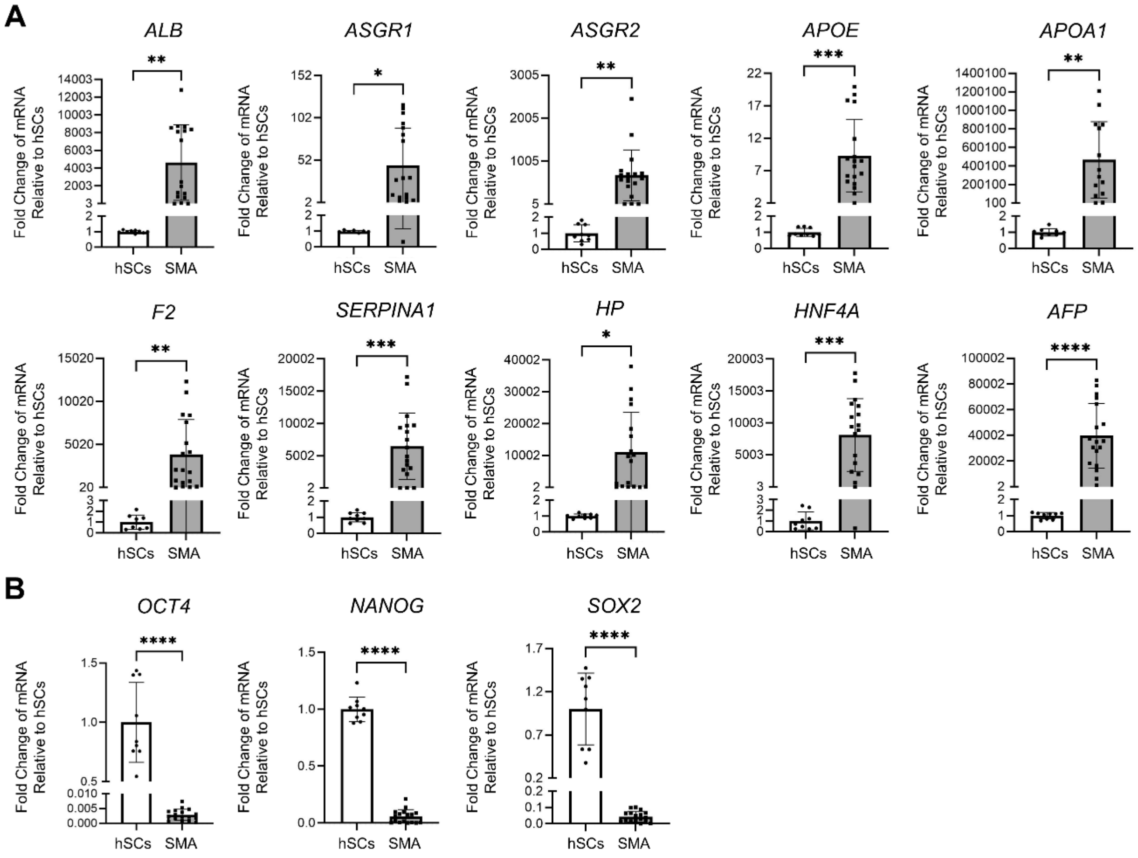

Figure S4. SMA iHeps differentiation. (A) RT-qPCR analysis of hepatocyte biomarkers between the WT stem cells (hSCs) and the SMA iHeps (SMA3 and SMA1 combined). For *ALB*, one outlier from SMA was removed, WT hSCs n=9, SMA3 iHeps n=9, SMA1 iHeps n=8. For *ASGR1*, two outliers from hSCs were removed, WT hSCs n=7, SMA3 iHeps n=9, SMA1 iHeps n=9. For *APOA1*, four outliers from SMA were removed, WT hSCs n=9, SMA3 iHeps n=7, SMA1 iHeps n=7. For all other hepatocyte biomarker genes, WT hSCs n=9, SMA3 iHeps n=9, SMA1 iHeps n=9 **(B)** RT-qPCR analysis of stem cell biomarkers between the WT stem cells (hSCs) and the SMA iHeps (SMA3 and SMA1 combined). For *OCT4*, *NANOG* and *SOX2*, one outlier from SMA was removed For *OCT4* and *NANOG*, WT hSCs n=9, SMA3 iHeps n=9, SMA1 iHeps n=8, and for *SOX2*, WT hSCs n=9, SMA3 iHeps n=8, SMA1 iHeps n=9. **(A – B)** Data is from three independent experiments, each with three biological replicates per sample group. Data was analyzed using unpaired two-tailed Student’s t-test. Any outliers were detected and removed using the ROUT test with a maximum false discovery rate (FDR) of 1%. Data are presented as mean (±SD). * p-value < 0.05; ** p-value < 0.01; *** p-value < 0.001; **** p-value < 0.0001.

Supplemental Figure 5

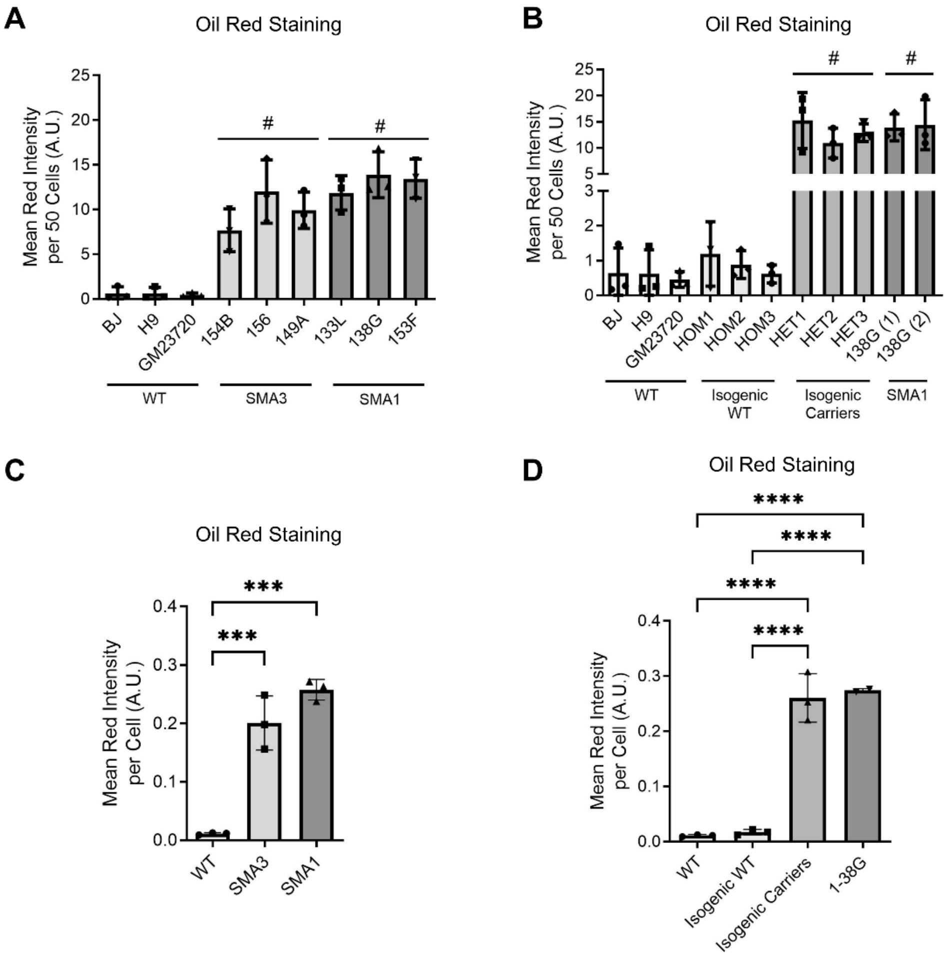

Figure S5. Oil Red O staining of Day 24 WT and SMA iHeps. Oil red staining positively correlates with levels of neutral triglycerides and lipids in iHeps. **(A)** Mean red intensity staining per every 50 cells (A.U.) between the individual WT, SMA3 and SMA1 iHep lines. # represents statistically significant difference between the respective cell lines with respect to WT lines. **(B)** Mean red intensity staining per every 50 cells (A.U.) between the individual WT, isogenic WT, isogenic carriers and 1-38G iHep lines. # represents statistically significant difference between the respective cell lines with respect to WT lines and isogenic WT lines. **(A - B)** For all cell lines, n=3, and are representative of three independent experiments. **(C)** Mean red intensity staining per cell (A.U.) of the respective iHep lines under WT (n=3), SMA3 (n=3) and SMA1 (n=3). Data is representative of three independent experiments. **(D)** Mean red intensity staining per cell (A.U.) of the respective iHep lines under WT (n=3), Isogenic WT (n=3), Isogenic carriers (n=3) and SMA1 (n=2). Data is representative of three independent experiments. **(A - D)** Data were analyzed using One-Way ANOVA test with Tukey's multiple comparison test. No outliers were detected using the ROUT test with a maximum false discovery rate (FDR) of 1%. Data are presented as mean (±SD). *** p-value < 0.001; **** p-value < 0.0001.

Supplemental Figure 6

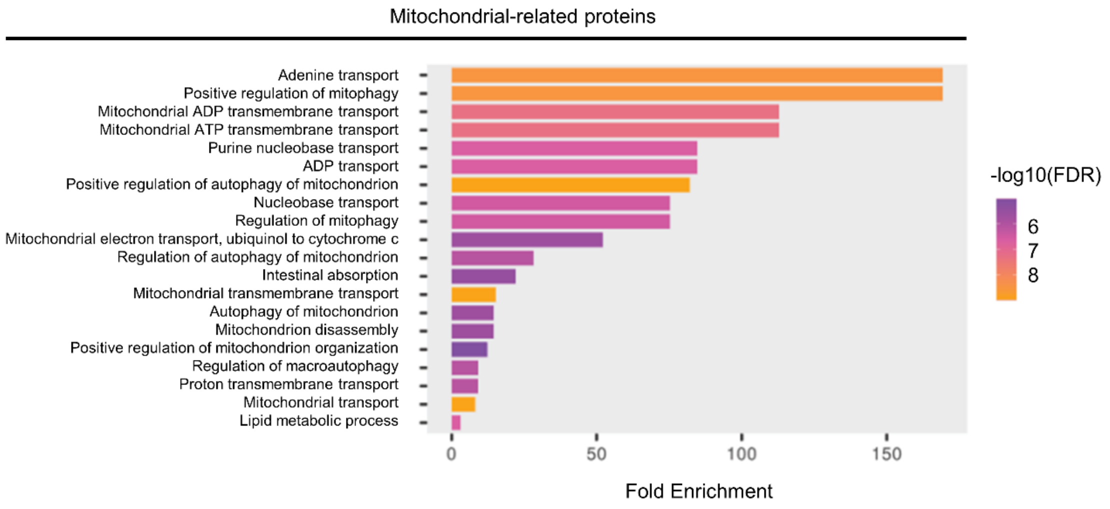

Figure S6. Proteins in Cluster 1, mitochondrial-related processes, subjected to term enrichment analysis using the ShinyGO app. Only the top 20 pathways associated with the cluster are shown and pathways were selected by their –log10(FDR) scores and sorted by fold enrichment.

Supplemental Figure 7

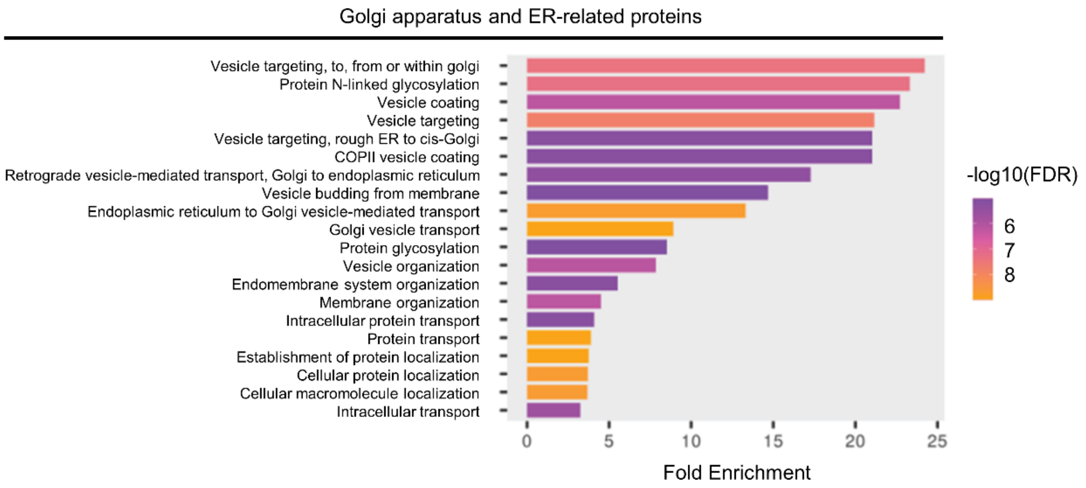

Figure S7. Proteins in Cluster 2, golgi apparatus and endoplasmic reticulum (ER) related processes, subjected to term enrichment analysis using the ShinyGO app. Only the top 20 pathways associated with the cluster are shown and pathways were selected by their –log10(FDR) scores and sorted by fold enrichment.

Supplemental Figure 8

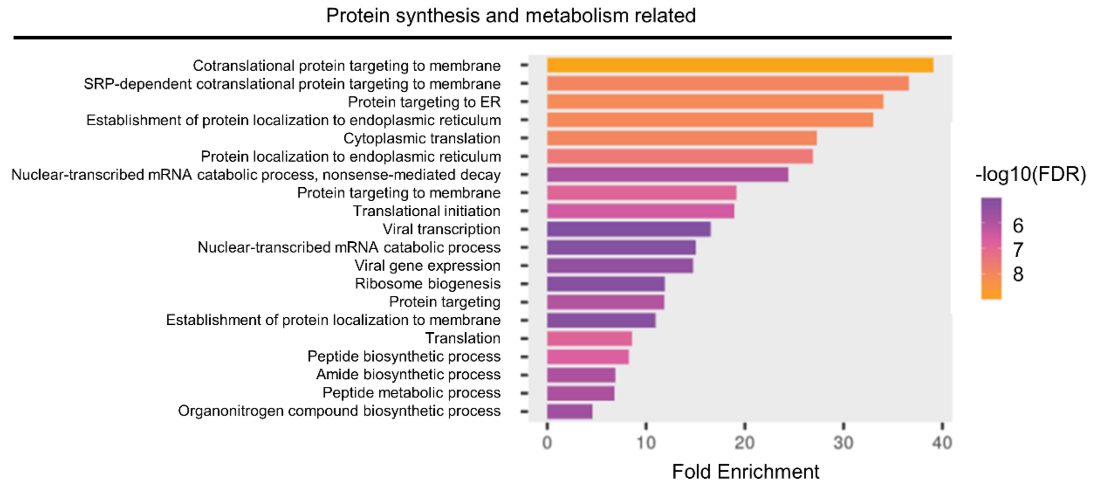

Figure S8. Proteins in Cluster 3, protein synthesis and metabolism related processes, subjected to term enrichment analysis using the ShinyGO app. Only the top 20 pathways associated with the cluster are shown and pathways were selected by their –log10(FDR) scores and sorted by fold enrichment.

Supplemental Figure 9

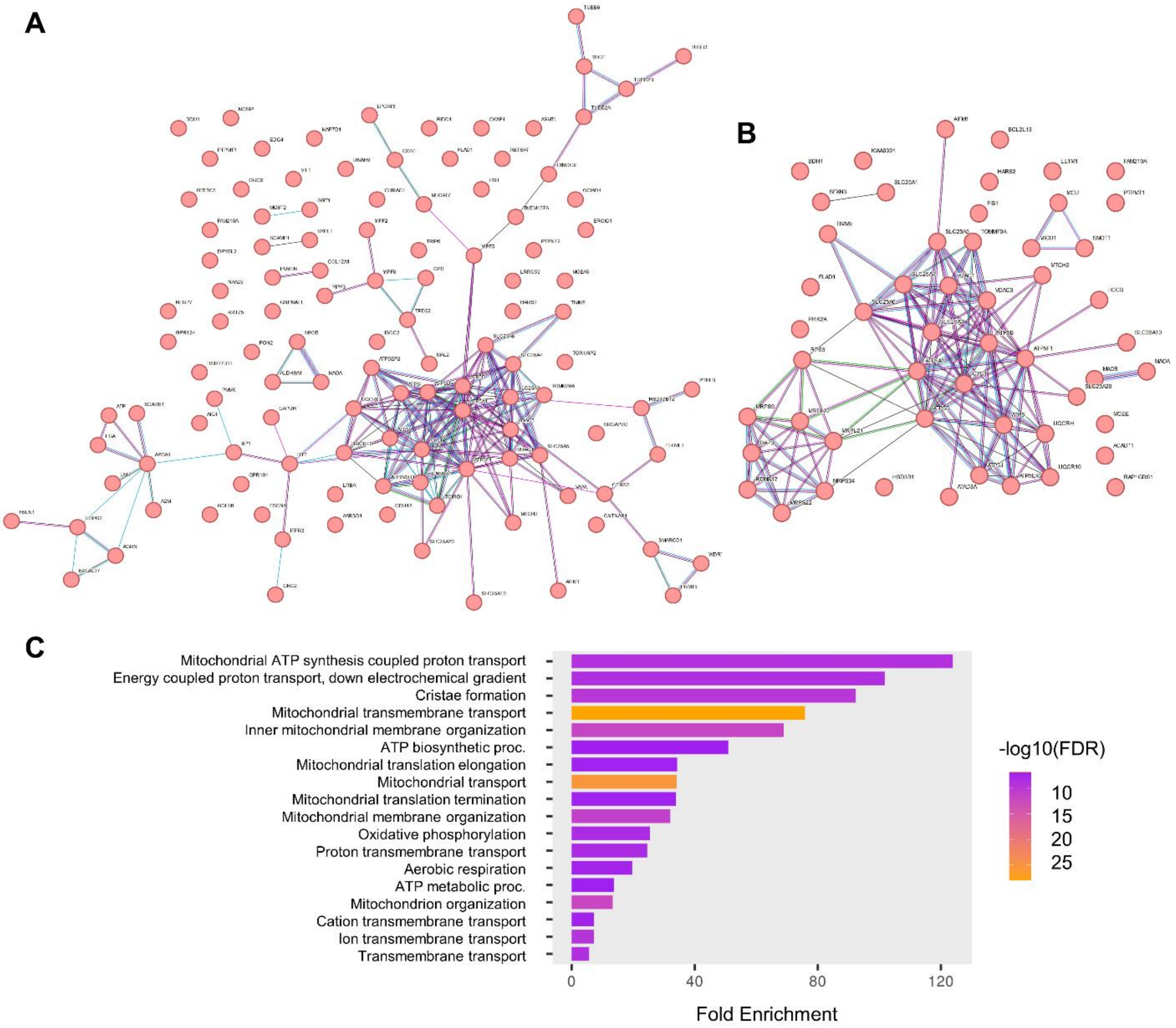

Figure S9. Network analysis of Day 24 WT and SMA iHeps. (A) STRING analysis of proteins in Cluster 1 (red) suggested that there were mitochondrion-related processes in the interaction network. **(B)** STRING analysis of a subset of proteins in Cluster 1 involved in mitochondrial related processes in the interaction network using the UniProt database. **(C)** The top 20 pathways enriched by the subset of proteins in **(B)** selected by their –log10(FDR) scores and sorted by fold enrichment.

Supplemental Figure 10

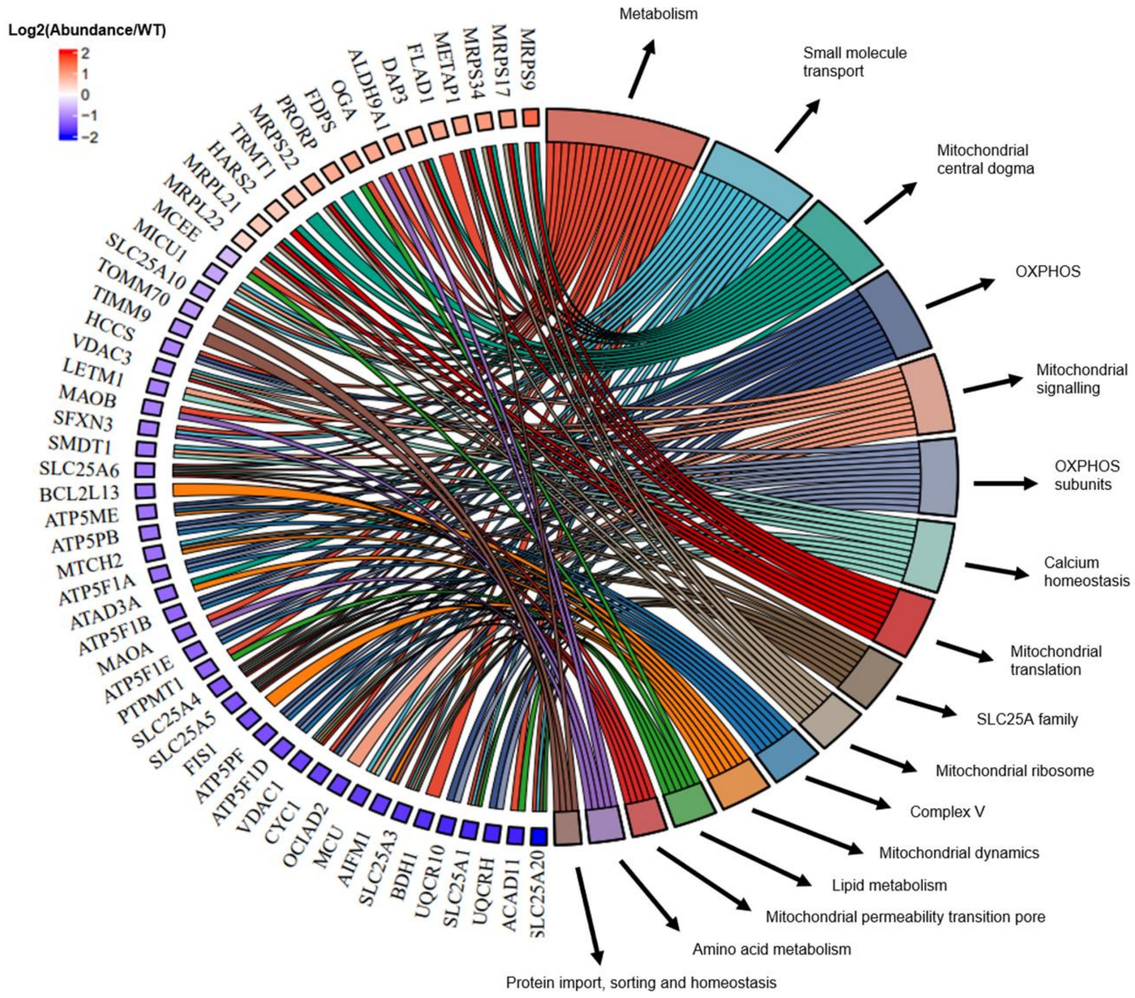

Figure S10. Circos plot associating mitochondrial protein hits with mitochondrial pathways. Protein hits associated with mitochondria based on the subcellular localization on the UniProt database and mitochondrial pathway using MitoCarta3.0 mitochondrial pathway inventory were used to generate Circos plot.

Supplemental Figure 11

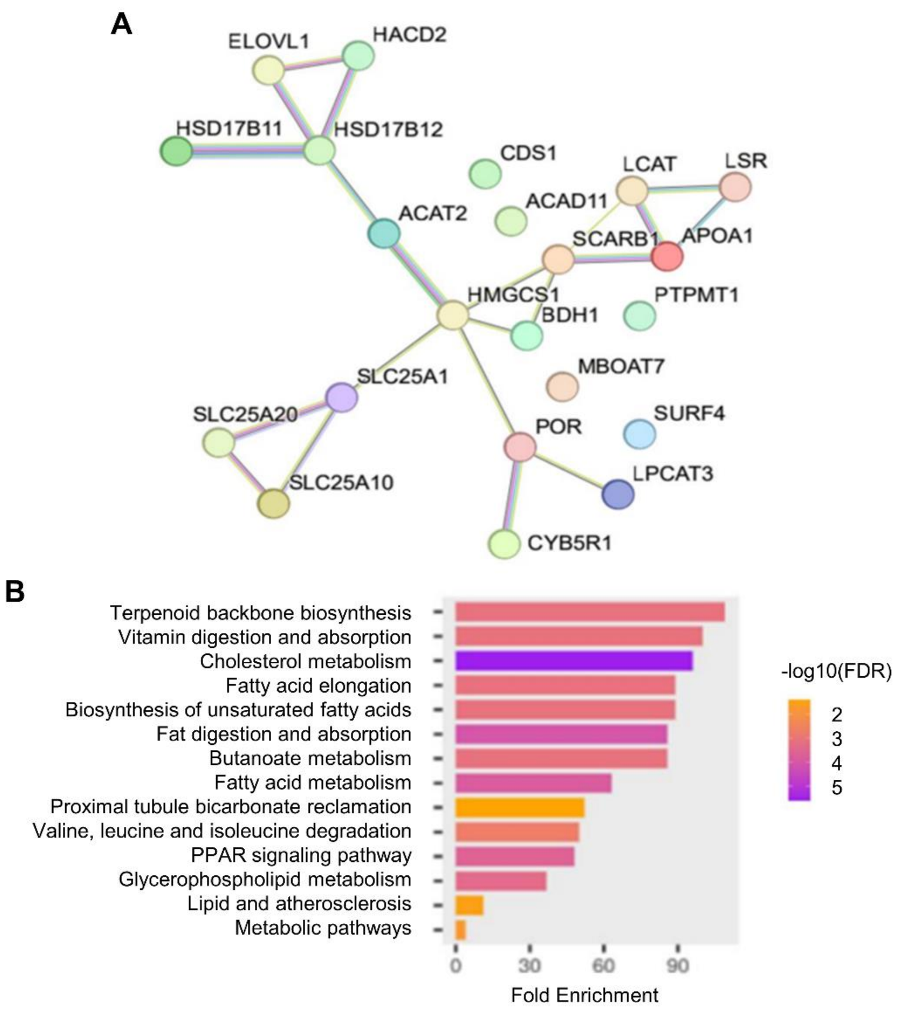

Figure S11. Network analysis of Day 24 WT and SMA iHeps. (A) STRING analysis of proteins involved in lipid homeostasis including lipid transport, triglyceride accumulation, fatty acid oxidation and cholesterol metabolism in the interaction network using the UniProt database. **(B)** The top 20 pathways were shown out of the top pathways selected.

Supplemental Figure 12

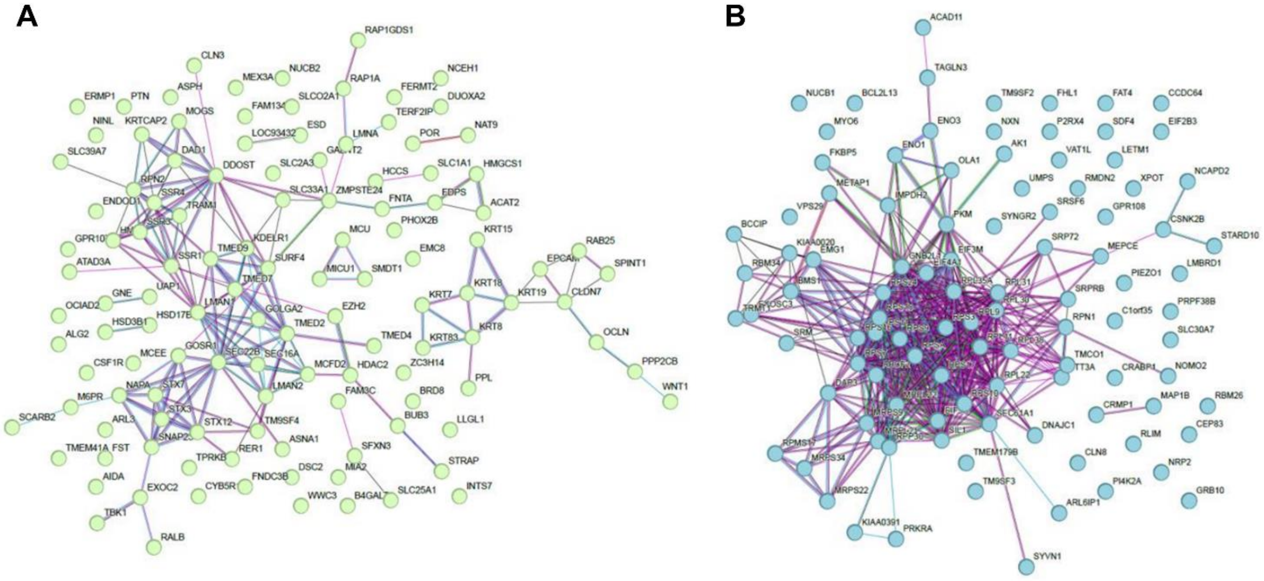

Figure S12. Network analysis of Day 24 WT and SMA iHeps. (A) STRING analysis of proteins in Cluster 2 (green) showed enrichment of golgi apparatus and endoplasmic reticulum related processes in the interaction network using the UniProt database. **(B**) STRING analysis of proteins in Cluster 3 (blue) showed enrichment of protein synthesis and metabolism related processes in the interaction network using the UniProt database.

Supplemental Figure 13

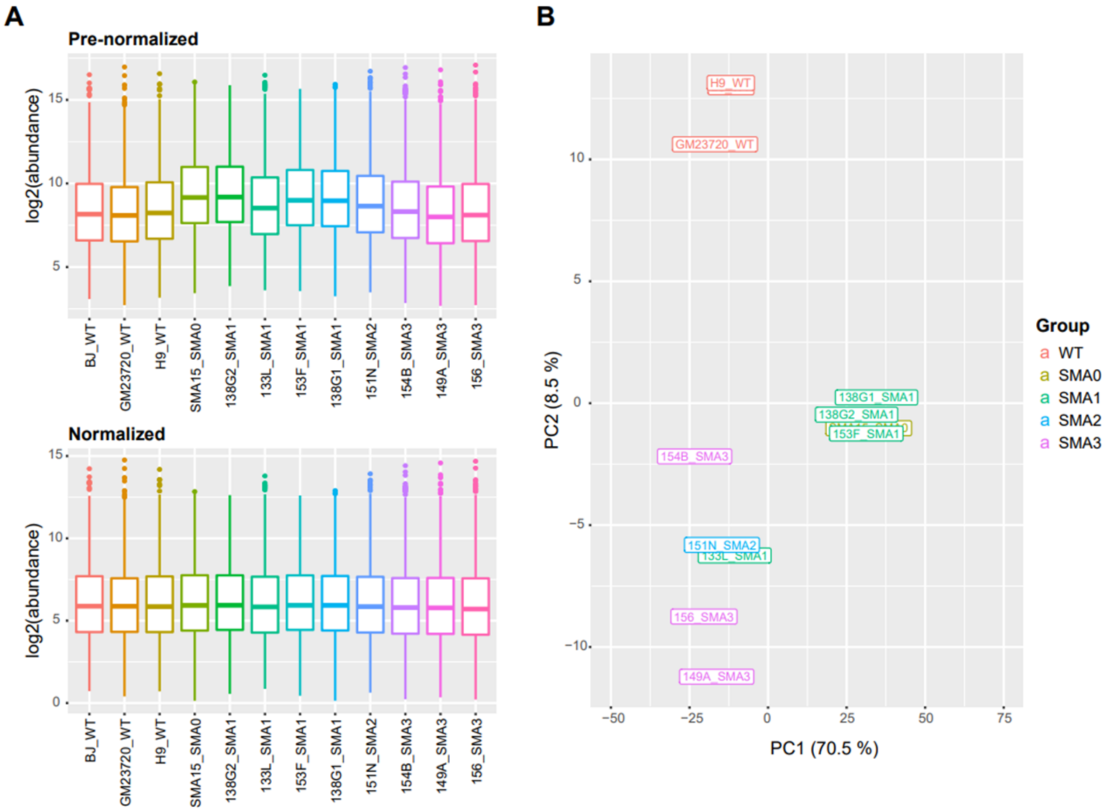

Figure S13. Normalization and principal component analysis (PCA) of Day 24 WT and SMA iHeps. (A) Total intensities for 4,058 proteins identified across all 12 iHeps samples were subjected to median normalization to eliminate potential differences across samples. Results are summarized using boxplots, with each bar representing the interquartile range, the horizontal line within each bar representing the median and the points outside of the whiskers as outliers. Box plots on the top panel show the distribution of the original protein intensities and the bottom panel show the distribution of the protein intensities after the median normalization. **(B)** Principal component analysis of all iHeps samples post-normalization. The first principal component explains 70.5% of the total data variability, separating the less severe cases (WT, SMA2, SMA3) from the more severe cases (SMA0, SMA1). The second principal component explains 8.5% of the total data variability, separating WT from the SMA samples.

Supplemental Figure 14

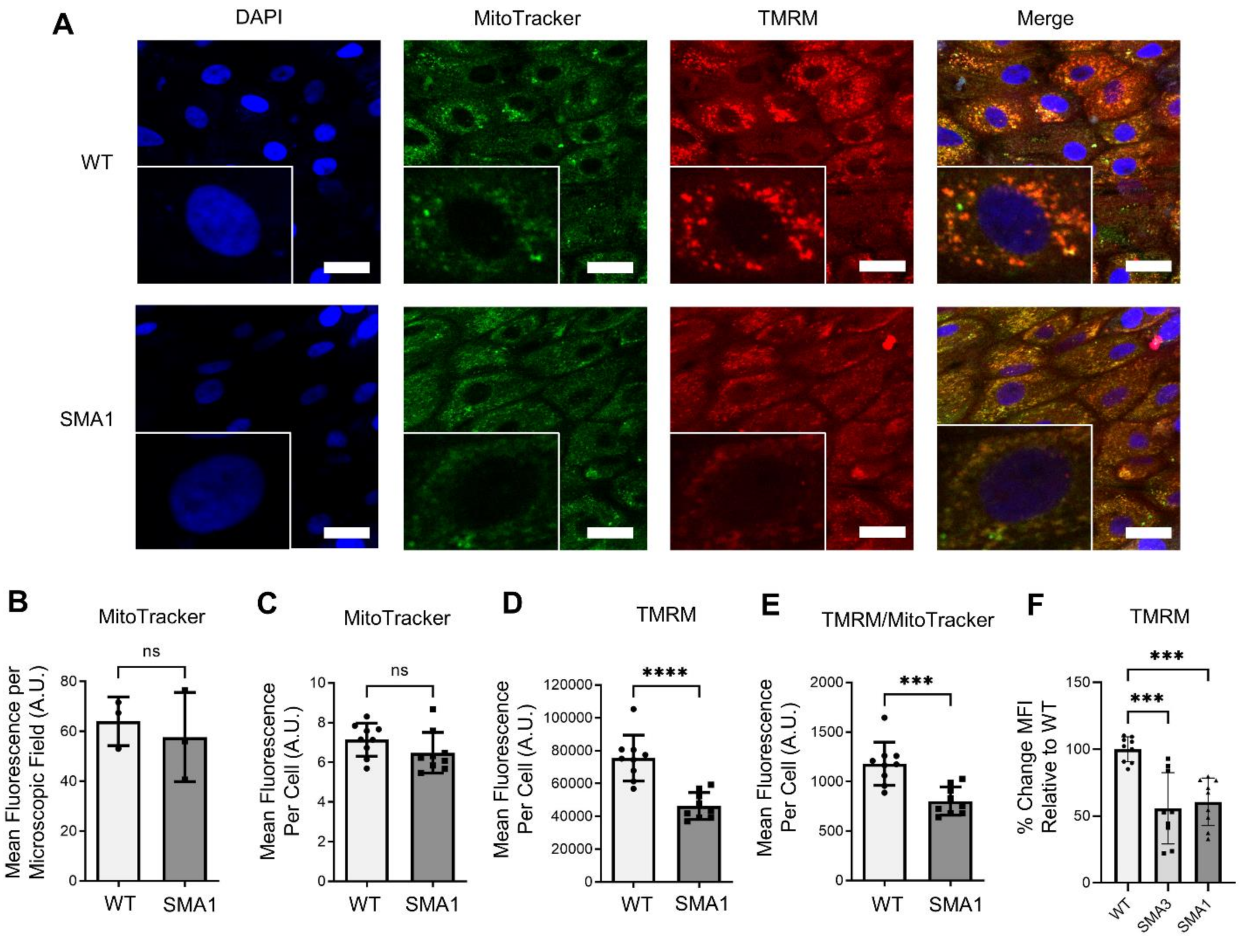

Figure S14. Mitochondrial membrane potential (MMP) reduction in day 24 SMA iHeps is intrinsic to mitochondria. (A) Confocal microscopy analysis of WT (BJ) and SMA1 (GM24468) iHeps. DAPI (blue) stains the nucleus. MitoTracker (green) stains mitochondria. Scale bar = 30µm. TMRM (red) staining positively correlates with MMP levels and co-localizes with MitoTracker staining. WT n=1 and SMA1 n=1. **(B - E)** ImageJ analysis of confocal staining showing that both TMRM only and TMRM normalized to MitoTracker staining showed reduction in the SMA1 iHep line. Data is from one independent experiment. Data was analyzed using unpaired two-tailed Student's t-test. **(B)** WT n=3, SMA1 n=3. **(C)** WT n=9, SMA1 n=9. **(D)** WT n=9, SMA1 n=9. **(E)** WT n=9, SMA1 n=9. **(F)** Mitochondrial membrane potential (MMP) quantification by TMRM, using flow cytometry. WT n=9, SMA1 n=9, SMA3 n=9, each with three biological replicates, and from three independent experiments. Data were analyzed using One-Way ANOVA test with Tukey's multiple comparison test. **(B – F)** No outliers were detected using the ROUT test with a maximum false discovery rate (FDR) of 1%. Data are presented as mean (±SD). *** p-value < 0.001; **** p-value < 0.0001.

Supplemental Figure 15

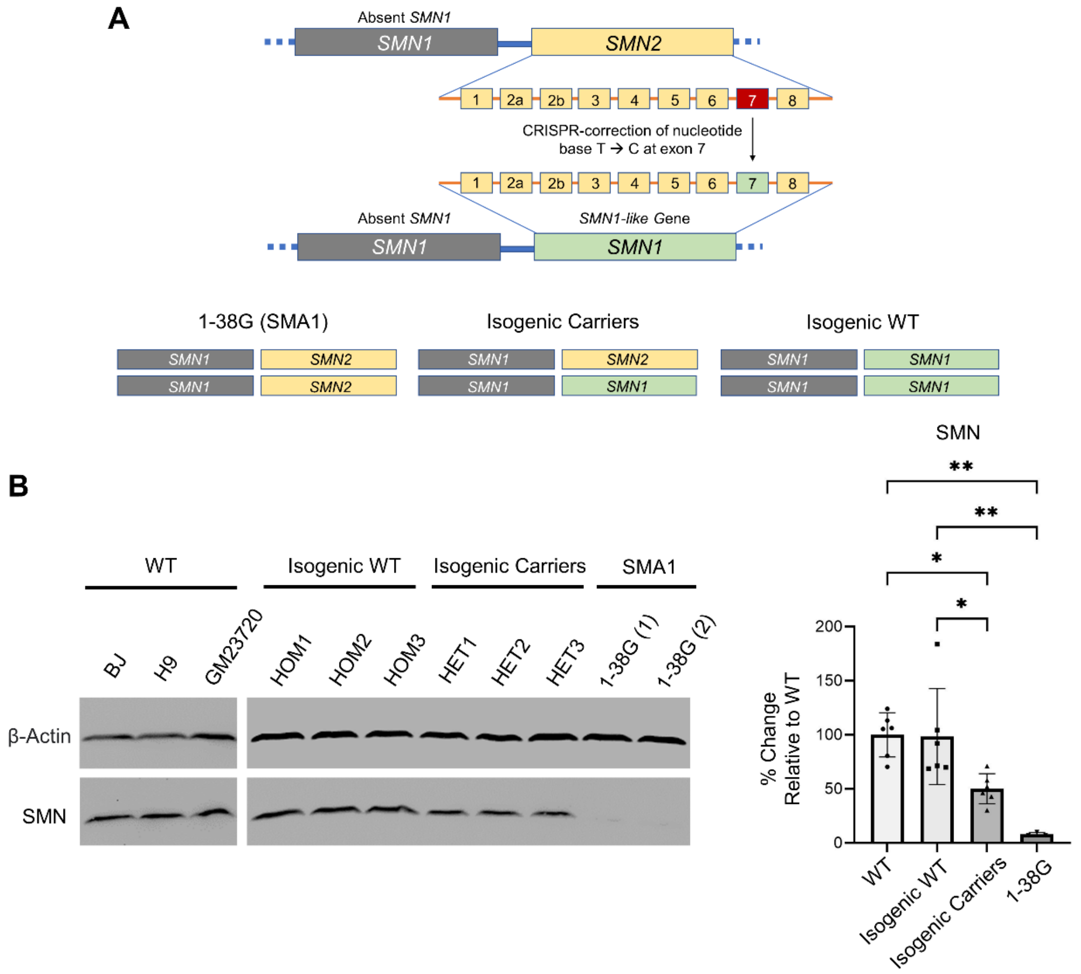

Figure S15. Rescue of SMN expression in Day 24 SMA Type I iHeps with CRISPR-editing. (A) CRISPR-editing of *SMN2* in the 1-38G (SMA1) cell line into *SMN1*-like genes through a T → C nucleotide change, to investigate SMN protein rescue on an isogenic background. **(B)** Western blot of SMN protein expression with beta-actin as the housekeeping protein for normalization, showing rescue of SMN protein expression, with the *SMN1*-like gene. Quantification of normalized SMN expression in the iHep lines was done using ImageJ analysis. Data was then presented as percentage levels relative to the mean of WTs. WT n=6, Isogenic WT n=6, Isogenic Carriers n=6, each with three biological replicates, and 1-38G n=3. Data is from two independent experiments. No outliers were detected using the ROUT test with a maximum false discovery rate (FDR) of 1%. Data are presented as mean (±SD). * p-value < 0.05; ** p-value < 0.01.

References

1. Stuhr, N. L., Nhan, J. D., Hammerquist, A. M., Van Camp, B., Reoyo, D. and Curran, S. P. (2022). Rapid Lipid Quantification in *Caenorhabditis elegans* by Oil Red O and Nile Red Staining. *Bio-protocol* 12(5): e4340. DOI: 10.21769/BioProtoc.4340.

Supplementary Table S4. Trends slopes for proteins with significant hits and their cluster assignments

Accession	Gene name	Protein description	Slope	Slope_Pval	logFC SMA	logFC SMA	logFC SMA	logFC SMA	NumSig	FDR	Cluster	logFC SMA/WT
P19883	FST	Follistatin	0.451353546	0.001600284	1.75401	1.342669	0.307093	0.308455	2	0.00956	Cluster 2	0.9280566
Q96BJ3	AIDA	Axin interactor, dorsalization-associated protein	0.387617565	2.25E-05	1.454758	1.198969	0.609857	0.353789	2	0.002708	Cluster 2	0.904343373
Q99453	PHOX2B	Paired mesoderm homeobox protein 2B	0.530508633	0.001004803	1.802044	1.520229	0.261724	-0.03673	1	0.007832	Cluster 2	0.886816462
P02771	AFP	Alpha-fetoprotein	-0.764456407	0.000716075	-2.62256	-2.51347	-1.06077	-0.76629	1	0.007112	Cluster 1	-1.740772871
P02671-2	FGA	Fibrinogen alpha chain	-0.588939426	0.001754457	-2.21572	-2.00051	-1.48337	-1.00216	1	0.009944	Cluster 1	-1.675440893
Q96PE1	ADGRA2	Adhesion G protein-coupled receptor A2	-0.832055309	0.001126074	-2.62195	-2.54353	-0.67194	-0.12079	1	0.008185	Cluster 1	-1.489550983
P04628	WNT1	Proto-oncogene Wnt-1	0.548864342	0.001505242	2.042324	1.311706	0.220903	-0.44589	2	0.009361	Cluster 2	0.782261959
P29762	CRABP1	Cellular retinoic acid-binding protein 1	0.862407274	0.001388493	3.009047	2.257033	0.186271	-0.48288	2	0.009113	Cluster 3	1.242368349
Q9NVV5	AIG1	Androgen-induced gene 1 protein	-0.600134244	0.001434356	-2.3459	-1.68301	-1.38308	-0.30494	2	0.009253	Cluster 1	-1.429234116
Q9ULE0	WWC3	Protein WWC3	0.239129242	3.35E-05	0.889447	0.87739	0.537525	0.534206	3	0.002821	Cluster 2	0.709642089
P36405	ARL3	ADP-ribosylation factor-like protein 3	0.394887585	0.000556123	1.580473	1.049083	0.094162	0.017231	2	0.00664	Cluster 2	0.68523718
Q01581	HMGCS1	Hydroxymethylglutaryl-CoA synthase, cytoplasmic	0.420201429	0.000449044	1.606741	1.08606	0.156593	-0.11772	2	0.006052	Cluster 2	0.682918545
O43684	BUB3	Mitotic checkpoint protein BUB3	0.348153215	0.000425463	1.38444	0.945978	0.211371	0.06725	2	0.006001	Cluster 2	0.652259911
Q9H0E9	BRD8	Bromodomain-containing protein 8	0.315056075	0.0002522	1.116407	0.847592	0.476048	-0.06272	2	0.005243	Cluster 2	0.594332599
Q9NR50	EIF2B3	Translation initiation factor eIF-2B subunit gamma	0.415188134	5.19E-05	1.773905	1.282953	0.784151	0.60629	2	0.003537	Cluster 3	1.1118247
Q9Y223	GNE	Bifunctional UDP-N-acetylglucosamine 2-epimerase/N-acetylmannosamine kinase	0.306286511	0.000214048	1.097241	0.850019	0.397529	0.001509	1	0.00516	Cluster 2	0.58657464
Q9Y2I6	NINL	Ninein-like protein	0.266427228	1.37E-05	0.958279	0.777736	0.481456	0.105408	2	0.002382	Cluster 2	0.580719767
P63173	RPL38	Ribosomal protein L38	0.391296321	8.49E-05	1.445238	1.307378	0.576116	0.54505	2	0.003998	Cluster 3	0.968445759
P02647	APOA1	Truncated apolipoprotein A-I	-0.472028288	6.73E-05	-1.66163	-1.68296	-1.23931	-0.8714	3	0.003542	Cluster 1	-1.363822853
Q9Y3F4	STRAP	Serine-threonine kinase receptor-associated protein	0.303973992	0.000722985	1.207927	0.81968	0.241006	0.050252	1	0.00712	Cluster 2	0.579716184
P82933	MRPS9	Mitochondrial ribosomal protein S9	0.304256469	2.29E-05	1.208725	1.041032	0.776225	0.599619	3	0.002708	Cluster 3	0.906400297
Q8N443	RIBC1	RIB43A domain with coiled-coils 1	-0.400881654	0.000580142	-1.39021	-1.57843	-1.35716	-1.08436	4	0.006707	Cluster 1	-1.352540001
A1L020	MEX3A	RNA-binding protein MEX3A	0.298208615	0.000230607	1.058315	0.856098	0.309595	0.046202	1	0.00516	Cluster 2	0.567552636
Q14692	BMS1	Ribosome biogenesis protein BMS1 homolog	0.427797341	0.000521646	1.549973	1.169794	0.445006	-0.03148	2	0.006568	Cluster 3	0.783323212
Q9NYB0	TERF2IP	Telomeric repeat-binding factor 2-interacting protein 1	0.344034187	0.001665637	1.248556	0.892219	0.207367	-0.14781	1	0.009759	Cluster 2	0.550082898
Q6Y1H2	HACD2	Very-long-chain (3R)-3-hydroxyacyl-CoA dehydratase 2	-0.491093769	0.000136298	-1.9267	-1.5352	-0.79668	-0.57041	2	0.004534	Cluster 1	-1.207247321
P42696	RBM34	RNA binding motif protein 34	0.367094273	0.000798523	1.3774	1.041543	0.357083	0.101461	2	0.007359	Cluster 3	0.719371878
Q14CX7	NAA25	N-alpha-acetyltransferase 25, NatB auxiliary subunit	-0.684107554	0.00039349	-2.2389	-1.99773	-0.58184	0.022116	2	0.0058	Cluster 1	-1.199087941
Q16222	UAP1	UDP-N-acetylgalactosamine pyrophosphorylase	0.33572011	0.000816973	1.160868	1.000277	-0.02899	0.060586	1	0.007396	Cluster 2	0.548185573
Q9P287	BCCIP	BRCA2 and CDKN1A-interacting protein	0.414186121	0.000822438	1.443585	1.141079	0.365039	-0.07596	2	0.007413	Cluster 3	0.718434517
Q15334	LLGL1	Lethal(2) giant larvae protein homolog 1	0.305561264	0.000367891	1.079272	0.895057	0.126268	0.060656	2	0.005768	Cluster 2	0.540313296
Q9Y3C4	TPRKB	EKC/KEOPS complex subunit TPRKB	0.257844967	0.000133578	0.994105	0.676296	0.481839	0.000723	1	0.004534	Cluster 2	0.538241013
O43772	SLC25A20	Mitochondrial carnitine/acylcarnitine carrier protein	-0.469751179	0.000394011	-1.82003	-1.56109	-0.5621	-0.70626	2	0.0058	Cluster 1	-1.162370934
P01023	A2M	Alpha-2-macroglobulin	-0.370939661	0.001217681	-1.42982	-1.37536	-0.69358	-0.89506	3	0.008503	Cluster 1	-1.098453666
P55289	CDH12	Cadherin-12	-0.804657852	0.001332762	-2.63982	-2.0983	-0.17347	0.635201	2	0.008955	Cluster 1	-1.069095498
Q9BU76	MMTAG2	Multiple myeloma tumor-associated protein 2	0.354429769	0.000514279	1.221761	1.050537	0.417414	0.097749	1	0.006535	Cluster 3	0.696865251
P07919	UQCRH	Cytochrome b-c1 complex subunit 6, mitochondrial	-0.362728716	0.000220738	-1.44477	-1.14523	-1.03327	-0.51755	2	0.00516	Cluster 1	-1.035204031
P14324	FDPS	Farnesyl pyrophosphate synthase	0.35556282	0.001024585	1.163532	0.987635	0.098787	-0.14687	1	0.007851	Cluster 2	0.525771408
Q9UDW1	UQCR10	Cytochrome b-c1 complex subunit 9	-0.327668253	0.000892602	-1.50388	-1.12493	-0.63817	-0.83436	3	0.007615	Cluster 1	-1.025335148
P55327	TPD52	Tumor protein D52	-0.323596414	0.000636652	-1.31301	-1.18712	-0.70446	-0.82932	3	0.006846	Cluster 1	-1.008478824
Q02338	BDH1	D-beta-hydroxybutyrate dehydrogenase, mitochondrial	-0.395062247	0.000131979	-1.52608	-1.28979	-0.62493	-0.55499	2	0.004534	Cluster 1	-0.998947915
O95831	AIFM1	Apoptosis-inducing factor 1, mitochondrial	-0.381091271	0.000342939	-1.403	-1.30775	-0.64774	-0.6125	2	0.005705	Cluster 1	-0.992746254
P10768	ESD	S-formylglutathione hydrolase	0.281042469	0.000262918	1.067076	0.736765	0.216621	-0.0508	1	0.005252	Cluster 2	0.492416524
P00568	AK1	Adenylate kinase isoenzyme 1	0.21554327	0.000525864	0.819006	0.804802	0.601074	0.542757	2	0.006581	Cluster 3	0.69190989
Q96KP1	EXOC2	Exocyst complex component 2	0.242438436	0.000182839	0.915812	0.63622	0.432041	-0.01981	1	0.004833	Cluster 2	0.49106604
Q8WTV0	SCARB1	Scavenger receptor class B member 1	-0.33438556	0.000541944	-1.34431	-1.12886	-0.80804	-0.63597	2	0.006627	Cluster 1	-0.979293359
Q9NVH2	INTS7	Integrator complex subunit 7	0.274426535	0.000965832	1.079156	0.701302	0.194398	-0.05457	1	0.007788	Cluster 2	0.480071475
Q92769	HDAC2	Histone deacetylase 2	0.303543774	0.000610052	1.078581	0.81458	0.112125	-0.10049	2	0.006765	Cluster 2	0.476197825
Q8NBQ5	HSD17B11	Estradiol 17-beta-dehydrogenase 11	-0.533737591	0.000254064	-1.91953	-1.56435	-0.28004	-0.14891	2	0.005243	Cluster 1	-0.97820537

Q9Y2R5	MRPS17	Mitochondrial ribosomal protein S17	0.236532496	1.46E-05	0.980892	0.783671	0.551992	0.444671	2	0.002389	Cluster 3	0.690306431
O43681	ASNA1	ATPase GET3	0.246293725	0.000288871	0.948327	0.677762	0.180377	0.038935	2	0.005524	Cluster 2	0.461350316
Q96ND0	FAM210A	Protein FAM210A	-0.299695885	0.001458486	-1.22372	-1.13149	-0.69124	-0.8513	3	0.009253	Cluster 1	-0.974438769
Q00325-2	SLC25A3	Phosphate carrier protein, mitochondrial	-0.336522715	0.000184111	-1.29541	-1.13943	-0.79955	-0.58848	2	0.004833	Cluster 1	-0.95571788
O95678	KRT75	Keratin, type II cytoskeletal 75	-0.526913191	0.000965205	-1.93185	-1.4726	-0.3708	-0.0349	1	0.007788	Cluster 1	-0.95253689
O75569	PRKRA	Interferon-inducible double-stranded RNA-dependent protein kinase activator A	0.339721987	0.000308274	1.193791	1.034471	0.358349	0.17296	1	0.005657	Cluster 3	0.689892932
P52306	RAP1GDS1	Rap1 GTPase-GDP dissociation stimulator 1	0.2988392	0.000920444	1.136225	0.764954	0.046753	-0.11387	2	0.007684	Cluster 2	0.458515815
Q6PJT7	ZC3H14	Zinc finger CCCH domain-containing protein 14	0.218299904	0.000979454	0.645215	0.722208	0.217735	0.11557	1	0.007807	Cluster 2	0.425181843
P08708	RPS17	Ribosomal protein S17	0.312730411	0.000142765	1.18974	0.92825	0.419236	0.206543	2	0.004614	Cluster 3	0.685942216
P21796	VDAC1	Voltage-dependent anion-selective channel protein 1	-0.445156399	0.000433201	-1.54054	-1.43166	-0.42484	-0.36712	1	0.006018	Cluster 1	-0.941040123
Q96EC8	YIPF6	Protein YIPF6	-0.361603968	9.10E-05	-1.37179	-1.18845	-0.61349	-0.5051	2	0.004014	Cluster 1	-0.919705919
P62714	PPP2CB	Serine/threonine-protein phosphatase 2A catalytic subunit beta isoform	0.260755833	0.000454948	0.959821	0.689268	0.075405	-0.07868	2	0.006096	Cluster 2	0.41145381
Q96AC1	FERMT2	Fermitin family homolog 2	0.203003701	0.000589715	0.663969	0.648715	0.141021	0.113746	1	0.006731	Cluster 2	0.39186297
P46821	MAP1B	Microtubule-associated protein 1B	0.395231198	0.001456548	1.361111	1.174242	0.104319	0.07373	1	0.009253	Cluster 3	0.678350478
Q15910	EZH2	Histone-lysine N-methyltransferase EZH2	0.22687051	0.001304367	0.804435	0.660917	-0.06117	0.022846	1	0.008881	Cluster 2	0.356757927
P08574	CYC1	Cytochrome c1, heme protein, mitochondrial	-0.311461019	0.000267536	-1.18427	-1.0924	-0.67738	-0.60708	2	0.005252	Cluster 1	-0.890282654
Q8NFQ8	TOR1AIP2	Torsin-1A-interacting protein 2	-0.465875207	0.000410587	-1.68293	-1.32798	-0.46506	-0.07867	2	0.005895	Cluster 1	-0.888659961
Q14194	CRMP1	Dihydropyrimidinase-related protein 1	0.445846215	0.001016609	1.63628	1.169559	0.056394	-0.16736	2	0.00785	Cluster 3	0.673718928
P46783	RPS10	40S ribosomal protein S10	0.340422374	0.000646106	1.292885	0.954008	0.265974	0.076056	2	0.006846	Cluster 3	0.647230519
Q9BWD1	ACAT2	Acetyl-CoA acetyltransferase, cytosolic	0.271526987	0.001163177	0.908502	0.682818	-0.03471	-0.26308	1	0.008273	Cluster 2	0.323382137
Q9BTE0	NAT9	N-acetyltransferase 9	0.184230115	3.91E-05	0.559643	0.53011	0.229698	-0.05864	1	0.00286	Cluster 2	0.315202224
P53582	METAP1	Methionine aminopeptidase 1	0.310317949	6.82E-05	1.15666	0.866544	0.491147	0.068186	2	0.003542	Cluster 3	0.645634328
P49354	FNTA	Protein farnesyltransferase/geranylgeranyltransferase type-1 subunit alpha	0.205500965	0.001027936	0.702457	0.532916	0.118749	-0.13227	2	0.007858	Cluster 2	0.305464032
Q92959	SLCO2A1	Solute carrier organic anion transporter family member 2A1	-0.282670612	0.001500684	-0.97268	-0.71364	0.10826	0.248778	2	0.009361	Cluster 2	-0.332320721
Q96PE7	MCEE	Methylmalonyl-CoA epimerase, mitochondrial	-0.260197407	0.001301462	-0.94285	-0.63999	-0.15303	0.191015	2	0.008881	Cluster 2	-0.386214319
Q13247	SRSF6	Serine/arginine-rich splicing factor 6	0.450991665	0.001777651	1.478833	1.24269	-0.04715	-0.22468	1	0.009992	Cluster 3	0.612423524
Q6P2E9	EDC4	Enhancer of mRNA-decapping protein 4	-0.419416417	0.000383079	-1.51653	-1.29225	-0.41557	-0.28681	1	0.0058	Cluster 1	-0.8777886
O75396	SEC22B	Vesicle-trafficking protein SEC22b	-0.231270306	0.00153884	-0.89432	-0.62284	-0.11148	-0.00379	1	0.009427	Cluster 2	-0.408109128
P18859	ATP5PF	ATP synthase-coupling factor 6, mitochondrial	-0.329079255	2.32E-05	-1.24614	-1.07057	-0.71203	-0.45006	3	0.002708	Cluster 1	-0.869696957
Q13451	FKBP5	Peptidyl-prolyl cis-trans isomerase FKBP5	0.366654861	0.001645831	1.259695	1.023588	0.21675	-0.06739	1	0.009704	Cluster 3	0.60816026
O00161	SNAP23	Synaptosomal-associated protein 23	-0.252389304	0.000444556	-0.87231	-0.67659	-0.20957	0.096718	2	0.006052	Cluster 2	-0.41543741
Q8N6L1	KRTCAP2	Keratinocyte-associated protein 2	-0.24184024	0.00130218	-0.80662	-0.65415	-0.34351	0.093075	1	0.008881	Cluster 2	-0.427800833
O43402	EMC8	ER membrane protein complex subunit 8	-0.129849168	0.00124985	-0.43123	-0.51782	-0.42153	-0.34472	1	0.008654	Cluster 2	-0.42882586
P19440	GGT1	Glutathione hydrolase 1 heavy chain	-0.450325695	0.000230764	-1.658	-1.28014	-0.39317	-0.09321	2	0.00516	Cluster 1	-0.85612734
Q969L2	MAL2	Protein MAL2	-0.308626734	0.000562465	-1.0152	-1.09031	-0.8134	-0.47652	1	0.006666	Cluster 1	-0.848858183
P22090	RPS4Y1	Ribosomal protein S4 Y-linked 1	0.276606333	0.000160149	0.878752	0.790453	0.458169	-0.04911	1	0.004763	Protein not	in STRING database
P30049	ATP5F1D	ATP synthase subunit delta, mitochondrial	-0.303105951	3.89E-05	-1.17575	-0.99165	-0.71133	-0.46107	3	0.00286	Cluster 1	-0.834949901
Q9HCJ6	VAT1L	Synaptic vesicle membrane protein VAT-1 homolog-like	0.347450826	0.000173442	1.294437	0.959954	0.161924	0.009758	2	0.004803	Cluster 3	0.606518377
Q86VR2	RETREG3	Reticulophagy regulator 3	-0.180732144	3.84E-05	-0.66728	-0.55545	-0.4301	-0.16216	1	0.00286	Cluster 2	-0.453746629
Q9BWQ6	YIPF2	Protein YIPF2	-0.409416044	6.34E-05	-1.40262	-1.27324	-0.39388	-0.22713	2	0.003542	Cluster 1	-0.82421666
P05141	SLC25A5	ADP/ATP translocase 2, N-terminally processed	-0.280880991	0.00017026	-1.0818	-0.99684	-0.62702	-0.58906	2	0.004803	Cluster 1	-0.823679038
Q6NUM9	RETSAT	All-trans-retinol 13,14-reductase	-0.377250886	0.00032534	-1.47235	-1.17936	-0.2359	-0.38877	2	0.005705	Cluster 1	-0.819092934
P63244	RACK1	Receptor of activated protein C kinase 1, N-terminally processed	0.282077721	0.00033635	1.170309	0.754042	0.365869	0.097175	1	0.005705	Cluster 3	0.59684903
P12235	SLC25A4	ADP/ATP translocase 1	-0.267164125	0.000102693	-1.03072	-0.97947	-0.58122	-0.62994	3	0.004202	Cluster 1	-0.805338493
P82930	MRPS34	28S ribosomal protein S34, mitochondrial	0.207091748	2.11E-05	0.682921	0.774382	0.514397	0.413019	1	0.002708	Cluster 3	0.596179939
P35268	RPL22	Ribosomal protein L22	0.248133531	7.40E-05	0.948953	0.747208	0.42477	0.202847	2	0.003692	Cluster 3	0.580944417
Q9BPX6	MICU1	Calcium uptake protein 1, mitochondrial	-0.169146384	0.000720631	-0.74862	-0.53082	-0.29523	-0.28836	1	0.00712	Cluster 2	-0.46575842
Q5VW38	GPR107	Protein GPR107	-0.16379296	3.32E-05	-0.66515	-0.54746	-0.38388	-0.30465	2	0.002821	Cluster 2	-0.475284823
Q13488	TCIRG1	V-type proton ATPase 116 kDa subunit a isoform 3	-0.313413889	0.000986426	-1.3126	-1.02455	-0.35282	-0.52925	1	0.007807	Cluster 1	-0.804803087
Q8NI22	MCFD2	Multiple coagulation factor deficiency protein 2	-0.256887266	5.65E-05	-0.91417	-0.72594	-0.30747	-0.0212	2	0.003542	Cluster 2	-0.492194906
P62888	RPL30	Ribosomal protein L30	0.225299403	2.51E-05	0.839189	0.718159	0.491964	0.2618	1	0.002741	Cluster 3	0.577778201

P21397	MAOA	Amine oxidase [flavin-containing] A	-0.287132175	0.00096686	-1.14427	-1.00675	-0.45826	-0.593	1	0.007788	Cluster 1	-0.800572723
P78385	KRT83	Keratin, type II cuticular Hb3	-0.269420289	5.32E-05	-0.90116	-0.78292	-0.28075	-0.00791	2	0.003537	Cluster 2	-0.493186752
P80303	NUCB2	Nucleobindin-2	-0.248651112	0.000135479	-0.97203	-0.6608	-0.32943	-0.0182	2	0.004534	Cluster 2	-0.495114525
P62280	RPS11	Ribosomal protein S11	0.29366555	0.000327552	1.029185	0.854632	0.309869	0.058771	1	0.005705	Cluster 3	0.563114189
P62081	RPS7	40S ribosomal protein S7	0.245548014	7.15E-05	0.965732	0.720803	0.381805	0.181742	2	0.003639	Cluster 3	0.562520451
O15091	PRORP	Mitochondrial ribonuclease P catalytic subunit	0.213180376	1.35E-05	0.855158	0.665148	0.433334	0.273295	2	0.002382	Cluster 3	0.556733728
Q9UG63	ABCF2	ATP binding cassette subfamily F member 2	0.26491254	3.25E-05	1.008205	0.737575	0.402823	0.072328	2	0.002821	Cluster 3	0.555232469
Q07065	CKAP4	Cytoskeleton-associated protein 4	-0.333160602	5.73E-05	-1.26264	-0.98225	-0.71727	-0.23054	2	0.003542	Cluster 1	-0.798177788
Q92520	FAM3C	Protein FAM3C	-0.227076343	1.07E-05	-0.7953	-0.70254	-0.33483	-0.14817	2	0.002382	Cluster 2	-0.495211159
Q7Z2K6	ERMP1	Endoplasmic reticulum metallopeptidase 1	-0.220852017	0.000889181	-0.98646	-0.61735	-0.24898	-0.20202	1	0.007615	Cluster 2	-0.513702388
Q9Y3D6	FIS1	Mitochondrial fission 1 protein	-0.271083505	0.000169954	-0.96827	-0.93696	-0.79204	-0.45769	4	0.004803	Cluster 1	-0.788738436
Q6P1A2	LPCAT3	Lysophospholipid acyltransferase 5	-0.302202771	0.000376495	-1.19677	-0.96784	-0.46225	-0.41044	2	0.005794	Cluster 1	-0.759323041
P46781	RPS9	Ribosomal protein S9	0.355042262	0.000938475	1.180144	0.959009	0.206932	-0.1767	2	0.007715	Cluster 3	0.542345426
P62834	RAP1A	Ras-related protein Rap-1A	-0.223914584	0.000440433	-0.95621	-0.67222	-0.17604	-0.25532	1	0.006052	Cluster 2	-0.514949678
Q08379	GOLGA2	Golgin subfamily A member 2	-0.194182851	1.37E-05	-0.83198	-0.58957	-0.37866	-0.26396	2	0.002382	Cluster 2	-0.516043135
Q96PC5	MIA2	Melanoma inhibitory activity protein 2	-0.207475082	0.000124982	-0.8755	-0.65178	-0.25865	-0.30094	2	0.004526	Cluster 2	-0.521714927
P23396	RPS3	40S ribosomal protein S3	0.316944624	0.000655095	1.217135	0.814393	0.21313	-0.08365	2	0.006873	Cluster 3	0.540251603
P20645	M6PR	Cation-dependent mannose-6-phosphate receptor	-0.186086341	0.000632448	-0.73273	-0.67576	-0.25782	-0.42288	1	0.006846	Cluster 2	-0.522297582
Q9H553	ALG2	Alpha-1,3/1,6-mannosyltransferase ALG2	-0.173876155	0.000554329	-0.66944	-0.61972	-0.43002	-0.37493	1	0.00664	Cluster 2	-0.523526203
Q8TBQ9	TMEM167	Protein kish-A	-0.377862991	0.000397511	-1.34219	-1.20172	-0.19466	-0.29766	2	0.005809	Cluster 1	-0.759059122
Q9GZM5	YIPF3	Protein YIPF3, 36 kDa form III	-0.39587572	0.000615552	-1.45499	-1.22108	-0.04477	-0.25872	2	0.006789	Cluster 1	-0.744889241
Q8WUK0	PTPMT1	Phosphatidylglycerophosphatase and protein-tyrosine phosphatase 1	-0.291930429	0.000115747	-1.14585	-0.97369	-0.38011	-0.46497	2	0.004443	Cluster 1	-0.741156927
O15027	SEC16A	Protein transport protein Sec16A	-0.224586201	0.000787235	-0.63538	-0.73989	-0.59543	-0.12483	1	0.007321	Cluster 2	-0.523883334
Q15165-1	PON2	Serum paraoxonase/arylesterase 2	-0.350388923	3.23E-05	-1.28562	-1.08391	-0.28145	-0.26063	2	0.002821	Cluster 1	-0.72790449
P12268	IMPDH2	Inosine-5'-monophosphate dehydrogenase 2	0.325722955	0.001294165	1.239785	0.883001	-0.00925	-0.02006	2	0.008856	Cluster 3	0.523369898
Q92903	CDS1	Phosphatidate cytidylyltransferase 1	-0.315264614	0.000531113	-1.07876	-1.0702	-0.36295	-0.37969	1	0.006594	Cluster 1	-0.722898746
Q5VTL8	PRPF38B	Pre-mRNA-splicing factor 38B	0.251858563	0.000377486	0.927598	0.701273	0.413282	0.041302	1	0.005794	Cluster 3	0.520863592
Q10471	GALNT2	Polypeptide N-acetylgalactosaminyltransferase 2 soluble form	-0.21064934	6.79E-06	-0.8026	-0.68089	-0.35078	-0.27158	2	0.002382	Cluster 2	-0.526462329
P18077	RPL35A	60S ribosomal protein L35a	0.233870908	0.000209823	0.894168	0.711417	0.267679	0.192112	2	0.00516	Cluster 3	0.51634387
P60842	EIF4A1	Eukaryotic initiation factor 4A-I	0.291398267	0.0009812	1.145267	0.755342	0.196912	-0.03592	1	0.007807	Cluster 3	0.51539995
P62269	RPS18	40S ribosomal protein S18	0.33208665	0.001722404	1.190164	0.885012	0.096939	-0.11634	2	0.009885	Cluster 3	0.513944124
P62249	RPS16	Ribosomal protein S16	0.328332785	0.001667007	1.153578	0.862839	0.171057	-0.15684	2	0.009759	Cluster 3	0.507658432
Q13724	MOGS	Mannosyl-oligosaccharide glucosidase	-0.223295733	8.56E-06	-0.80891	-0.71677	-0.37028	-0.23473	2	0.002382	Cluster 2	-0.532671906
O95249	GOSR1	Golgi SNAP receptor complex member 1	-0.165228336	0.000467254	-0.63401	-0.60374	-0.5797	-0.40613	1	0.006128	Cluster 2	-0.555895862
P63241	EIF5A	Eukaryotic translation initiation factor 5A-1	0.28247383	0.000586359	1.080925	0.763463	0.16537	0.002532	1	0.006731	Cluster 3	0.50307252
P51398	DAP3	28S ribosomal protein S29, mitochondrial	0.171337416	0.000120908	0.67621	0.563801	0.469094	0.286887	1	0.004466	Cluster 3	0.498997962
Q5T8P6	RBM26	RNA binding motif protein 26	0.278246205	0.000752296	1.068102	0.773751	0.097877	0.046878	1	0.00712	Cluster 3	0.496651985
P08865	RPSA	40S ribosomal protein SA	0.260858677	0.001098168	1.084114	0.676925	0.194728	0.030425	1	0.008058	Cluster 3	0.496548077
P14618	PKM	Pyruvate kinase PKM	0.299331946	0.000599784	1.119937	0.786705	0.092133	-0.08165	1	0.006731	Cluster 3	0.479280675
P11172	UMPS	Orotidine 5'-phosphate decarboxylase	0.343834815	0.001709158	1.312228	0.848242	-0.04549	-0.20799	2	0.009864	Cluster 3	0.476747426
Q7L2J0	MEPCE	7SK snRNA methylphosphate capping enzyme	0.315326693	0.001156541	1.071367	0.855075	0.110535	-0.13441	1	0.00827	Cluster 3	0.475640849
P78346	RPP30	Ribonuclease P protein subunit p30	0.238732991	0.00030547	0.917691	0.689108	0.184699	0.108761	2	0.005641	Cluster 3	0.475064572
Q9NTK5	OLA1	Obg-like ATPase 1	0.261537056	0.000367564	1.008639	0.703548	0.163288	0.003912	1	0.005768	Cluster 3	0.469846629
Q9Y3B3	TMED7	Transmembrane emp24 domain-containing protein 7	-0.231842318	1.94E-05	-0.86441	-0.68135	-0.54381	-0.14629	2	0.002708	Cluster 2	-0.558965952
O15400	STX7	Syntaxin-7	-0.227960356	0.000292701	-0.92798	-0.72715	-0.26955	-0.31954	2	0.005571	Cluster 2	-0.561054537
O60462	NRP2	Neuropilin-2	0.327190165	0.001543453	1.211793	0.855124	-0.09061	-0.13355	1	0.009441	Cluster 3	0.460690409
P62899	RPL31	Ribosomal protein L31	0.19083733	0.00173535	0.510706	0.727819	0.322636	0.276761	1	0.009932	Cluster 3	0.459480727
Q13322	GRB10	Growth factor receptor-bound protein 10	0.177921888	7.84E-05	0.768572	0.4659	0.48602	0.098457	1	0.003818	Cluster 3	0.454737345
O15126	SCAMP1	Secretory carrier-associated membrane protein 1	-0.299556105	1.17E-06	-1.23705	-0.91702	-0.34435	-0.35087	2	0.002382	Cluster 1	-0.712324623
Q5VTU8	ATP5F1EP	ATP synthase subunit epsilon-like protein, mitochondrial	-0.245268268	0.000230242	-0.93318	-0.83275	-0.63617	-0.42899	2	0.00516	Cluster 1	-0.707771624
P39656	DDOST	Dolichyl-diphosphooligosaccharide--protein glycosyltransferase 48 kDa subunit	-0.243769051	2.98E-05	-0.87976	-0.76002	-0.40274	-0.20285	1	0.002821	Cluster 2	-0.561342185
P61421	ATP6V0D1	V-type proton ATPase subunit d 1	-0.290108283	0.000525046	-1.15297	-0.94407	-0.30906	-0.4164	1	0.006581	Cluster 1	-0.705626066
Q15629	TRAM1	Translocating chain-associated membrane protein 1	-0.298894265	0.001288818	-1.11686	-0.88836	-0.1017	-0.14656	1	0.008834	Cluster 2	-0.563370734
Q13642	FHL1	Four and a half LIM domains protein 1	0.172410694	0.000331724	0.65605	0.587403	0.286226	0.288241	1	0.005705	Cluster 3	0.454479982
P12236	SLC25A6	ADP/ATP translocase 3, N-terminally processed	-0.289213698	3.40E-06	-1.05937	-0.94982	-0.44835	-0.35989	2	0.002382	Cluster 1	-0.70435496
P06576	ATP5F1B	ATP synthase subunit beta, mitochondrial	-0.216120903	0.000449636	-0.82488	-0.76274	-0.68846	-0.45916	2	0.006052	Cluster 1	-0.683810886
P53701	HCCS	Cytochrome c-type heme lyase	-0.220996811	0.000148151	-0.81611	-0.7804	-0.3688	-0.40589	2	0.004639	Cluster 2	-0.592798987
Q0VDD5	MIR22HG	Putative uncharacterized protein encoded by MIR22HG	-0.167184083	0.000729005	-0.8332	-0.55682	-0.48517	-0.47123	1	0.00712	Protein not in STRING database	

Q53EP0	FNDC3B	Fibronectin type III domain-containing protein 3B	-0.308817154	0.000557221	-1.07934	-0.90521	-0.35805	-0.07684	1	0.00664	Cluster 2	-0.60485903
Q02487	DSC2	Desmocollin-2	-0.253607908	0.000414866	-0.98603	-0.80514	-0.35569	-0.30684	1	0.005915	Cluster 2	-0.6134218
Q9BVK6	TMED9	Transmembrane emp24 domain-containing protein 9	-0.304221574	0.000280328	-1.1155	-0.87547	-0.37208	-0.09391	1	0.005385	Cluster 2	-0.614239776
P82650	MRPS22	Mitochondrial ribosomal protein S22	0.168830233	2.48E-06	0.705703	0.537449	0.297795	0.263579	2	0.002382	Cluster 3	0.451131581
O75844	ZMPSTE24	CAAX prenyl protease 1 homolog	-0.220604237	7.11E-06	-0.86559	-0.72364	-0.5695	-0.35542	2	0.002382	Cluster 2	-0.628534768
O15260	SURF4	Surfeit locus protein 4	-0.301236854	0.000127436	-1.10258	-0.89969	-0.37298	-0.16443	1	0.004534	Cluster 2	-0.634918399
P62913	RPL11	60S ribosomal protein L11	0.206721569	0.000597089	0.89944	0.544738	0.252338	0.09376	1	0.006731	Cluster 3	0.44756908
Q9NQT5	EXOSC3	Exosome complex component RRP40	0.251790041	0.000191471	0.926531	0.689129	0.161717	-0.01403	2	0.004928	Cluster 3	0.440837944
Q9Y6C9	MTCH2	Mitochondrial carrier homolog 2	-0.264950729	0.000170036	-0.98599	-0.897	-0.43451	-0.40753	2	0.004803	Cluster 1	-0.681256731
P09327	VIL1	Villin-1	-0.427293796	0.001313757	-1.51971	-1.23028	0.105499	-0.01648	2	0.008915	Cluster 1	-0.665244129
P67870	CSNK2B	Casein kinase II subunit beta	0.244880246	0.000254019	0.970927	0.654771	0.118507	0.017548	1	0.005243	Cluster 3	0.44043833
Q92544	TM9SF4	Transmembrane 9 superfamily member 4	-0.222592108	0.000379911	-0.83385	-0.79039	-0.49359	-0.44393	1	0.0058	Cluster 2	-0.640440563
P24539	ATP5PB	ATP synthase F(0) complex subunit B1, mitochondrial	-0.19005362	0.000704853	-0.75743	-0.71145	-0.60947	-0.52674	3	0.007091	Cluster 1	-0.65127508
P49257	LMAN1	Protein ERGIC-53	-0.226491872	5.19E-05	-0.87572	-0.73311	-0.62674	-0.33473	1	0.003537	Cluster 2	-0.642574411
Q15397	PUM3	Pumilio homolog 3	0.222922604	0.000484296	0.836322	0.617127	0.253261	0.031399	2	0.006252	Cluster 3	0.434527141
P27338	MAOB	Amine oxidase [flavin-containing] B	-0.350370864	0.000636382	-1.37436	-1.07135	0.131201	-0.27579	2	0.006846	Cluster 1	-0.647573736
Q92979	EMG1	Ribosomal RNA small subunit methyltransferase NEP1	0.270262774	0.000773008	1.025713	0.701985	0.063367	-0.07988	2	0.007238	Cluster 3	0.427797422
P56385	ATP5ME	ATP synthase subunit e, mitochondrial, N-terminally processed	-0.214621985	0.000219659	-0.79855	-0.75178	-0.6019	-0.41369	2	0.00516	Cluster 1	-0.641481943
Q9NXH9	TRMT1	tRNA (guanine(26)-N(2))-dimethyltransferase	0.263172654	0.000678651	0.698904	0.813859	0.260114	-0.0661	1	0.006909	Cluster 3	0.42669316
O43592	XPOT	Exportin-T	0.255335769	0.00135144	0.955172	0.672906	0.112898	-0.06192	1	0.009023	Cluster 3	0.419762926
P25705	ATP5F1A	ATP synthase subunit alpha, mitochondrial	-0.185532049	0.001485726	-0.72345	-0.67318	-0.68431	-0.46074	1	0.009361	Cluster 1	-0.63541798
Q99735	MGST2	Microsomal glutathione S-transferase 2	-0.320311889	0.000885177	-1.21724	-0.97438	-0.11971	-0.22831	2	0.007615	Cluster 1	-0.634909802
Q96AG4	LRRC59	Leucine-rich repeat-containing protein 59, N-terminally processed	-0.274198464	8.17E-06	-1.00638	-0.85768	-0.39573	-0.24479	2	0.002382	Cluster 1	-0.626144063
P04844	RPN2	Dolichyl-diphosphooligosaccharide--protein glycosyltransferase subunit 2	-0.258763177	1.78E-05	-0.92446	-0.83985	-0.53411	-0.29125	1	0.002708	Cluster 2	-0.647418442
O43278	SPINT1	Kunitz-type protease inhibitor 1	-0.316818312	0.000230672	-1.19817	-0.9665	-0.21914	-0.23732	2	0.00516	Cluster 2	-0.655283467
P62847	RPS24	40S ribosomal protein S24	0.255306932	0.000600611	0.897139	0.688437	0.157431	-0.08021	2	0.006731	Cluster 3	0.415699471
Q9BWM7	SFXN3	Sideroflexin-3	-0.275892896	3.38E-05	-1.08058	-0.90136	-0.28458	-0.38695	2	0.002821	Cluster 2	-0.663369537
Q8NBJ4	GOLM1	Golgi membrane protein 1	-0.200152085	0.000328453	-0.8211	-0.70587	-0.47164	-0.46283	2	0.005705	Cluster 1	-0.615359491
P23142	FBLN1	Fibulin-1	-0.320892989	8.98E-05	-0.84362	-1.04352	-0.51111	-0.04606	1	0.004014	Cluster 1	-0.611075697
P11169	SLC2A3	Solute carrier family 2, facilitated glucose transporter member 3	-0.321522515	0.001394642	-1.09674	-1.04643	-0.24092	-0.26941	1	0.009131	Cluster 2	-0.663375462
Q15363	TMED2	Transmembrane emp24 domain-containing protein 2	-0.285793686	9.41E-05	-1.06268	-0.86247	-0.53394	-0.21242	1	0.004035	Cluster 2	-0.66787727
Q14573	ITPR3	Inositol 1,4,5-trisphosphate receptor type 3	-0.273344202	0.001609825	-1.08647	-0.91256	-0.01052	-0.41249	2	0.009589	Cluster 1	-0.605510587
Q9Y277	VDAC3	Voltage-dependent anion-selective channel protein 3	-0.219676623	0.001223431	-0.63705	-0.83876	-0.5135	-0.38565	1	0.008529	Cluster 1	-0.59373754
Q12907	LMAN2	Vesicular integral-membrane protein VIP36	-0.317214814	6.43E-05	-1.19161	-0.93865	-0.38533	-0.18342	2	0.003542	Cluster 2	-0.674754502
Q99715	COL12A1	Collagen alpha-1(XII) chain	-0.302099317	0.000198527	-0.99349	-0.92701	-0.28324	-0.09754	2	0.005077	Cluster 1	-0.575318284
Q9H4I9	SMDT1	Essential MCU regulator, mitochondrial	-0.197389043	0.001164581	-1.15738	-0.55378	-0.53078	-0.49502	2	0.008273	Cluster 2	-0.684239489
Q7Z7H5	TMED4	Transmembrane emp24 domain-containing protein 4	-0.212928585	0.000338097	-0.76573	-0.73315	-0.8634	-0.38525	3	0.005705	Cluster 2	-0.686882213
Q9UBV7	B4GALT7	Beta-1,4-galactosyltransferase 7	-0.205645683	0.001420879	-0.57305	-0.81362	-0.5098	-0.40413	1	0.009229	Cluster 1	-0.575151039
O15258	RER1	Protein RER1	-0.333060913	8.44E-05	-1.20457	-1.01584	-0.34497	-0.20672	2	0.003998	Cluster 2	-0.693024187
Q9BW60	ELOVL1	Elongation of very long chain fatty acids protein 1	-0.249548664	0.000105164	-1.02993	-0.74424	-0.24356	-0.2431	2	0.004235	Cluster 1	-0.565205991
P51571	SSR4	Translocon-associated protein subunit delta	-0.252006308	2.72E-05	-0.90891	-0.84546	-0.66243	-0.36926	1	0.002821	Cluster 2	-0.696514489
Q15021	NCAPD2	Condensin complex subunit 1	0.250753599	0.000940208	0.970612	0.66035	0.028832	-0.03841	1	0.007715	Cluster 3	0.405345391
Q5T1J5	CHCHD2P9	Putative coiled-coil-helix-coiled-coil-helix domain-containing protein CHCHD2P9, mitochondrial	-0.362429127	0.001372143	-1.15003	-0.9651	-0.27726	0.258516	1	0.009056	Protein not	in STRING database
Q9UBQ0	VPS29	Vacuolar protein sorting-associated protein 29	0.196843927	5.72E-06	0.677348	0.57821	0.280467	0.046853	1	0.002382	Cluster 3	0.395719518
O76094	SRP72	Signal recognition particle subunit SRP72	0.179147386	1.04E-05	0.714313	0.480606	0.319371	0.046243	1	0.002382	Cluster 3	0.390133115
P50851	LRBA	Lipopolysaccharide-responsive and beige-like anchor protein	-0.216232149	5.36E-05	-0.78605	-0.7118	-0.43508	-0.27787	2	0.003537	Cluster 1	-0.552699244
Q92504	SLC39A7	Zinc transporter SLC39A7	-0.265776254	0.000216234	-1.08155	-0.79738	-0.63857	-0.29623	1	0.00516	Cluster 2	-0.703431489
P32969	RPL9	Ribosomal protein L9	0.231032306	0.000866811	0.908906	0.56394	0.186313	-0.10185	1	0.007531	Cluster 3	0.38932831
Q9NYC9	DNAH9	Dynein heavy chain 9, axonemal	-0.169594403	0.000350833	-0.69869	-0.59964	-0.49935	-0.4096	2	0.00573	Cluster 1	-0.551821084
Q93050-1	ATP6V0A1	V-type proton ATPase 116 kDa subunit a isoform 1	-0.215154934	0.000375515	-0.84017	-0.72082	-0.29867	-0.34754	1	0.005794	Cluster 1	-0.551800197
Q96G23	CERS2	Ceramide synthase 2	-0.189748842	4.02E-05	-0.83369	-0.61336	-0.38865	-0.36349	2	0.002886	Cluster 1	-0.549797229
O00400	SLC33A1	Acetyl-coenzyme A transporter 1	-0.31936473	9.98E-05	-1.21998	-0.98495	-0.32885	-0.28712	2	0.004169	Cluster 2	-0.70522412
P24390	KDELR1	ER lumen protein-retaining receptor 1	-0.424399735	0.001713297	-1.31353	-1.24894	-0.33089	0.071136	1	0.009874	Cluster 2	-0.705555214
P13929	ENO3	Beta-enolase	0.195477578	2.55E-05	0.659357	0.560706	0.317634	0.007606	1	0.002741	Cluster 3	0.38632573
Q53GQ0	HSD17B12	Very-long-chain 3-oxoacyl-CoA reductase	-0.212966461	0.000271275	-0.81815	-0.70047	-0.32328	-0.30466	1	0.005252	Cluster 1	-0.53663995
P19623	SRM	Spermidine synthase	0.215355702	0.000735348	0.842819	0.595216	-0.02379	0.03325	2	0.00712	Cluster 3	0.361874395
Q7L2H7	EIF3M	Eukaryotic translation initiation factor 3 subunit M	0.2290876	0.001503748	0.858219	0.554963	0.151432	-0.15712	1	0.009361	Cluster 3	0.351872393

Q9UI15	TAGLN3	Transgelin-3	0.215580212	0.000396002	0.911239	0.522013	0.018712	-0.0585	1	0.005808	Cluster 3	0.348365432
Q7Z2W9	MRPL21	Mitochondrial ribosomal protein L21	0.178413844	0.000132396	0.597857	0.540669	0.148092	0.051484	1	0.004534	Cluster 3	0.334525426
Q9NVI7-2	ATAD3A	ATPase family AAA domain-containing protein 3A	-0.286148216	0.000202256	-1.06889	-0.91723	-0.53334	-0.33676	1	0.005124	Cluster 2	-0.714055321
Q6DKJ4	NXN	Nucleoredoxin	0.173432184	0.001537006	0.721506	0.441189	0.102437	-0.00174	1	0.009427	Cluster 3	0.315847141
Q7Z2Y8	GVINP1	Interferon-induced very large GTPase 1	-0.34846661	2.98E-06	-1.30437	-1.06208	-0.69595	-0.29592	2	0.002382	Protein not in	STRING database
P43307	SSR1	Translocon-associated protein subunit alpha	-0.283729939	8.56E-06	-1.0637	-0.89273	-0.62091	-0.31077	2	0.002382	Cluster 2	-0.722027566
P06733	ENO1	Alpha-enolase	0.178064941	0.00031221	0.551544	0.512157	0.224603	-0.04624	1	0.005694	Cluster 3	0.310516364
Q9NWU5	MRPL22	Mitochondrial ribosomal protein L22	0.162219358	0.000389504	0.431842	0.536368	0.115933	0.032484	1	0.0058	Cluster 3	0.279156648
Q86Y82	STX12	Syntaxin-12	-0.299228383	4.23E-05	-1.1594	-0.9563	-0.42267	-0.37243	2	0.002985	Cluster 2	-0.727699348
Q9Y5J7	TIMM9	Mitochondrial import inner membrane translocase subunit Tim9	-0.185472853	0.001443087	-0.78029	-0.58682	-0.40258	-0.29494	1	0.009253	Cluster 1	-0.516157147
P54920	NAPA	Alpha-soluble NSF attachment protein	-0.220035195	0.000332709	-0.86426	-0.81692	-0.67673	-0.57891	4	0.005705	Cluster 2	-0.734203532
P38435	GGCX	Vitamin K-dependent gamma-carboxylase	-0.187016944	0.001557683	-0.7341	-0.67238	-0.21702	-0.403	1	0.009444	Cluster 1	-0.506625865
O60437	PPL	Periplakin	-0.310721278	0.00093442	-1.09469	-1.01122	-0.50589	-0.32536	1	0.007715	Cluster 2	-0.734290047
P98160	HSPG2	Basement membrane-specific heparan sulfate proteoglycan core protein	-0.282089185	0.000363603	-0.82779	-0.90906	-0.18412	-0.07883	1	0.005767	Cluster 1	-0.4999483
Q16563-2	SYPL1	Synaptophysin-like protein 1	-0.17713837	0.000175615	-0.68772	-0.63612	-0.2912	-0.38196	2	0.004823	Cluster 1	-0.499249039
P16435	POR	NADPH--cytochrome P450 reductase	-0.245751039	0.000556969	-1.00127	-0.85596	-0.54952	-0.53426	1	0.00664	Cluster 2	-0.735252051
Q15041	ARL6IP1	ADP-ribosylation factor-like protein 6-interacting protein 1	-0.238110496	0.001002297	-0.71802	-0.72311	-0.12546	0.015777	1	0.007832	Cluster 3	-0.387702715
Q969X5	ERGIC1	Endoplasmic reticulum-Golgi intermediate compartment protein 1	-0.296705753	0.001060877	-1.10005	-0.82488	-0.04118	-0.0036	2	0.007965	Cluster 1	-0.492425988
Q8WX94	NLRP7	NACHT, LRR and PYD domains-containing protein 7	-0.228641598	0.000831023	-0.78995	-0.73709	-0.21397	-0.19069	1	0.007439	Cluster 1	-0.482924322
P02545	LMNA	Prelamin-A/C	-0.328303064	5.54E-05	-1.23625	-0.98621	-0.52088	-0.23916	2	0.003542	Cluster 2	-0.745626301
Q9P0L0	VAPA	Vesicle-associated membrane protein-associated protein A	-0.223101415	0.000266147	-0.87037	-0.63727	-0.29308	-0.11287	1	0.005252	Cluster 1	-0.478399341
Q16625	OCLN	Occludin	-0.366575988	0.000390779	-1.41048	-1.05501	-0.34878	-0.16867	1	0.0058	Cluster 2	-0.74573428
Q9NX40	OCIAD1	OCIA domain-containing protein 1	-0.159577626	1.27E-05	-0.57718	-0.55231	-0.49211	-0.28117	1	0.002382	Cluster 1	-0.47569107
O94826	TOMM70	Mitochondrial import receptor subunit TOM70	-0.166910636	0.000186548	-0.61573	-0.56582	-0.41603	-0.26877	1	0.004847	Cluster 1	-0.46658649
Q15654	TRIP6	Thyroid receptor-interacting protein 6	-0.188310029	0.000636085	-0.69368	-0.62867	-0.25089	-0.25642	1	0.006846	Cluster 1	-0.45741302
O94919	ENDOD1	Endonuclease domain-containing 1 protein	-0.340642962	0.000151385	-1.23564	-1.05063	-0.44953	-0.25173	1	0.004658	Cluster 2	-0.746882001
Q5JPE7	NOMO2	Nodal modulator 2	-0.169285634	3.58E-05	-0.59491	-0.57016	-0.27331	-0.21826	1	0.00286	Cluster 3	-0.414161283
Q13277	STX3	Syntaxin-3	-0.37444279	0.000340435	-1.4484	-1.08487	-0.28231	-0.18776	2	0.005705	Cluster 2	-0.750833843
P43005	SLC1A1	Excitatory amino acid transporter 3	-0.35459962	1.87E-05	-1.39629	-0.97892	-0.53238	-0.12422	2	0.002708	Cluster 2	-0.757951988
P11234	RALB	Ras-related protein Ral-B	-0.288469518	0.000602523	-1.07634	-0.91038	-0.7292	-0.32777	1	0.006731	Cluster 2	-0.760921161
Q86X29	LSR	Lipolysis-stimulated lipoprotein receptor	-0.295714413	0.001363575	-0.96478	-0.86057	-0.02759	0.044054	1	0.009029	Cluster 1	-0.45222244
P57735	RAB25	Ras-related protein Rab-25	-0.292471275	0.001197539	-1.10372	-0.99724	-0.4894	-0.48196	1	0.008402	Cluster 2	-0.768080115
Q9UM54-	MYO6	Unconventional myosin-VI	-0.218337152	8.50E-05	-0.75797	-0.66565	-0.14852	-0.09459	1	0.003998	Cluster 3	-0.41668506
O00468	AGRN	Agrin C-terminal 110 kDa subunit	-0.15393858	0.000907857	-0.5156	-0.58749	-0.30803	-0.33308	1	0.007644	Cluster 1	-0.436049908
Q96N66	MBOAT7	Lysophospholipid acyltransferase 7	-0.222711909	0.000151092	-0.79494	-0.67938	-0.12885	-0.11661	1	0.004658	Cluster 1	-0.429942789
P42858	HTT	Huntingtin, myristoylated N-terminal fragment	-0.148393716	0.000428718	-0.49951	-0.5324	-0.33762	-0.25267	1	0.006001	Cluster 1	-0.405548018
O75976	CPD	Carboxypeptidase D	-0.158537999	0.000117663	-0.58279	-0.53338	-0.24788	-0.22786	1	0.004443	Cluster 1	-0.397979165
Q969M3	YIPF5	Protein YIPF5	-0.200083545	0.000971693	-0.78042	-0.52871	0.078665	0.032007	1	0.007807	Cluster 1	-0.29961503
Q12797	ASPH	Aspartyl/asparaginyl beta-hydroxylase	-0.385907333	0.000753385	-1.54868	-1.15953	-0.13833	-0.32458	2	0.00712	Cluster 2	-0.792781444
P49590	HARS2	Histidine--tRNA ligase, mitochondrial	0.141154246	0.001069699	0.430771	0.469952	0.210047	0.102588	1	0.007998	Cluster 1	0.303339665
Q8TCT9	HM13	Minor histocompatibility antigen H13	-0.386361427	0.000510157	-1.37011	-1.22044	-0.37778	-0.3035	1	0.006524	Cluster 2	-0.817959282
Q15126	PMVK	Phosphomevalonate kinase	0.180266714	0.001637001	0.830416	0.418594	0.06473	-0.01482	1	0.009666	Cluster 1	0.32472962
Q96EK6	GNPNAT1	Glucosamine-phosphate N-acetyltransferase 1	0.208386221	0.000665503	0.818679	0.514692	0.082482	-0.0891	1	0.006907	Cluster 1	0.331686976
Q9BUF5	TUBB6	Tubulin beta-6 chain	0.205003042	0.000272063	0.90871	0.521632	0.187155	0.061436	1	0.005252	Cluster 1	0.419733032
O14744	PRMT5	Protein arginine N-methyltransferase 5, N-terminally processed	0.317251862	0.000828792	1.093549	0.88049	-0.09548	-0.09768	2	0.007437	Cluster 1	0.445217858
P16422	EPCAM	Epithelial cell adhesion molecule	-0.479282895	0.000440807	-1.69265	-1.37427	-0.18246	-0.02729	2	0.006052	Cluster 2	-0.819168033
Q9H173	SIL1	Nucleotide exchange factor SIL1	-0.255331024	0.001192813	-0.99379	-0.68639	-0.03426	0.002035	2	0.008401	Cluster 3	-0.428101258
P21246	PTN	Pleiotrophin	-0.42005689	0.00067238	-1.60197	-1.23213	-0.30806	-0.22036	1	0.006909	Cluster 2	-0.840627358
Q6V0I7	FAT4	Protocadherin Fat 4	-0.169513349	0.000889262	-0.84707	-0.47939	-0.14952	-0.25269	1	0.007615	Cluster 3	-0.432169125
P08047	SP1	Transcription factor Sp1	0.23239423	0.000338529	0.787411	0.675508	0.489042	0.044757	1	0.005705	Cluster 1	0.499179297
Q15813	TBCE	Tubulin-specific chaperone E	0.367902118	0.001534211	1.276819	0.956808	0.010927	-0.23387	2	0.009427	Cluster 1	0.502671166

Q9BTE6	AARSD1	Alanyl-tRNA editing protein Aarsd1	0.362049149	0.001402968	1.266793	0.97094	-0.00592	-0.15648	2	0.009171	Cluster 1	0.518832844
O95471	CLDN7	Claudin-7	-0.40535791	0.000715889	-1.61204	-1.26678	-0.15707	-0.43603	1	0.007112	Cluster 2	-0.867981992
O14530	TXNDC9	Thioredoxin domain-containing protein 9	0.280756811	0.000220066	1.072706	0.747342	0.282874	-0.01148	2	0.00516	Cluster 1	0.522861758
Q56VL3	OCIAD2	OCIA domain containing 2	-0.419243183	0.0005556	-1.49305	-1.31886	-0.35978	-0.31798	2	0.00664	Cluster 2	-0.872419104
P15291	B4GALT1	Processed beta-1,4-galactosyltransferase 1	-0.389680088	5.99E-05	-1.44305	-1.23742	-0.46134	-0.39063	2	0.003542	Cluster 2	-0.883109885
P61619	SEC61A1	Protein transport protein Sec61 subunit alpha isoform 1	-0.192057352	9.19E-05	-0.69597	-0.62924	-0.46562	-0.24677	1	0.004014	Cluster 3	-0.509399667
P49189	ALDH9A1	4-trimethylaminobutyraldehyde dehydrogenase, N-terminally processed	0.346828934	0.000399807	1.161604	0.947005	0.144784	-0.14761	2	0.005822	Cluster 1	0.526446486
O95671	ASMTL	Probable bifunctional dTTP/UTP pyrophosphatase/methyltransferase protein	0.305722563	0.000744202	1.110738	0.875513	0.109514	0.045235	1	0.00712	Cluster 1	0.535250209
Q9BTU6	PI4K2A	Phosphatidylinositol 4-kinase type 2-alpha	-0.253144023	0.000934221	-0.92223	-0.85211	-0.0094	-0.31355	1	0.007715	Cluster 3	-0.524325321
Q14108	SCARB2	Lysosome membrane protein 2	-0.4426767	0.00126174	-1.75531	-1.37521	0.006633	-0.43302	2	0.008707	Cluster 2	-0.889227124
O60502	OGA	Protein O-GlcNAcase	0.196643619	6.58E-06	0.738395	0.659578	0.469667	0.311722	1	0.002382	Cluster 1	0.544840242
Q96KC8	DNAJC1	DnaJ homolog subfamily C member 1	-0.230263848	2.79E-05	-0.9688	-0.66709	-0.26625	-0.20375	2	0.002821	Cluster 3	-0.526470536
Q8NE86	MCU	Calcium uniporter protein, mitochondrial	-0.368864201	6.73E-05	-1.39815	-1.18276	-0.65558	-0.4517	2	0.003542	Cluster 2	-0.922047588
Q9NRG0	CHRAC1	Chromatin accessibility complex protein 1	0.29408255	0.000237137	1.172671	0.763965	0.255975	-0.00934	2	0.005174	Cluster 1	0.545818458
Q02818	NUCB1	Nucleobindin-1	-0.284298834	0.000224441	-1.03516	-0.75725	-0.40862	0.048064	2	0.00516	Cluster 3	-0.538240754
O43760	SYNGR2	Synaptogyrin-2	-0.195337762	6.03E-05	-0.82384	-0.63743	-0.35415	-0.34801	1	0.003542	Cluster 3	-0.54085576
Q96AB3	ISOC2	Isochorismatase domain containing 2	0.160431251	0.001717989	0.619766	0.626207	0.519806	0.482607	1	0.009874	Cluster 1	0.562096298
Q3KQU3	MAP7D1	MAP7 domain containing 1	0.199915473	2.93E-05	0.697442	0.716195	0.520365	0.369863	1	0.002821	Cluster 1	0.575966204
P61803	DAD1	Dolichyl-diphosphooligosaccharide--protein glycosyltransferase subunit DAD1	-0.459963586	9.47E-05	-1.65469	-1.3757	-0.45146	-0.21343	2	0.004035	Cluster 2	-0.923816964
Q8NFF5	FLAD1	Molybdenum cofactor biosynthesis protein-like region	0.233752554	3.77E-05	0.873059	0.736414	0.458062	0.248877	1	0.00286	Cluster 1	0.579103098
Q9UNL2	SSR3	Translocon-associated protein subunit gamma	-0.399071273	5.78E-06	-1.53277	-1.17011	-0.78837	-0.27424	2	0.002382	Cluster 2	-0.941371087
P53007	SLC25A1	Tricarboxylate transport protein, mitochondrial	-0.393792728	0.0004817	-1.51538	-1.25246	-0.75928	-0.48882	1	0.006246	Cluster 2	-1.003985307
Q05209	PTPN12	Tyrosine-protein phosphatase non-receptor type 12	0.343863092	0.000778754	1.313694	0.896409	0.221944	-0.07005	1	0.007263	Cluster 1	0.590499386
Q99805	TM9SF2	Transmembrane 9 superfamily member 2	-0.235105278	2.90E-05	-0.86158	-0.74859	-0.34773	-0.23881	2	0.002821	Cluster 3	-0.549174214
P59768	GNG2	Guanine nucleotide-binding protein G(I)/G(S)/G(O) subunit gamma-2	0.349979628	0.000553149	1.29042	0.930425	0.223308	-0.07807	2	0.00664	Cluster 1	0.591521308
P04406	GAPDH	Glyceraldehyde-3-phosphate dehydrogenase	0.338594382	0.000241492	1.302167	0.931385	0.175566	0.043097	2	0.005174	Cluster 1	0.613053727
Q96HV5	TMEM41A	Transmembrane protein 41A	-0.305598348	6.84E-05	-1.27335	-1.09306	-0.87279	-0.77733	4	0.003542	Cluster 2	-1.004132324
Q9Y5M8	SRPRB	Signal recognition particle receptor subunit beta	-0.184599951	0.000358005	-0.70587	-0.62421	-0.57977	-0.33194	1	0.005767	Cluster 3	-0.560446879
Q2M2H8	MGAM2	Maltase-glucoamylase 2	-0.735203027	0.001512478	-2.37267	-2.09825	-0.02105	0.232556	1	0.009377	Cluster 2	-1.064853023
Q1HG44	DUOXA2	Dual oxidase maturation factor 2	-0.536424521	0.001160128	-2.08169	-1.6589	-0.2279	-0.48882	1	0.00827	Cluster 2	-1.114329602
Q9BRK5	SDF4	45 kDa calcium-binding protein	-0.338790294	0.000647438	-1.20245	-0.94151	-0.1976	0.033608	2	0.006846	Cluster 3	-0.576988296
P04843	RPN1	Dolichyl-diphosphooligosaccharide--protein glycosyltransferase subunit 1	-0.22011139	2.28E-05	-0.86486	-0.68651	-0.51667	-0.27119	1	0.002708	Cluster 3	-0.584806504
Q9HD45	TM9SF3	Transmembrane 9 superfamily member 3	-0.211000846	9.29E-06	-0.81193	-0.71801	-0.50838	-0.37744	2	0.002382	Cluster 3	-0.60394163
O95202	LETM1	Mitochondrial proton/calcium exchanger protein	-0.200854761	0.000356037	-0.80473	-0.69076	-0.51139	-0.41035	1	0.005759	Cluster 3	-0.604310883
Q7Z7N9	TMEM179	Transmembrane protein 179B	-0.340272461	0.001015555	-1.30824	-0.9939	0.006656	-0.15126	2	0.00785	Cluster 3	-0.61168862
Q9BQA1	WDR77	Methylosome protein 50	0.352628458	0.000429724	1.298093	0.97011	0.1998	-0.01101	2	0.006001	Cluster 1	0.614248594
Q9Y365	STARD10	START domain-containing protein 10	-0.393806705	0.001021295	-1.33439	-1.24382	0.122875	-0.19048	1	0.007851	Cluster 3	-0.661455268
P46977	STT3A	Dolichyl-diphosphooligosaccharide--protein glycosyltransferase subunit STT3A	-0.296772463	2.47E-05	-1.08275	-0.92781	-0.4735	-0.26241	2	0.002741	Cluster 3	-0.686618151
P37059	HSD17B2	Estradiol 17-beta-dehydrogenase 2	-0.407291683	0.001734107	-1.85026	-1.35599	-0.38025	-0.87815	3	0.009932	Cluster 2	-1.116163187
P05783	KRT18	Keratin, type I cytoskeletal 18	-0.675807921	0.000940212	-2.25552	-1.96393	-0.41772	0.016774	1	0.007715	Cluster 2	-1.155098497
Q15063-5	POSTN	Periostin	0.367341687	0.000118363	1.310618	1.013204	0.215618	-0.04646	2	0.004443	Cluster 1	0.623244766
Q9BXK5	BCL2L13	Bcl-2-like protein 13	-0.244873512	0.001076514	-1.03501	-0.80894	-0.44987	-0.4611	2	0.007998	Cluster 3	-0.688728581
Q9UBY8	CLN8	Protein CLN8	-0.336665416	0.001559537	-1.12903	-1.1063	-0.24122	-0.28502	1	0.009444	Cluster 3	-0.690391369
Q16555	DPYSL2	Dihydropyrimidinase-related protein 2	0.352763492	0.001320961	1.34172	1.034623	-0.03037	0.14907	1	0.00892	Cluster 1	0.623760538
Q9NUN5	LMBRD1	Probable lysosomal cobalamin transporter	-0.306815168	0.000218122	-1.08363	-1.02865	-0.36076	-0.37554	1	0.00516	Cluster 3	-0.712143303
Q9UHQ9	CYB5R1	NADH-cytochrome b5 reductase 1	-0.526413473	0.000322967	-2.00813	-1.66039	-0.48446	-0.54607	2	0.005705	Cluster 2	-1.174763685
Q96GM5	SMARCD1	SWI/SNF-related matrix-associated actin-dependent regulator of chromatin subfamily D member 1	0.421156889	0.00149495	1.472735	1.130452	0.128623	-0.16563	2	0.009361	Cluster 1	0.641544335
Q13885	TUBB2A	Tubulin beta-2A chain	0.382961199	0.001055338	1.598913	0.935649	0.126757	-0.09484	2	0.007965	Cluster 1	0.641619048
Q9UM00	TMCO1	Calcium load-activated calcium channel	-0.292089384	0.000342458	-1.16446	-0.93695	-0.51401	-0.41524	1	0.005705	Cluster 3	-0.757665808
Q9NPR9	GPR108	G protein-coupled receptor 108	-0.371215079	0.000649145	-1.39977	-1.1849	-0.08945	-0.37191	2	0.006846	Cluster 3	-0.76150686
Q9Y314	NOSIP	Nitric oxide synthase-interacting protein	0.350524192	0.000408826	1.285352	0.989372	0.295898	0.050539	2	0.005891	Cluster 1	0.655289978
Q9UBX3	SLC25A10	Mitochondrial dicarboxylate carrier	-0.170301823	0.000322208	-0.61298	-0.59073	-0.24847	-0.26922	1	0.005705	Protein is not part of any cluster	
Q6ZP65	BICDL1	BICD family-like cargo adapter 1	-0.327765989	6.44E-05	-1.24327	-0.95662	-0.69149	-0.20468	2	0.003542	Cluster 3	-0.774016844
Q16658	FSCN1	Fascin	0.299800185	1.14E-05	1.124629	0.923735	0.345678	0.251091	2	0.002382	Cluster 1	0.661283025
Q86TM6	SYVN1	E3 ubiquitin-protein ligase synoviolin	-0.330057106	1.10E-05	-1.3147	-0.98608	-0.62655	-0.31169	2	0.002382	Cluster 3	-0.809754243
Q6PIU2	NCEH1	Neutral cholesterol ester hydrolase 1	-0.441631118	0.000299715	-1.5999	-1.5383	-0.9409	-0.75565	2	0.0056	Cluster 2	-1.208688271
P14060	HSD3B1	3 beta-hydroxysteroid dehydrogenase/Delta 5-->4-isomerase type 1	-0.71586054	0.00039145	-2.4067	-2.13157	-0.36924	-0.10548	2	0.0058	Cluster 2	-1.253246449
Q8NEW0	SLC30A7	Zinc transporter 7	-0.407592701	0.000667168	-1.46993	-1.08775	-0.75522	0.05957	2	0.006907	Cluster 3	-0.813333723
P05787	KRT8	Keratin, type II cytoskeletal 8	-0.718793871	0.000779186	-2.46876	-2.12308	-0.46953	-0.13082	1	0.007263	Cluster 2	-1.298046094

Q96LZ7	RMDN2	Regulator of microtubule dynamics 2	-0.343678116	0.000347731	-1.26241	-1.21863	-0.33758	-0.60938	3	0.005728	Cluster 3	-0.857000402
Q99571	P2RX4	P2X purinoceptor 4	-0.558813398	0.000580254	-2.19951	-1.65236	0.191818	-0.32408	2	0.006707	Cluster 3	-0.996034054
Q13286	CLN3	Battenin	-0.525373825	0.001705867	-1.97697	-1.78347	-0.67293	-0.81963	1	0.009859	Cluster 2	-1.3132495
P07333	CSF1R	Macrophage colony-stimulating factor 1 receptor	-0.726239013	0.000114023	-2.63725	-2.02755	-0.77689	-0.04753	2	0.004443	Cluster 2	-1.372303254
P0DJJ0	SRGAP2C	SLIT-ROBO Rho GTPase-activating protein 2C	0.38465523	0.000944591	1.407452	1.036007	0.314476	-0.05918	2	0.007715	Cluster 1	0.674688565
P19012	KRT15	Keratin, type I cytoskeletal 15	-0.615390351	0.000297057	-2.32786	-1.98101	-0.96121	-0.75138	1	0.005576	Cluster 2	-1.505364127
Q9NVW2	RLIM	E3 ubiquitin-protein ligase RLIM	-0.692148667	0.000812938	-2.36608	-1.98175	-0.33274	0.037637	1	0.007396	Cluster 3	-1.160734104
Q9Y5Q8	GTF3C5	General transcription factor 3C polypeptide 5	0.483137045	0.00072881	1.6509	1.247392	0.229822	-0.32927	2	0.00712	Cluster 1	0.699711258
Q709F0	ACAD11	Acyl-CoA dehydrogenase family member 11	-0.472447568	0.000759601	-1.88073	-1.60564	-0.51418	-0.83397	2	0.007162	Cluster 3	-1.208630586
P08727	KRT19	Keratin, type I cytoskeletal 19	-0.717632167	0.0002446	-2.56283	-2.22311	-0.75816	-0.49073	1	0.005213	Cluster 2	-1.508705883
Q9UHD2	TBK1	Serine/threonine-protein kinase TBK1	-0.591776096	0.001623385	-2.50723	-1.90957	-0.91576	-1.00045	1	0.009613	Cluster 2	-1.583251059
Q13509	TUBB3	Tubulin beta-3 chain	0.343737359	0.000539517	1.699836	0.970437	0.477184	0.510202	2	0.006627	Cluster 1	0.914414615
P08729	KRT7	Keratin, type II cytoskeletal 7	-0.689535864	0.000871646	-2.59922	-2.3964	-0.78064	-1.19255	1	0.007557	Cluster 2	-1.742201966
Q92508	PIEZO1	Piezo-type mechanosensitive ion channel component 1	-0.664308811	0.000424136	-2.03076	-1.95777	-1.36885	0.035834	2	0.006001	Cluster 3	-1.330383601
Q9BVA1	TUBB2B	Tubulin beta-2B chain	0.624279425	0.001016797	2.314391	1.634082	0.012088	-0.2264	2	0.00785	Cluster 1	0.933539355
Q9Y592	CEP83	Centrosomal protein of 83 kDa	-0.992400578	0.00042139	-4.0305	-2.93619	-0.2449	-0.76885	2	0.005987	Cluster 3	-1.995109904
Q96P66	GPR101	Probable G-protein coupled receptor 101	0.494300065	0.001084452	1.724625	1.421086	0.765019	0.079517	2	0.007998	Cluster 1	0.997561579
Q9C0A0	CNTNAP4	Contactin-associated protein-like 4	0.473106242	0.000143203	1.885574	1.535457	0.684155	0.695224	2	0.004614	Cluster 1	1.200102403
