# Supplementary material for: Hepatocyte-intrinsic SMN deficiency drives metabolic dysfunction and liver steatosis in spinal muscular atrophy
Source: J Clin Invest. 2024 May 9;134(12):e173702. doi: 10.1172/JCI173702 (PMC11178536; doi:10.1172/JCI173702)
Supplement: Unedited blot and gel images [file jci-134-173702-s126.pdf]

Figure 1A

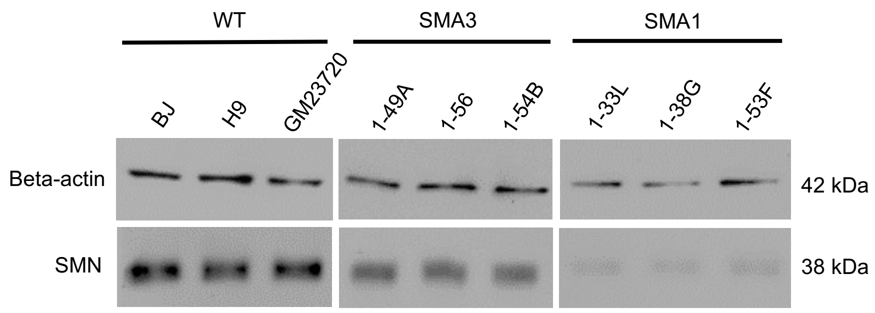
WT
SMA3
SMA1
BJ
H9
GM23720
1-49A
1-56
1-54B
1-33L
1-38G
1-53F
Beta-actin
SMN
42 kDa
38 kDa

Unedited blot for figure 1A

Beta-actin

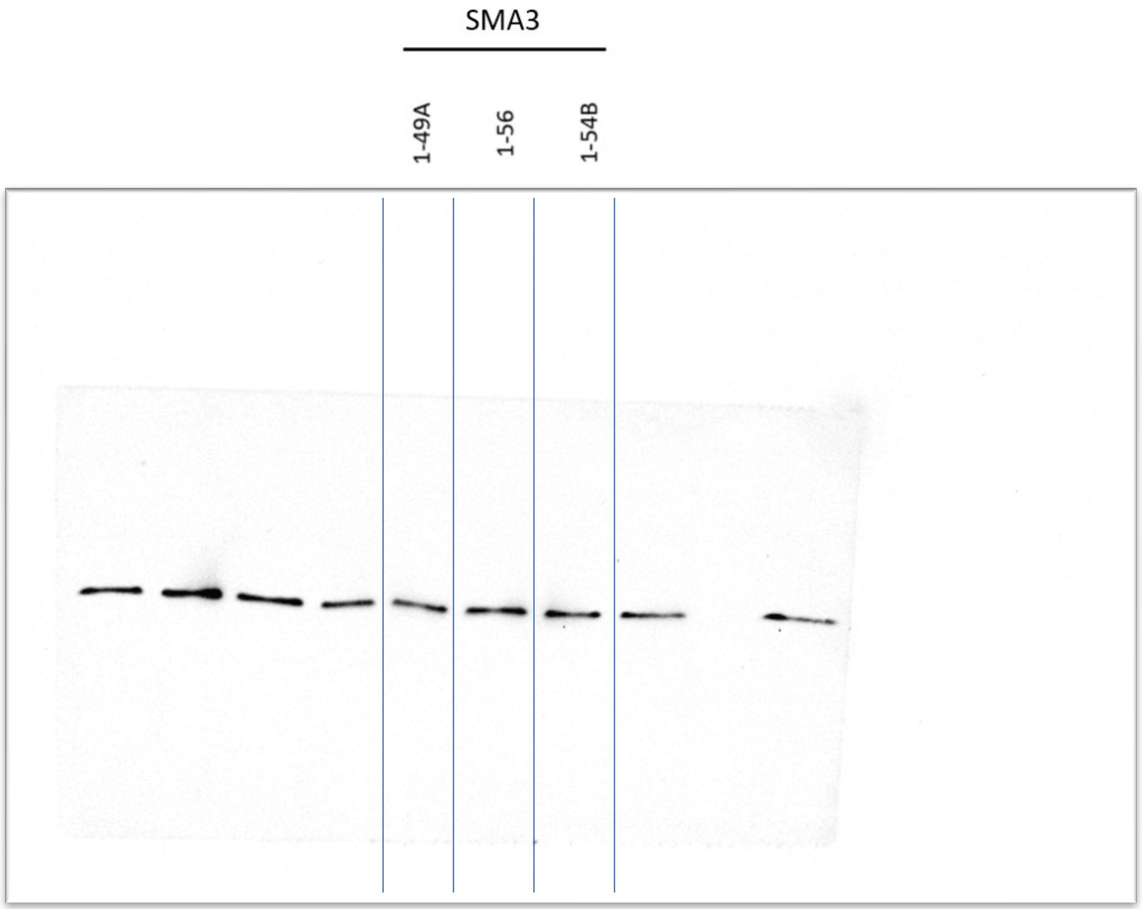

Blot 1

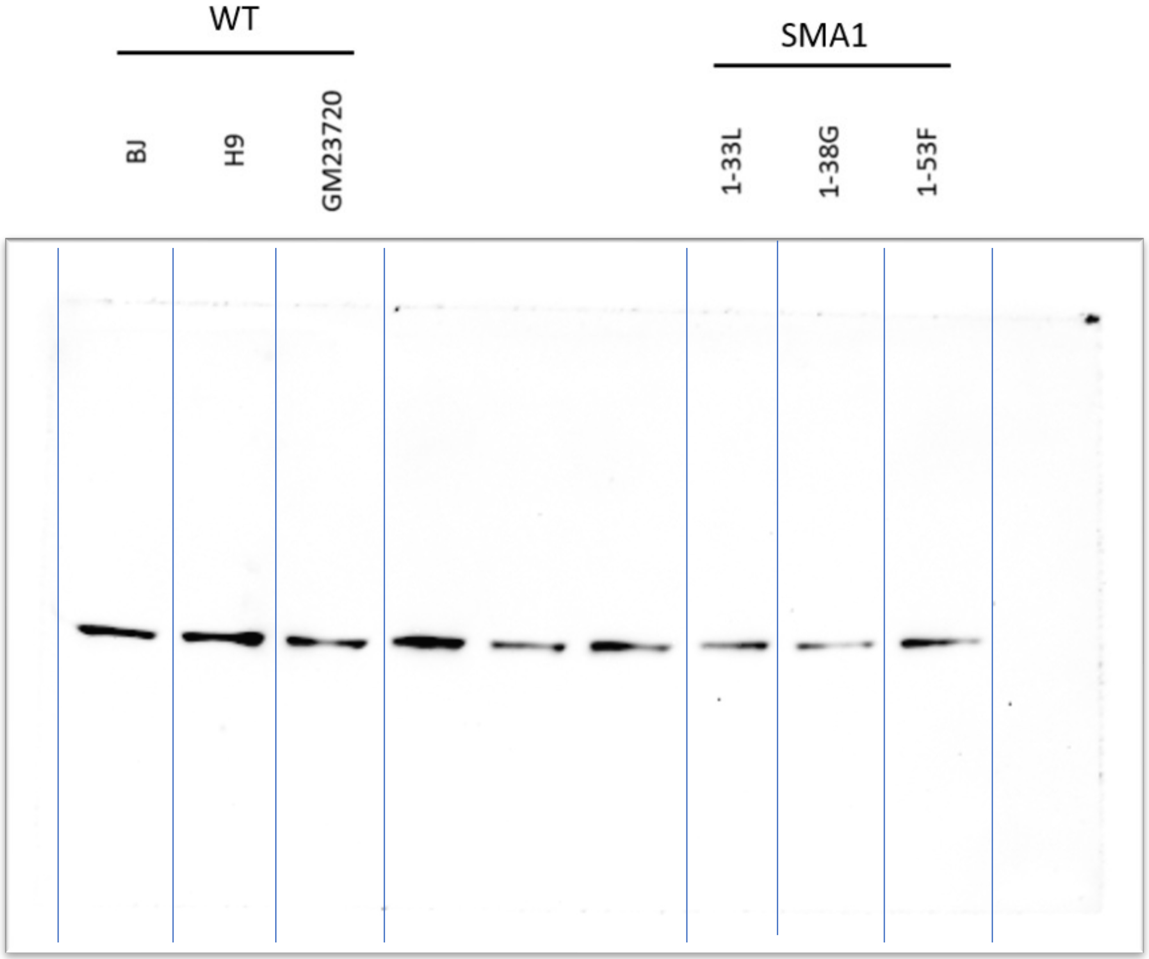

Blot 2

Unedited blot for figure 1A

SMN

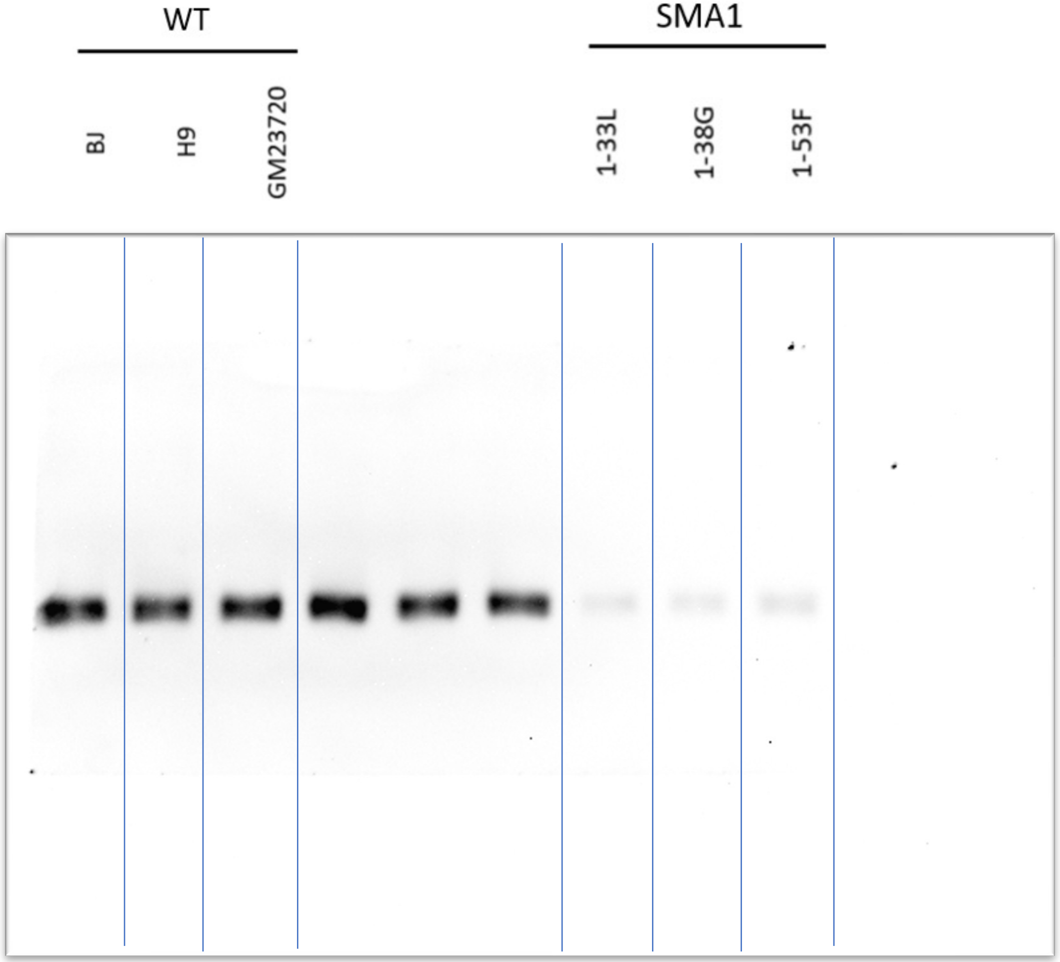

Blot 3

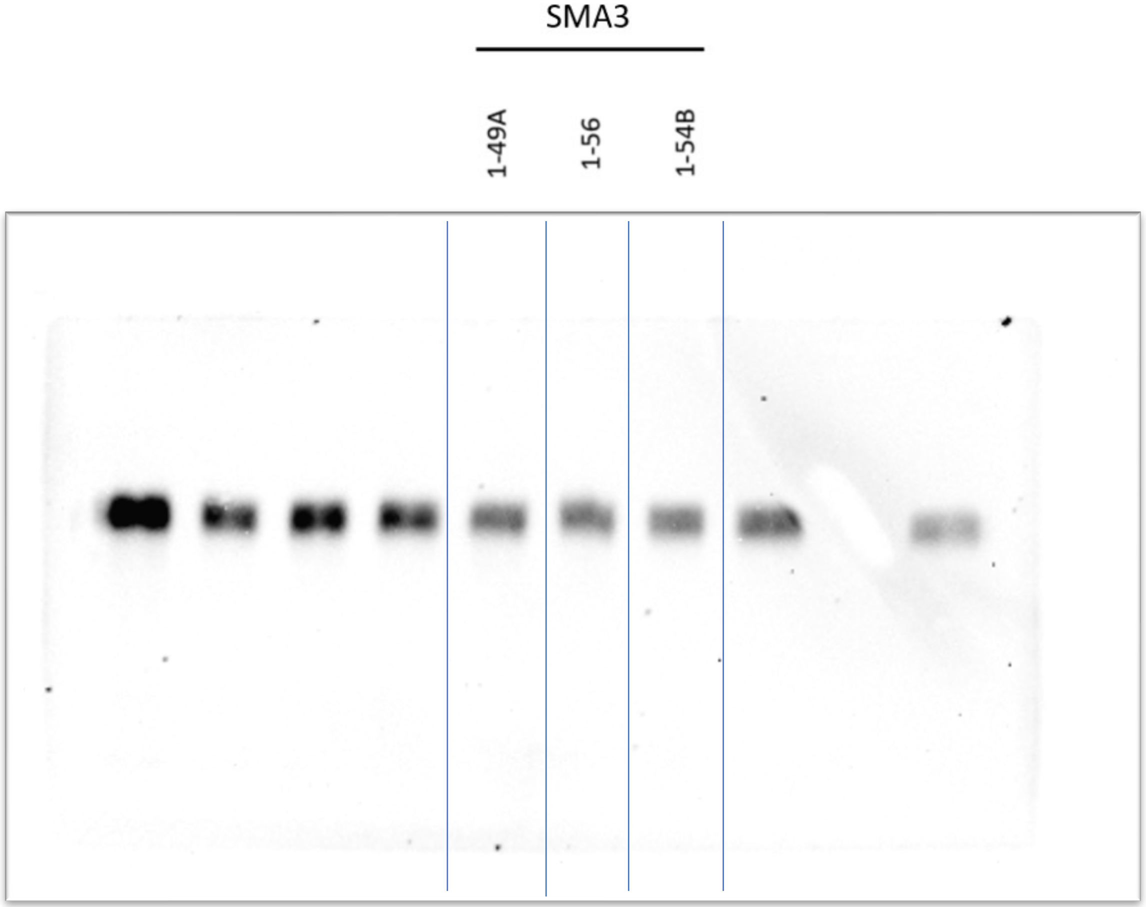

Blot 4

Supplemental Figure 14B

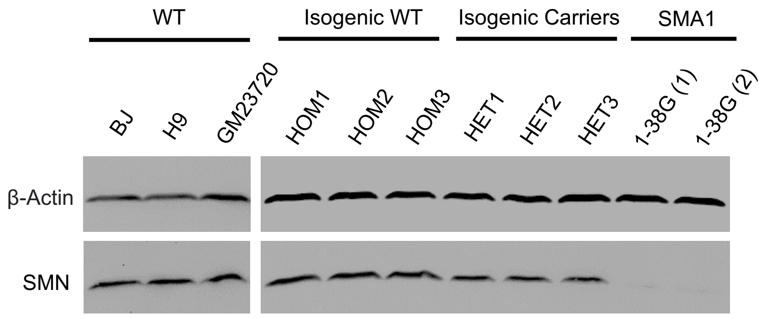

Unedited blot for supplemental figure 14B

Beta actin

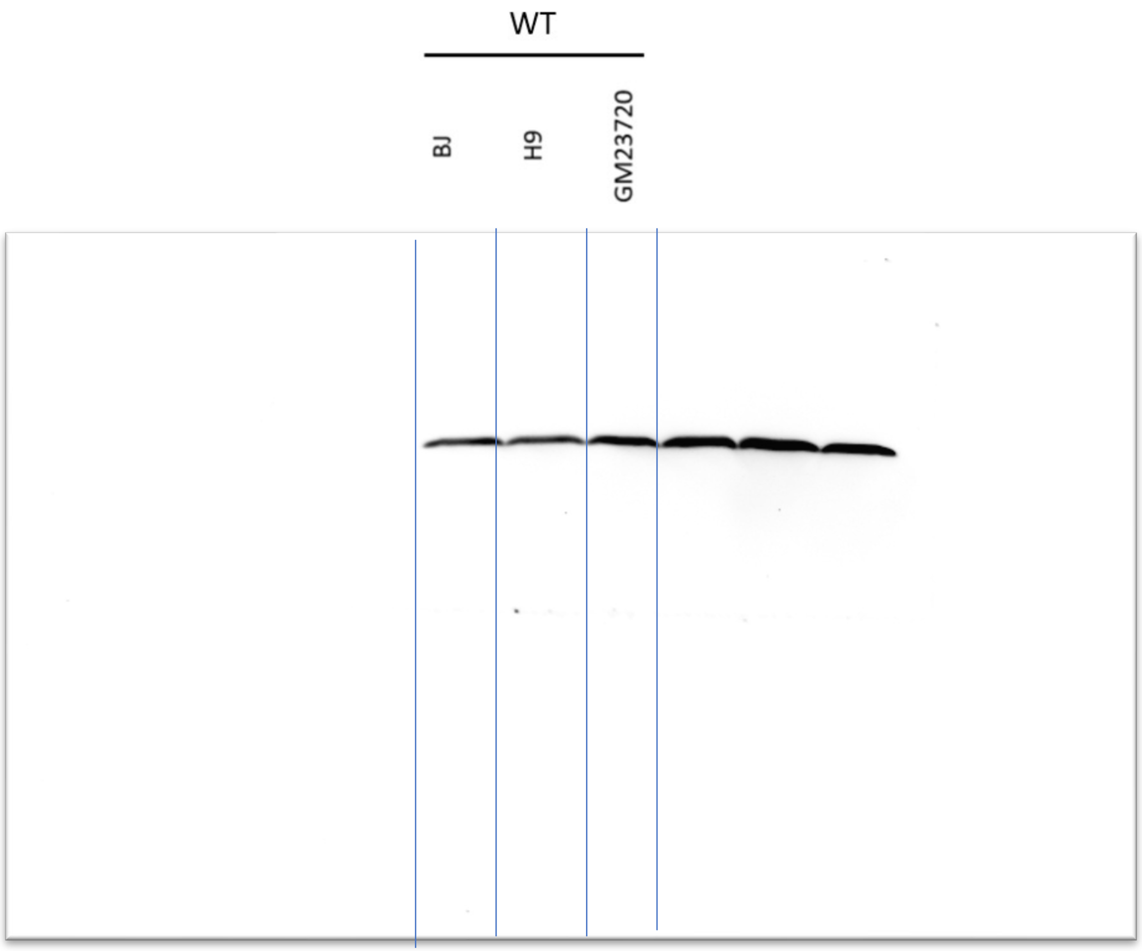

Blot 5

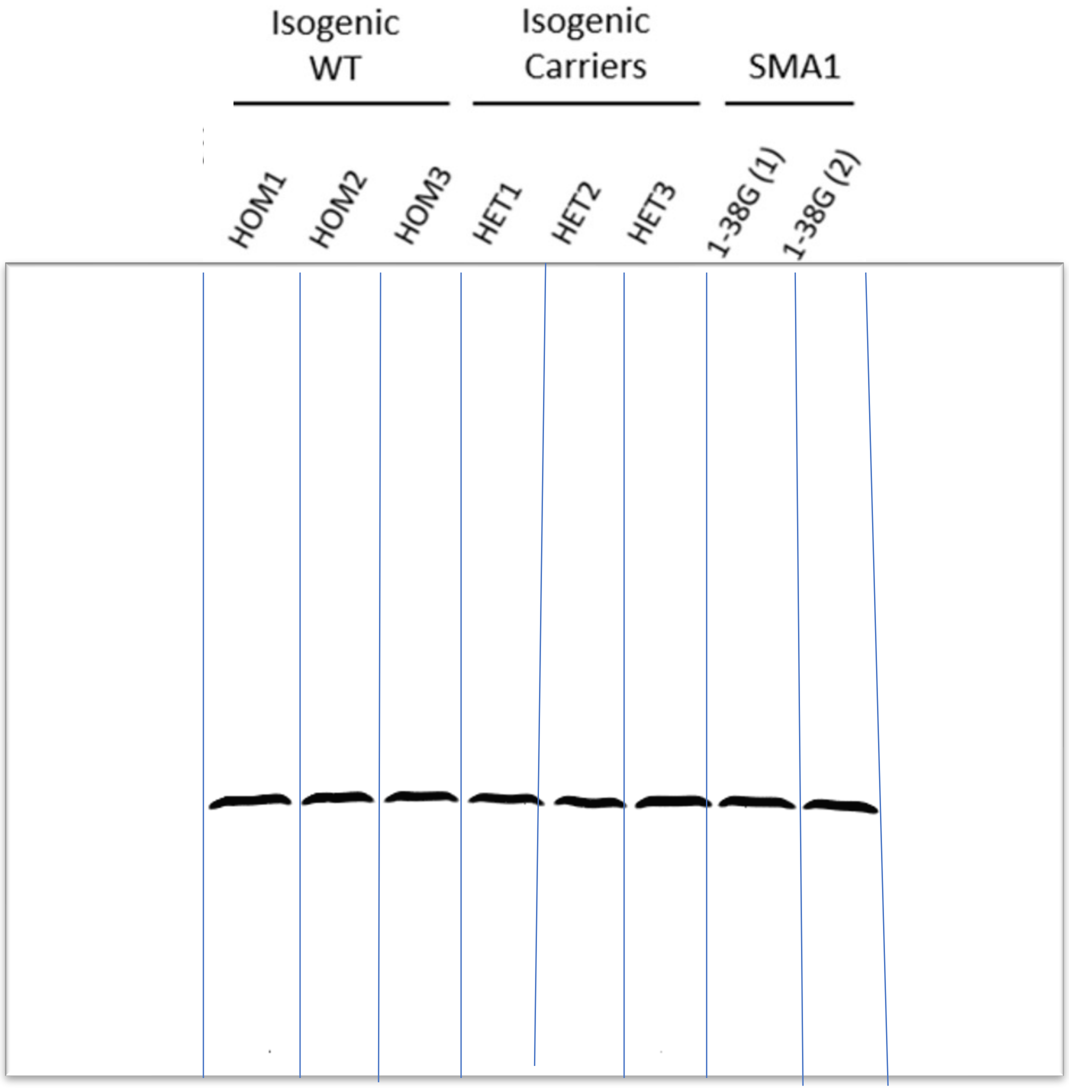

Blot 6

Unedited blot for supplemental figure 14B

SMN

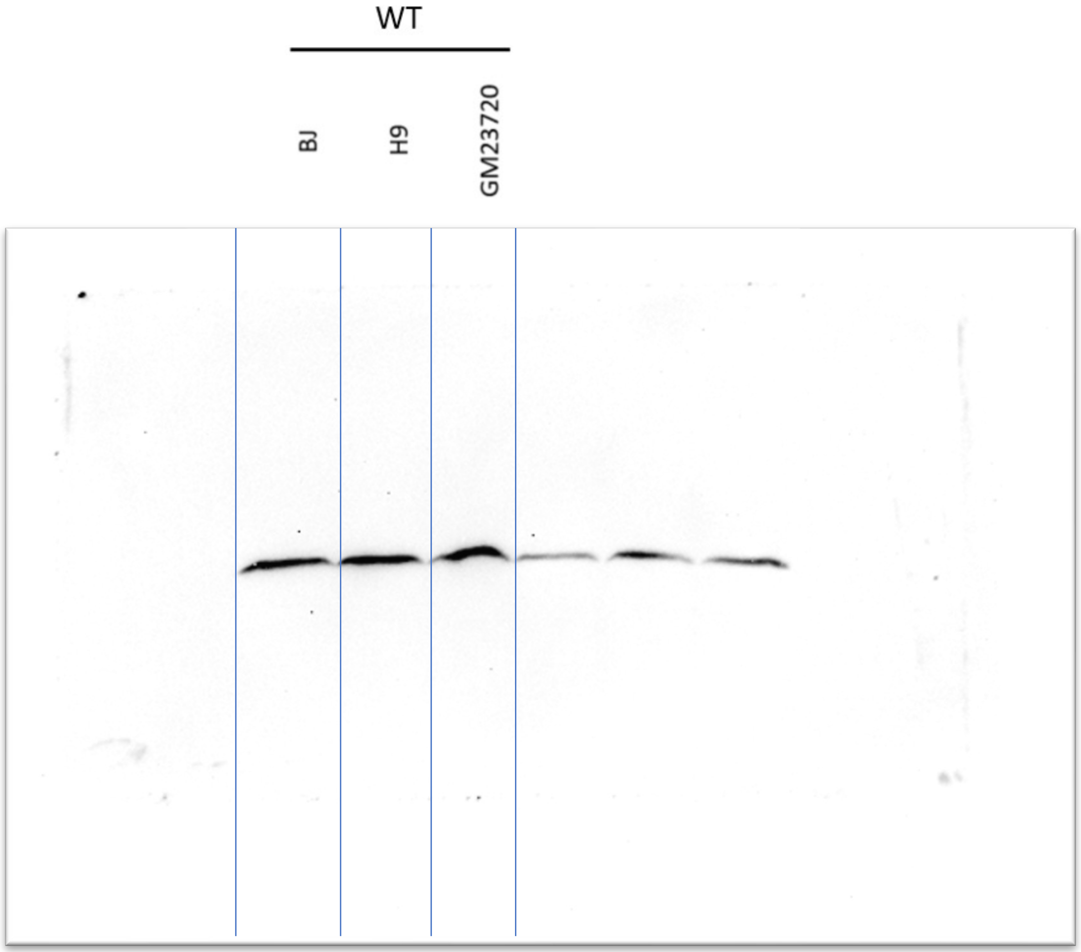

Blot 7

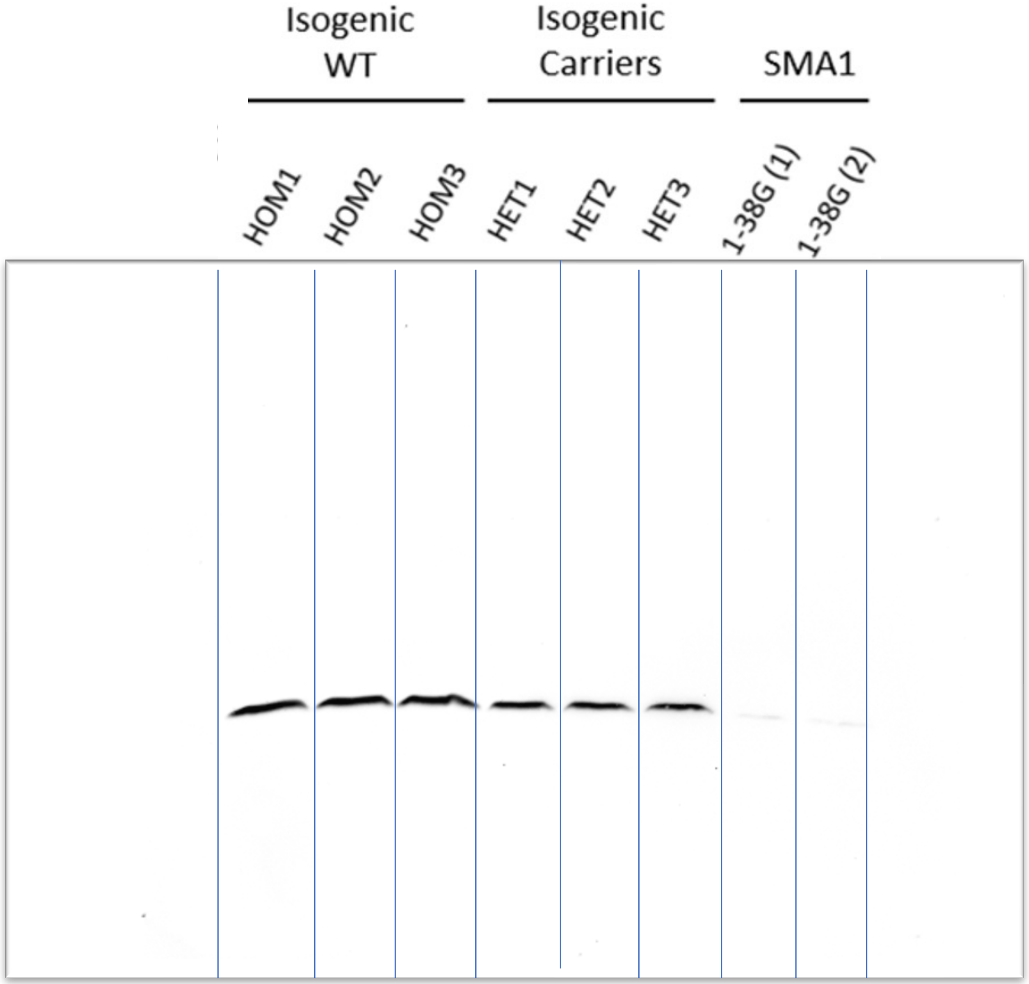

Blot 8
